# Alkoxide-induced ring opening of bicyclic 2-vinylcyclobutanones: A convenient synthesis of 2-vinyl-substituted 3-cycloalkene-1-carboxylic acid esters

**DOI:** 10.3762/bjoc.8.72

**Published:** 2012-04-26

**Authors:** Xiufang Ji, Zhiming Li, Quanrui Wang, Andreas Goeke

**Affiliations:** 1 Department of Chemistry, Fudan University, 220 Handan Road, 200433 Shanghai, P. R. China; 2 Shanghai Givaudan Ltd., Fragrances, 298 Li Shi Zhen Road, 201203 Shanghai, P. R. China

**Keywords:** alkoxide, cyclobutanones, esters, fused ring systems, ring opening

## Abstract

The fused 2-vinyl or 2-phenyl substituted cyclobutanones **4** undergo stereoselective ring openings by the action of alkoxide ions (*t-*BuO^−^ or MeO^−^) to produce novel vicinally disubstituted cycloalkene derivatives **5** and **6** in moderate to high yields. The ring cleavage usually occurs with complete regioselectivity. The accessibility of γ,δ-unsaturated ester or acid derivatives makes this transformation a good supplementary method for the well-established Johnson–Claisen rearrangement.

## Introduction

A great variety of methods are available for the synthesis of cyclobutane derivatives. Frequently employed ones include the thermal [2 + 2] cycloaddition of ketenes to alkenes and the polar addition of cyclopropyl ylides to carbonyls [1–2]. These methods generally allow regioselective as well as stereoselective syntheses of extensively substituted four-membered ring carbocycles. In particular, due to their facile accessibility [3–8] and expeditious ring transformations, the use of cyclobutanones as extremely versatile and useful starting materials or intermediates in the construction of carbon skeletons has flourished over the past three decades [9–10]. In addition, the high electrophilicity of the carbonyl carbon atom, and the puckering of the cyclobutane caused by the substitution at C-2 and C-4, also offer both interesting preparative and mechanistic aspects [11]. The ease of ring opening of cyclobutanones is influenced by the strain of the four-membered rings, the substitution pattern on the ring, and the properties of the reagents as well as the reaction conditions. In particular, the ring opening of certain fused-ring cyclobutanones, which are easily prepared by the well-established [2 + 2] cycloadditions of ketenes with various cycloalkenes, has been extensively investigated [3–8,12–14]. To achieve the ring opening, many reaction conditions have been investigated, such as acidic conditions, basic conditions, nucleophilic attack, thermolysis, and oxidizing as well as reducing conditions [4,11]. Cohen and Matz described the [1,2]- and [1,3]-acyl migration of 2-vinylcyclobutanones, leading to cyclopentenones or cyclohexenones, respectively [15]. Danheiser and co-workers reported an oxyanion-accelerated [1,3]-rearrangement of in situ reduced 2-vinylcyclobutanones, by applying mixtures of LiBu_3_BH-MeLi and HMPT to give bicyclic cyclohexenols [16], which were also subsequently described by Cohen who elaborated the method into a synthesis of (−)-β-selinene [17]. The core structures of ophiobolins A and other natural products can be synthesized by an alkenylation/oxy-Cope/methylation sequence of vinylcyclobutones in one pot [18–23]. Ring-opening reactions of cyclobutanones are expected to be a valuable methodology for vicinal difunctionalization of double bonds. A prerequisite for this reaction to take place is the presence at C-2 of substituents capable of stabilizing the developing vicinal carbanion [6–8]. In this context, α,α-dihalosubstituents, thioketal, sulfoxide, ester or aryl substituents have been reported to facilitate the ring cleavage [6–8].

**Scheme 1:**
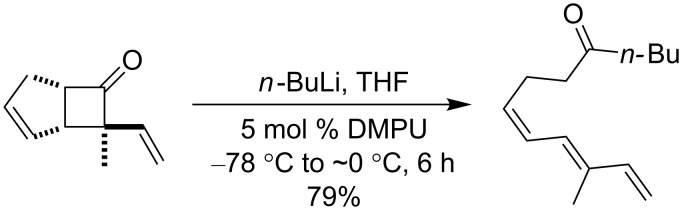
Metathetic ring opening of 7-methyl-7-vinylbicyclo[3.2.0]hept-2-en-6-one to a linear polyene ketone.

We recently reported a novel metathetic cycloreversion of 7-vinyl and aryl bicyclo[3.2.0]hept-2-en-6-ones giving rise to linear polyene ketones. The reaction was triggered by an in situ alkylation with alkyl or aryllithium reagents in the presence of catalytic amounts of Li-chelating agents (e.g., Scheme 1) [24]. The configuration of the newly formed double bond in position 6 was diastereoselectively determined by the configuration of the bicyclic precursor.

Our ongoing interest in constructing odorants from easily accessible starting materials prompted us to search further for novel transformations of cyclobutanones. We were pleased to find that selective ring cleavage of bicyclic cyclobutanones **4** bearing a vinyl or phenyl group in the alpha position can be achieved by the action of sodium methoxide or potassium *tert*-butoxide. This approach constitutes an efficient path to vicinally disubstituted ester products **5** or **6**.

## Results and Discussion

**Scheme 2:**
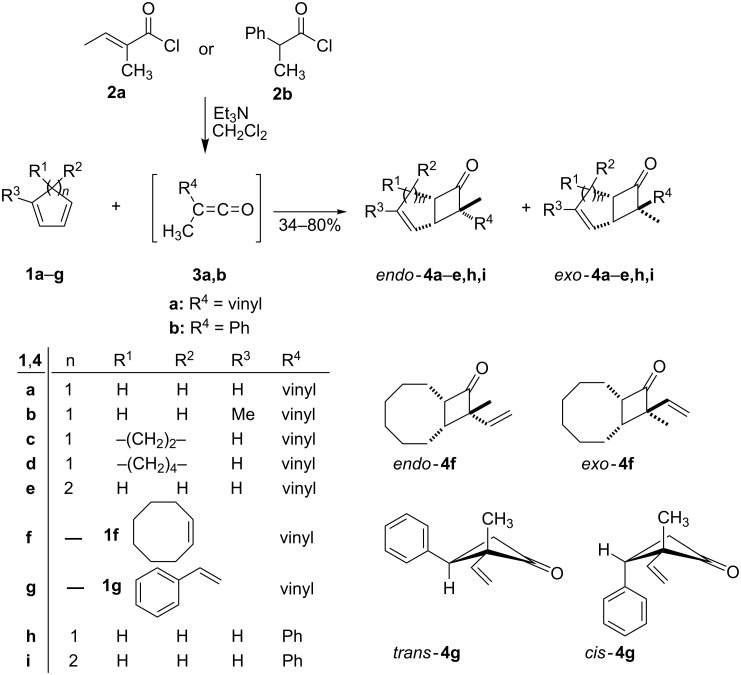
Synthesis of vinyl or phenyl substituted cyclobutanones **4a**–**i**.

We began our investigation with the preparation of cyclobutanones as shown in Scheme 2. The reaction involves generation of the intermediate vinylketenes or phenylketenes by the triethylamine-promoted 1,4- or 1,2-dehydrochlorination of acyl chlorides **2** [1]. The in situ generated vinylketenes or phenylketenes **3** were trapped by reaction with excessive amount of alkenes **1** in methylene chloride. The reaction of ketenes (R_2_C=C=O) with alkenes under thermal conditions to give cyclobutanone products is generally described as an [_π_2_s_ + _π_2_a_] process from orbital symmetry considerations. However, there is strong evidence that formation of the Staudinger-type cycloadducts by using cyclic (*s*-cis) 1,3-dienes proceeds by a two-step process, involving an initial hetero Diels–Alder cycloaddition of the diene and the ketene carbonyl group, followed by a [3,3]-sigmatropic (Claisen) rearrangement [25–26].

The most effective conditions were achieved by the employment of a slightly excessive amount of olefins **1**. The cycloadditions proceeded with complete regio- and stereoselectivity with respect to the olefin **1**, but less so with respect to the ketene **3** (Table 1), such that *endo-****4*** and *exo*-**4** isomers were formed in variable amounts, depending on the nature of the substrates. In general, the conversions of cyclopenta-1,3-diene **1a** and the spiroheptadiene (**1c**) gave high yields of product **4** at 0 °C. However, the yields were moderate to low with cyclic mono olefin **1f** or styrene (**1g**), even under reflux conditions. Mixtures of *endo*- and *exo*-isomers of compounds **4** were used in the subsequent ring-opening step without prior separation.

**Table 1:** Formal [2 + 2] cycloaddition of vinylketenes **2** with olefins.^a^

entry	alkene **1**	acid chloride **2**	time^b^ (h)	product **4**	*endo:exo^c^*	yield*^d^* (%)

1	**1a**	**2a**	3	**4a**	30:70	76
2	**1b**	**2a**	3	**4b**	50:50	80
3	**1c**	**2a**	4	**4c**	25:75	40
4	**1d**	**2a**	8	**4d**	50:50	34
5	**1e**	**2a**	8	**4e**	35:65	40
6*^e^*	**1f**	**2a**	10	**4f**	25:75	60
7*^e^*	**1g**	**2a**	12	**4g**	33:67*^f^*	16
8	**1a**	**2b**	3	**4h**	95:5	85
9	**1e**	**2b**	7	**4i**	>95:5	21

^a^Reagents and conditions (cf. Scheme 2): alkene **1** (1.5 equiv), Et_3_N (1.2 equiv), acid chloride **2** (1.0 equiv), 0 °C. ^b^Time required for the olefin to achieve maximal conversion. ^c^the *exo*/*endo* ratio was determined by ^1^H NMR spectroscopy. ^d^Isolated yields after purification by column chromatography on silica gel. ^e^Using chloroform as solvent under reflux. ^f^*cis*/*trans* ratio. Structures are shown in Scheme 2.

**Figure 1:**
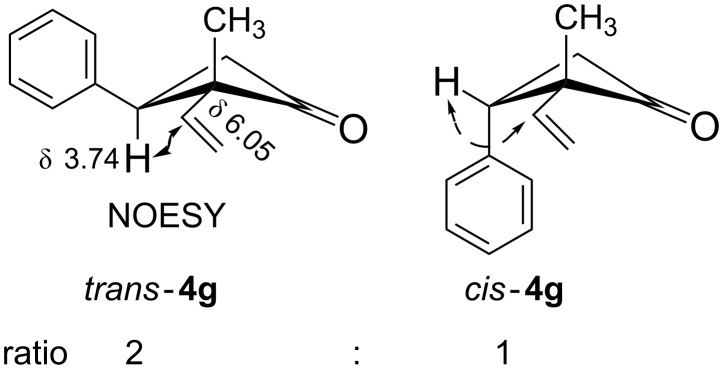
Determination of the structure of 3-phenyl-2-vinyl substituted cyclobutanone **4g**.

The configurations were determined from their ^1^H NMR spectra and mechanistic considerations [27]. For example, the C-7 methyl signal in the *endo*-bicyclo[3.2.0]heptenone systems was found in ^1^H NMR at a lower field (δ = 1.40 for *endo*-**4a**), while that for the *exo*-counterpart appeared at a higher field (δ = 1.10 for *exo*-**4a**). The configuration of compound **4g** was discernible from ^1^H NMR spectra and confirmed by 2D NMR spectroscopy. Thus, the NOESY spectrum of *trans*-**4g** indicated a correlation between the signal at 6.05 ppm from the α-vinyl proton of the vinyl group and the triplet at 3.74 ppm from the C-3 proton (Figure 1).

Taking the high degree of ring strain in substrates **4** into account, an alkoxide-promoted ring opening to vicinally functionalized products **5** or **6** was expected. To validate this proposal, the 7-methyl-7-vinyl substituted bicyclo[3.2.0]heptenone **4a** was employed as a model substrate with sodium methoxide or potassium *tert*-butoxide as the base. Various reaction conditions, including the amount of metal alkoxide, solvents and reaction temperature were examined. Representative results are listed in Table 2.

**Table 2:** Cleavage reaction of **4a** with metal alkoxide.^a^

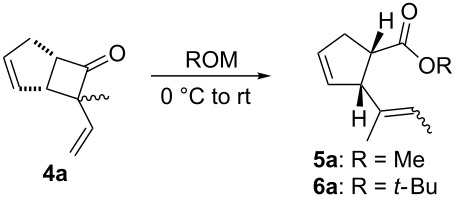						

entry	alkoxide (equiv)	solvent	time (h)	product	*E*:*Z*^b^	yield^c^ (%)

1	NaOMe (1.2)	MeOH	2	**5a**	60:40	40
2	NaOMe (0.010)	MeOH	16	**5a**	55:45	<10
3	*t-*BuOK (1.2)	THF	2	**6a**	9:1	46
4	*t-*BuOK (0.010)	THF	16	**6a**	75:25	<10
5^d^	*t-*BuOK (1.2)	THF	0.5	**6a**	—	decomp.
6	*t-*BuOK (1.2)	*t-*BuOH	24	**6a**	>99:1	30
7	*t-*BuOK (0.010)	*t-*BuOH	24	**6a**	95:5	<10
8	*t-*BuOK (1.2)^e^	THF	2	**6a**	90:10	47

^a^Addition of **4a** to the alkoxide was performed at 0 °C, then continued at rt. ^b^*E*/Z ratio was determined by GC–MS prior to chromatography. ^c^Isolated yield after column chromatography on silica gel. ^d^Under reflux. ^e^Using 0.05 equiv dibenzo-18-C-6 as a K-chelating agent.

It was found that both MeONa and *t*-BuOK can promote the ring opening to furnish the alkyl 2-substituted cyclopent-3-enecarboxylate **5a** or **6a**. Using 1.2 equiv of MeONa with methanol as solvent gave **5a** in moderate isolated yield, after 2 h at room temperature, in form of an *E*/*Z* isomeric mixture about the 2-vinyl double bond, in a ratio of 6:4 (Table 2, entry 1). Reducing the amount of the base significantly decreased the yield. Using 0.01 equiv of MeONa afforded no more than 10% yield, even though the reaction time was prolonged to 16 h (Table 2, entry 2). The more bulky *tert*-BuOK was also a suitable base to trigger the ring opening, giving acceptable yields of product **6a**. Not surprisingly, the use of *tert-*BuOK as base provided **6a** with a higher diastereoselectivity of 9:1 in favour of the *E*-isomer. It seems that 1.2 equiv of the base had to be applied, and THF as solvent was superior to *tert*-butanol in terms of yield and reaction rate (Table 2, entry 3 versus 4, entry 3 versus 6). A detrimental effect of enhancing the temperature was observed. Thus, an attempt to accelerate the reaction led to the decomposition of the cyclobutanone **4a** under reflux conditions, without the detection of any desired product **6a** (Table 2, entry 5). In addition, the use of a K-chelating agent, i.e., dibenzo-18-crown-6, did not show any improvement of the yield of compound **6a** (Table 2, entry 8).

**Scheme 3:**
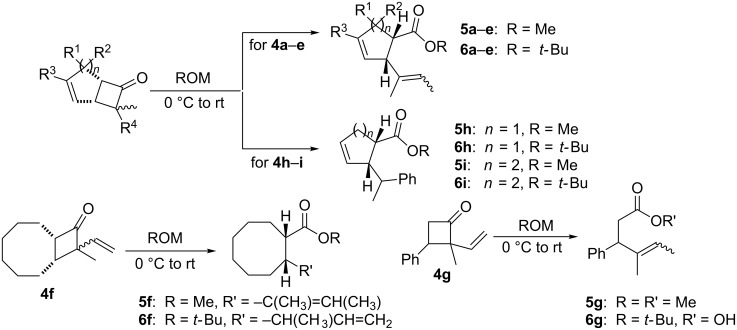
Ring opening of cyclobutanones **4** to afford products **5** or **6**.

In light of the above promising results, the substrate scope of this base-promoted ring-opening reaction was assessed by using a variety of cyclobutanones **4b**–**i** (Scheme 3, Table 3). Diverse substitution patterns at both the ketene and the olefin moiety were compatible with this ring-opened method. Thus, cyclobutanones carrying both vinyl (Table 3, entries 1–14) and phenyl (Table 3, entries 15–18) substituents underwent the cleavage reaction. The monocyclic butanone **4g** was also tolerated to give moderate yields (Table 3, entries 13 and 14). Another noticeable observation is that the use of *t-*BuOK furnished generally better yields than MeONa, except for compound **4d**, which was obtained in a slightly higher yield in the presence of MeONa as the base (Table 3, entries 7 and 8).

**Table 3:** Reaction of **4** with MeONa or *t-*BuOK.^a^

entry	ketone **4**	ROM	time (h)	product	product label	*E*:*Z^b^*	yield^c^ (%)

1 2	**4a** **4a**	MeONa *t-*BuOK	2 2	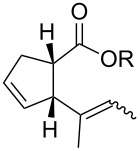	**5a** **6a**	60:40 9:1	40 46
3 4	**4b** **4b**	MeONa *t-*BuOK	4 6	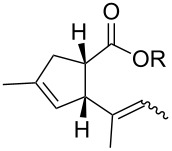	**5b** **6b**	50:50 95:5	41 50
5 6	**4c** **4c**	MeONa *t-*BuOK	4 16	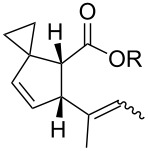	**5c** **6c**	67:33 80:20	18 43
7 8	**4d** **4d**	MeONa *t-*BuOK	24 24	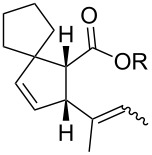	**5d** **6d**	60:40 >99:1	37 29
9	**4e**	MeONa	14	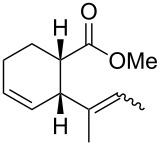	**5e**	55:45	41
10	**4e**	*t-*BuOK	3	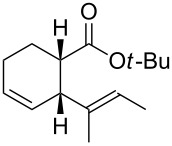	**6e**	>99:1	72
11	**4f**	MeONa	3	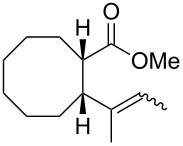	**5f**	60:40	51
12	**4f**	*t-*BuOK	5	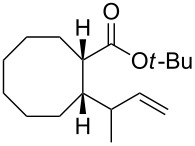	**6f**	-	60
13	**4g**	MeONa	2	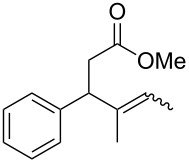	**5g**	60:40	45
14	**4g**	*t-*BuOK	5	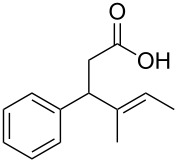	**6g**	>99:1	65
15 16	**4h** **4h**	MeONa *t-*BuOK	12 2	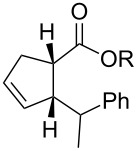	**5h** **6h**	- -	63 74
17 18	**4i** **4i**	MeONa *t-*BuOK	6 3	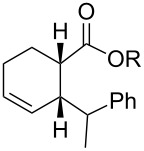	**5i** **6i**	- -	57 70

^a^1.2 equiv of base was employed. For MeONa methanol was the solvent, and for *t-*BuOK THF was the solvent. The progression was monitored by GC. ^b^The *E*/Z ratio was determined by GC-MS before chromatography. ^c^Isolated yield after silica-gel column chromatography.

**Scheme 4:**
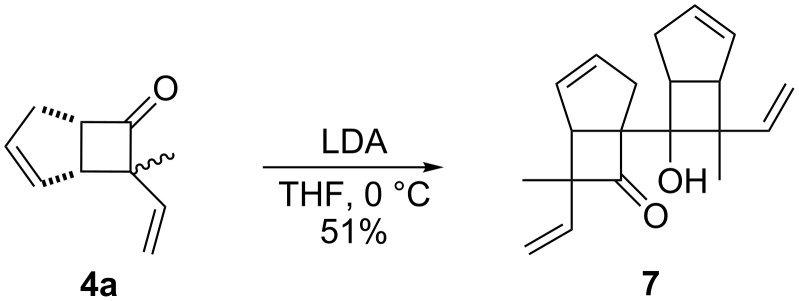
Reaction of **4a** with LDA.

Surprisingly, the reaction of **4f** with *t-*BuOK led to ester **6f** (Table 3, entry 12) carrying an allylic instead of a vinylic side chain. In the case of the 2-phenyl substituted cyclobutanone **4g** the reaction afforded a 65% yield of γ,δ-unsaturated acid product (*E*)-**6g** (Table 3, entry 14). Finally, an attempt using the sterically hindered base LDA only led to the formation of aldol adduct **7** (Scheme 4).

**Scheme 5:**
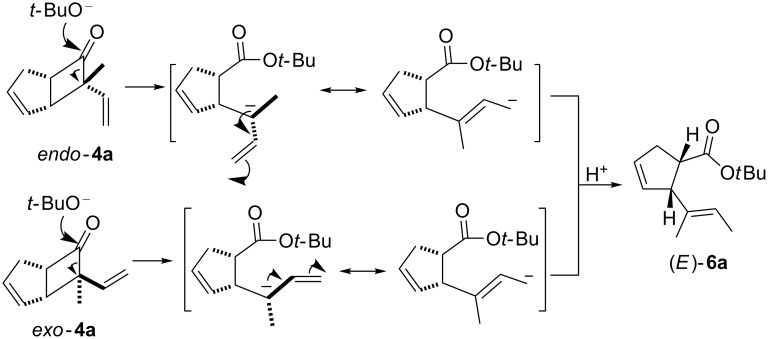
Plausible mechanism for ring opening of **4a**.

Although the mechanism is not fully understood, a tentative mechanistic rationale is depicted in Scheme 5. Due to the high ring strain of the cyclobutanones, the ring is very sensitive to the influence of nucleophiles. As exemplified by **4a**, the reaction begins with the nucleophilic *exo*-facial attack on the carbonyl group by an alkoxide ion to trigger the cleavage of the four-membered ring. The presence of a vinyl group facilitates the ring fission since the resultant carbanion can be stabilized by resonance. Protonation leads to the formation of the isolated products **5a** or **6a**. When the hindered base *t-*BuOK is employed, the reaction occurs with a high preference for *E*-product formation. This may be a result of thermodynamic control.

## Conclusion

In conclusion, our intention to synthesize potential olfactorily useful compounds led us to disclose the facial ring opening of a series of cyclobutanones to produce 1,2-disubstituted cyclopentene derivatives with high selectivity. The economic cycloaddition/ring-opening sequence is significant in that it allows a useful functional group to be easily introduced under mild conditions. Furthermore, the products have a general γ,δ-unsaturated carbonyl skeleton, and hence the protocol should be a good surrogate for the well-established Johnson orthoester Claisen rearrangement [28–29].

## Supporting Information

File 1 Detailed experimental procedures.

File 2 NMR spectral data for unknown compounds.
