# Supplementary material for: Alkoxide-induced ring opening of bicyclic 2-vinylcyclobutanones: A convenient synthesis of 2-vinyl-substituted 3-cycloalkene-1-carboxylic acid esters
Source: Beilstein J Org Chem. 2012 Apr 26;8:650–7. doi: 10.3762/bjoc.8.72 (PMC3388850; doi:10.3762/bjoc.8.72)
Supplement: File 1 — Detailed experimental procedures. [file Beilstein_J_Org_Chem-08-650-s001.pdf]

Supporting Information

for

Alkoxide-induced ring opening of bicyclic 2-vinylcyclobutanones: A convenient synthesis of 2-vinyl-substituted 3-cycloalkene-1-carboxylic acid esters

Xiufang Ji[1], Zhiming Li[1], Quanrui Wang*[1] and Andreas Goeke*[2]

Address: [1]Department of Chemistry, Fudan University, 220 Handan Road, 200433 Shanghai, P. R. China and [2]Shanghai Givaudan Ltd., Fragrances, 298 Li Shi Zhen Road, 201203 Shanghai, P. R. China

Email: Quanrui Wang* - qrwang@fudan.edu.cn; Andreas Goeke* - andreas.goeke@givaudan.com

*Corresponding author

Detailed experimental procedures

General Information……………………………………………………………S2
Synthesis of substrates **4**……………………………………………………S3
Reaction of **4** with NaOMe/MeOH (general procedure) ……………………S6
Analytical data of the unknown compounds **5**……………………………S6
Reaction of **4** with *t*-BuOK/THF (general procedure) ………………………S11
Analytical data of the unknown compounds **6**…………………………S12
Synthesis of adol adduct **7**…………………………………………………S15
References…………………………………………………………………S16

Experimental procedures and analytical data for new compounds

1. General: ^{1}H and ^{13}C NMR spectra were recorded with AW 300 and AV2 400 MHz Bruker spectrometer instruments in $CDCl_3$. Chemical shifts in $CDCl_3$ and C_6D_6 are reported in δ (ppm) relative to tetramethylsilane (TMS), chloroform and benzene as internal references unless otherwise stated. In the ^{13}C NMR spectra, the nature of the carbons (C, CH, CH_2, or CH_3) was determined by DEPT-90 and DEPT-135 experiments, and is given in parentheses. The following abbreviations are used: s = singlet, d = doublet, t = triplet, q = quartet, m = multiplet, dd = double doublet, bs = broad singlet, sept = septet. Solvents for extraction and chromatography were technical grade and used without further purification. Flash chromatography was performed by using Tsingdao Haiyang Chemical silica gel (200–300 mesh) and silica gel Merck grade (60 Å). IR spectra were recorded with a Bruker Tensor 27 and a Jasco FT/IR-4100. High-resolution MS were obtained with a Finnigan MAT 95 (San Jose, CA; USA) double-focusing magnetic-sector mass spectrometer (geometry BE); for compound **7** it was obtained with a micrOTOF II. GC–MS spectral data were obtained from an Agilent 6890 N and MSD 5975 by using a column HP-5 MS, 30 m, 0.25 mm, 0.25 µm. *E*/*Z* ratios were determined by GC-MS. All solvents were dried over standard drying agent and freshly distilled prior to use. All other reagents were used as received. Substrates **4a**,**c**,**e,f**,**h** were synthesized according to literature protocols [1-5]. Unless otherwise noted, for purifications by chromatography, a mixture of hexane:MTBE (50:1) was used as eluent.

2. General procedure for the preparation of cyclobutanones 4:

Freshly prepared acyl chloride **2** (0.20 mol in 200 mL of dry CH_2Cl_2 or $CHCl_3$) was added dropwise to a solution of triethylamine (24.29 g, 0.24 mol) and diene **1** (0.30 mol) in CH_2Cl_2 or $CHCl_3$ (300 mL) at 0 °C during 1.5 h. After being stirred for several hours at room temperature or under reflux, the mixture was filtered and concentrated in vacuo. The residue was diluted with hexane (300 mL), washed with water and brine, dried ($MgSO_4$), concentrated in vacuo. The residue was purified by silica gel chromatography to provide the pure product **4**.

7-Methyl-7-vinylbicyclo[3.2.0]hept-2-en-6-one (4a) [1]

Yield 22.50 g (76%); colorless oil.

3,7-Dimethyl-7-vinylbicyclo[3.2.0]hept-2-en-6-one (4b)

Yield 25.92 g (80%); colorless liquid; IR (KBr): 3086, 3041, 2924, 2848, 1775, 1631, 1442, 1368 cm^{-1}; ^1H NMR (300 MHz, $CDCl_3$) δ 5.86 (dd, *J* = 10.2, 17.1 Hz, 0.5 H), 5.70 (dd, *J* = 10.8, 17.4 Hz, 0.5H), 5.30–5.19 (m, 1H), 5.08–4.99 (m, 2H), 3.92–3.84 (m, 1H), 3.32–3.14 (m, 1H), 2.48–2.21 (m, 2H), 1.68 (s, 3H), 1.32 (s, 1.5H), 1.01 (s, 1.5H); ^{13}C NMR (75 MHz, $CDCl_3$) δ 215.8 (s), 215.2 (s), 144.8 (s), 144,1 (s), 139.4 (d), 135.6 (d), 124.4 (d), 123.5 (d), 115.2 (t), 113.5 (t), 71.0 (s), 69.4 (s), 59.3 (d), 58.6 (d), 51.2 (d), 48.7 (d), 37.8 (t), 21.4 (q), 16.7 (q), 16.6 (q), 15.9 (q); EIMS: *m*/*z* (% relative intensity): 162 $[M]^+$ (18), 147 (20), 119 (27), 91 (26), 82 (44), 80 (100), 79 (42); HRMS–EI (*m*/*z*): $[M]^+$ calcd for $C_{11}H_{14}O$, 162.1045; found, 162.1037.

6-Methyl-6-vinylspiro[bicyclo[3.2.0]hept[3]ene-2,1'-cyclopropan]-7-one (4c) [2]

Yield 13.92 g (40%); colorless oil.

6-Methyl-6-vinylspiro[bicyclo[3.2.0]hept[3]ene-2,1'-cyclopentan]-7-one (4d)

Yield 13.74 g (34%); colorless oil; IR (KBr): 3048, 2931, 1772, 1612, 1450, 919 cm^{-1}; ^{1}H NMR (300 MHz, $CDCl_3$) δ 5.94 (dd, *J* = 10.5, 17.4 Hz, 1H), 5.79–5.59 (m, 2H), 5.12–5.07 (m, 2H), 3.90–3.84 (m, 0.5H), 3.53–3.48 (m, 1H), 3.32–3.24 (m, 0.5H), 1.38–1.78 (m, 8H), 1.13 (s, 1.5H), 1.08 (s, 1.5H); ^{13}C NMR (75 MHz, $CDCl_3$) δ 216.3 (s), 143.4 (d), 139.7 (d), 126.8 (d), 113.5 (t), 70.9 (s), 58.9 (s), 57.5 (d), 48.0 (d), 37.3 (t), 33.9 (t), 26.5 (t), 26.1 (t), 16.0 (q); EIMS: *m*/*z* (% relative intensity): 202 $[M]^+$ (18), 174 (16), 120 (100), 91 (64), 82 (13); HRMS–EI (*m*/*z*): $[M]^+$ calcd for $C_{14}H_{18}O$, 202.1358; found, 202.1365.

8-Methyl-8-vinylbicyclo[4.2.0]oct-2-en-7-one (4e) [3]

Yield 12.97 g (40%); colorless liquid; IR (KBr): 3024, 2927, 2850, 1772, 1631, 1449, 1369 cm^{-1}; ^{1}H NMR (300 MHz, $CDCl_3$) δ 6.03–5.92 (m, 2H), 5.82–5.71 (m, 1H), 5.20–5.07 (m, 2H), 3.79–3.72 (m, 1H), 2.92–2.71 (m, 1H), 2.06–1.84 (m, 4H), 1.45 (s, 1H), 1.09 (s, 2H).

10-Methyl-10-vinylbicyclo[6.2.0]decan-9-one (4f) [4]

Yield 23.01 g (60%); colorless liquid; IR (KBr): 3028, 2923, 1728, 1630, 1434, 1160 cm^{-1}; ^{1}H NMR (300 MHz, $CDCl_3$) δ 5.93 (dd, *J* = 10.5, 17.4 Hz, 0.7H), 5.75 (dd, *J* = 10.5, 17.4 Hz, 0.3H), 5.16–5.02 (m, 2H), 3.35–3.22 (m, 1H),

2.42–2.21 (m, 0.7H), 2.18–2.13 (m, 0.3H), 1.91–1.44 (m, 12H), 1.31 (s, 1H), 1.09 (s, 2H).

2-Methyl-3-phenyl-2-vinylcyclobutanone (4g)

Yield 5.95 g (16%); *cis*:*trans* = 1:2; colorless liquid; IR (KBr) 3086, 3061, 3030, 2969, 2926, 1779, 1634, 1497, 923, 763, 701 cm^{-1}; Major isomer (*trans*-**4g**) ^{1}H NMR (300 MHz, $CDCl_3$) δ 7.52–7.05 (m, 5H), 6.05 (dd, *J* = 10.5, 17.2 Hz, 1H), 5.31 (d, *J* = 17.2 Hz, 1H), 5.16 (d, *J* = 10.5, 1H), 3.74 (t, *J* = 9.0 Hz, 1H), 3.54–3.21 (m, 2H), 0.91 (s, 3H); ^{13}C NMR (75 MHz, $CDCl_3$) δ 210.0 (s), 138.9 (d), 138.3 (s), 128.5 (d), 127.8 (2d), 126.8 (2d), 114.6 (t), 69.9 (s), 46.0 (t), 39.6 (d), 17.0 (q); Minor isomer (*cis*-**4g**) ^{1}H NMR (300 MHz, $CDCl_3$) δ 7.52–7.05 (m, 5H), 5.33 (dd, *J* = 10.7, 17.4 Hz, 1H), 5.05 (d, *J* = 17.4 Hz, 1H), 4.95 (d, *J* = 10.7 Hz, 1H), 3.54–3.21 (m, 3H), 1.45 (s, 3H); ^{13}C NMR (75 MHz, $CDCl_3$) δ 209.4 (s), 138.5 (s), 135.7 (d), 128.4 (2d), 127.9 (2d), 126.8 (d), 115.3 (t), 70.0 (s), 46.0 (t), 39.6 (d), 17.0 (q); EIMS *m*/*z* (% relative intensity): 186 $[M]^+$ (6), 144 (54), 129 (100), 82 (64), 77 (10); HRMS–EI (*m*/*z*): $[M]^+$ calcd for $C_{13}H_{14}O$, 186.1045; found, 186.1046.

7-Methyl-7-phenylbicyclo[3.2.0]hept-2-en-6-one (4h) [5]

Yield 33.70 g (85%); colorless liquid; IR (KBr) 3027, 2974, 1728, 1603, 1392, 1151, 911, 765, 701 cm^{-1}; ^{1}H NMR (300 MHz, $CDCl_3$) δ 7.34–7.18 (m, 5H), 5.69–5.65 (m, 1H), 5.51-5.47 (m, 1H), 4.02 (dd, *J* = 8.1, 8.1Hz, 1H), 3.59–3.56 (m, 1H), 2.71–2.64 (m, 1H), 2.52–2.42 (m, 1H), 1.66 (s, 3H).

8-Methyl-8-phenylbicyclo[4.2.0]oct-2-en-7-one (4i)

Yield 8.92 g (21%); colorless liquid; IR (KBr): 3028, 2973, 2850, 1731, 1631, 1494, 1029, 701 cm^{-1}; ^{1}H NMR (300 MHz, $CDCl_3$) δ 7.38–7.18 (m, 5H), 5.68–5.54 (m, 2H), 3.89–3.74 (m, 1H), 2.99–2.96 (m, 1H), 2.10–1.96 (m, 2H), 1.71 (s, 3H), 1.61–1.51 (m, 2H); ^{13}C NMR (75 MHz, $CDCl_3$) δ 215.5 (s), 140.4 (s), 128.8 (d), 128.1 (2d), 127.1 (d), 126.4 (2d), 126.4 (d), 68.6 (s), 52.7 (d), 37.3 (d), 27.3 (q), 21.5 (t), 19.14 (t); EIMS *m*/*z* (% relative intensity): 212 $[M]^+$ (20), 184 (24), 132 (100), 105 (38), 80 (10), 77 (12).

3. Reaction of 4 with NaOMe/MeOH (general procedure):

To the solution of freshly prepared NaOMe (0.65 g, 0.012 mol) in MeOH (20 mL) was added dropwise cyclobutanone **4** (0.01 mol) in MeOH (20 mL) with stirring at 0 °C under argon, and the mixture was then stirred for several hours at 0 °C or under reflux. The volatiles were removed under reduce pressure. The residue was partitioned between MTBE (20 mL) and water (20 mL). The aqueous phase was extracted with MTBE (2 × 30 mL), and the organic phases were combined, washed with brine, dried ($MgSO_4$), and concentrated in vacuo. The residue was purified by silica-gel chromatography to provide the ring-opened ester product **5**.

Methyl 2-(but-2-en-2-yl)cyclopent-3-enecarboxylate (5a)

Yield 0.72 g (40%); *E*:*Z* = 60:40; colorless liquid; IR (KBr): 2926, 1739, 1662, 1437, 1165 cm^{-1}; ^{1}H NMR (300 MHz, $CDCl_3$) δ 5.75–5.69 (m, 1H), 5.53–5.42 (m, 1H), 5.36–5.30 (m, 1H), 4.19–4.14(m, 0.5H), 3.69 (s, 3H), 3.62–3.59 (m,

0.5H), 2.93–2.80 (m, 1 H), 2.72–2.61 (m, 2H), 1.64–1.55 (m, 6H); ^{13}C NMR (75 MHz, $CDCl_3$) δ 176.4 (s), 176.3 (s), 136.6 (s), 135.7 (s), 132.6 (d), 132.5 (d), 129.3 (d), 129.1 (d), 121.2 (d), 119.7 (d), 58.7 (d), 51.7 (q), 51.7 (q), 50.3 (d), 47.3 (d), 46.5 (d), 36.6 (t), 36.6 (t), 19.4 (Me from *E*-**5a**, q), 13.3 (q), 13.0 (q); EIMS *m*/*z* (% relative intensity): 180 $[M]^+$ (40), 125 (12), 121 (78), 93 (100), 77 (38); EI–HRMS (*m*/*z*). $[M]^+$ calcd for $C_{11}H_{16}O_2$, 180.1150; found, 180.1151.

Methyl 2-(but-2-en-2-yl)-4-methylcyclopent-3-enecarboxylate (5b)

Yield 0.79 g (41%); *E*:*Z*= 50:50; colorless liquid; IR (KBr): 2917, 2858, 1737, 1667, 1436 cm^{-1}; ^{1}H NMR (300 MHz, $CDCl_3$) δ 5.26 (d, *J* = 6.0 Hz, 1H), 5.07–4.99 (m, 1H), 4.11–4.07 (m, 0.5H), 3.65 (s, 3H), 3.56–3.52 (m, 0.5H), 2.91–2.78 (m, 1H), 2.64–2.46 (m, 2H), 1.70 (s, 3H), 1.58–1.50 (m, 6H); ^{13}C NMR (75 MHz, $CDCl_3$) δ 176.6 (s), 176.4 (s), 139.0 (s), 138.9 (s), 137.2 (s), 136.4 (s), 126.3 (d), 126.1 (d), 120.7 (d), 119.2 (d), 58.9 (d), 51.7 (q), 51.6 (q), 50.4 (d), 48.0 (d), 47.2 (d), 40.5 (t), 40.5 (t), 19.4 (Me from *E*-**5b**, q), 16.2 (q), 13.3 (q), 12.9 (q); EIMS *m*/*z* (% relative intensity): 194 $[M]^+$ (96), 179 (10), 139 (12), 135 (100), 119 (82), 107 (66), 91 (60), 79 (61); EI–HRMS (*m*/*z*): $[M]^+$ calcd for $C_{12}H_{18}O_2$, 194.1307; found, 194.1312.

Methyl 5-(but-2-en-2-yl)spiro[4.2]hept-6-ene-4-carboxylate (5c)

Yield 0.37 g (18%); *E*:*Z* = 67:33; colorless liquid IR (KBr): 2951, 1742, 1654, 1436, 1161 cm^{-1}; ^{1}H NMR (300 MHz, $CDCl_3$) δ 5.51 (d, *J* = 6.7 Hz, 0.2H), 5.44 (d, *J* = 6.7 Hz, 0.8H), 5.36–5.26 (m, 1H), 5.23-5.19 (m, 1H), 4.49–4.36 (m, 0.8H), 3.88–3.86(m, 0.2H), 3.67 (s, 3H), 2.79 (d, *J* = 6.0 Hz, 0.8H), 2.75 (d, *J* =

6.0 Hz, 0.2H), 1.64–1.57 (m, 6H), 0.85–0.68 (m, 4H); ^{13}C NMR (75 MHz, $CDCl_3$) δ 173.6 (s), 135.2 (d), 135.0 (s), 129.5 (d), 119.9 (d), 51.9 (d), 50.5 (q), 48.8 (d), 31.1 (s), 18.7 (Me from *E*-**5c**, q), 12.4 (t), 12.1 (q), 9.6 (t); EIMS *m*/*z* (% relative intensity): 206 $[M]^+$ (42), 191 (20), 147 (58), 131 (68), 119 (64), 105 (60), 91 (100); HRMS–EI (*m*/*z*): $[M]^+$ calcd for $C_{13}H_{18}O_2$, 206.1307; found, 206.1322.

Methyl 2-(but-2-en-2-yl)spiro[4.4]non-3-enecarboxylate (5d)

Yield 0.86 g (37%); *E*:*Z* = 60:40; colorless liquid; IR (KBr): 2953, 2864, 1737, 1670, 1379, 1160 cm^{-1}; ^{1}H NMR (300 MHz, $CDCl_3$) δ 5.67–5.52 (m, 1 H), 5.44 (d, *J* = 6.7 Hz, 0.4H), 5.39–5.30 (m, 1.6H), 4.36–4.32 (m, 1H), 3.67 (s, 3H), 2.83 (d, *J* = 8.6 Hz, 0.6H) 2.75 (d, *J* = 8.6 Hz, 0.4H), 1.82–1.63 (m, 8H), 1.58–1.53 (m, 6H); ^{13}C NMR (75 MHz, $CDCl_3$) δ 174.6 (s), 174.4 (s), 138.6 (d), 138.3 (d), 136.4 (s), 135.2 (s), 130.0 (d), 129.8 (d), 121.6 (d), 119.7 (d), 59.4 (s), 58.1 (d), 56.8 (d), 56.5 (d), 51.3 (q), 48.3 (d), 39.8 (t), 39.7 (t), 34.5 (t), 25.0 (2t), 24.3 (t), 24.2 (t), 19.6 (Me from *E*-**5d**, q), 13.6 (q), 13.1 (q); EIMS *m*/*z* (% relative intensity): 234 $[M]^+$ (10), 175 (22), 147 (100), 119 (76), 91 (56); HRMS–EI (*m*/*z*): $[M]^+$ calcd for $C_{15}H_{22}O_2$, 234.1620; found, 234.1620.

Methyl 2-(but-2-en-2-yl)cyclohex-3-enecarboxylate (5e)

Yield 0.80 g (41%); *E*:*Z* = 55:45; colorless liquid; IR (KBr): 3023, 2948, 1738, 1435, 1160 cm^{-1}; ^{1}H NMR (300 MHz, $CDCl_3$) δ 5.79–5.72 (m, 1H), 5.47–5.24 (m, 2H), 3.64 (s, 3H), 3.68–3.61 (m, 0.5H), 3.09–3.04 (m, 0.5H), 2.60–2.48 (m, 1H), 2.18–2.02 (m, 2H), 2.02–1.70 (m, 2H), 1.68–1.49 (m, 6H); ^{13}C NMR (75

MHz, $CDCl_3$); δ 176.0 (s), 176.0 (s), 136.4 (s), 136.0 (s), 129.7 (d), 129.6 (d), 127.0 (d), 126.9 (d), 121.7 (d), 121.2 (d), 51.4 (q), 51.3 (q), 48.0 (d), 44.0 (d), 43.3 (d), 39.6 (d), 25.9 (t), 25.0 (t), 24.4 (t), 24.2 (t), 19.2 (Me from *E*-**5e**, q), 13.4 (q), 12.9 (q), 12.8 (q); EIMS *m*/*z* (% relative intensity): 194 $[M]^+$ (79), 180 (8), 176 (26), 164 (60), 135 (56), 105 (100), 93 (99), 57 (98); HRMS–EI (*m*/*z*): $[M]^+$ calcd for $C_{12}H_{18}O_2$, 194.1307; found, 194.1309.

Methyl 2-(but-2-en-2-yl)cyclooctanecarboxylate (5f)

Yield 1.14 g (51%); *E*:*Z* = 60:40; colorless liquid; IR (KBr): 2923, 1728, 1434, 1160 cm^{-1}; ^1H NMR (300 MHz, $CDCl_3$) δ 5.27–5.01 (m, 1H), 3.57 (s, 3H), 3.15–2.96 (m, 0.6H), 2.53–2.39 (m, 0.4H), 2.71–2.55 (m, 1H), 1.99–1.32 (m, 18H); ^{13}C NMR (75 MHz, $CDCl_3$) δ 176.5 (s), 176.1 (s), 139.4 (s), 139.2 (s), 119.1 (d), 118.3 (d), 50.9 (q), 49.1 (d), 47.9 (d), 47.6 (d), 39.8 (d), 30.4 (t), 29.8 (t), 27.8 (t), 27.7 (t), 27.6 (t), 27.1 (t), 26.1 (t), 25.9 (t), 25.7 (t), 25.6 (t), 25.4 (t), 18.8 (Me from *E*-**5f**, q), 13.2 (q), 13.0 (q), 12.9 (q); EIMS *m*/*z* (% relative intensity): 224 $[M]^+$ (80), 167 (58), 109 (90), 95 (100), 67 (60); EI–HRMS (*m*/*z*): $[M]^+$ calcd for $C_{14}H_{24}O_2$, 224.1776; found, 224.1777.

Methyl 4-methyl-3-phenylhex-4-enoate (5g)

Yield 0.92 g (45%); *E*:*Z* = 60:40; colorless liquid; IR (KBr) 3028, 2951, 1741, 1437, 1260, 1196, 701 cm^{-1}; ^1H NMR (300 MHz, $CDCl_3$) δ 7.31–7.17 (m, 5H), 5.48–5.41 (m, 1H), 4.51 (t, *J* = 10.4 Hz, 0.5H), 3.79 (t, *J* = 8.1 Hz, 0.5H), 3.59 (s, 3H), 2.93–2.67 (m, 2H), 1.61 (d, *J* = 6.6 Hz, 3H), 1.47 (s, 3H); ^{13}C NMR (75 MHz, $CDCl_3$) δ 171.8 (s), 171.7 (s), 141.5 (s), 141.1 (s), 135.8 (s), 135.3 (s),

127.3 (2d), 127.3 (2d), 126.6 (2d), 126.1 (2d), 125.4 (d), 125.2 (d), 120.5 (d), 118.1 (d), 50.6 (q), 50.5 (q), 48.8 (d), 39.9 (d), 37.5 (t), 35.3 (t), 17.8 (Me from *E*-**5g**, q), 13.5 (q), 12.4 (q), 12.3 (q); EIMS *m*/*z* (% relative intensity): 218 $[M]^+$ (64), 158 (24), 145 (100), 129 (48), 117 (24), 77 (12).

Methyl 2-(1-phenylethyl)cyclopent-3-enecarboxylate (5h)

Yield 1.45 g (63%); colorless liquid; IR (KBr): 3060, 2963, 1735, 1612, 1494, 1452, 1162, 702 cm^{-1}; ^{1}H NMR (300 MHz, $CDCl_3$) δ 7.37–7.10 (m, 5H), 5.81–5.72 (m, 0.7H), 5.72–5.64 (m, 0.7H), 5.61–5.52 (m, 0.3H), 5.44–5.36 (m, 0.3H), 3.43 (s, 1H), 3.66 (s, 2H), 3.38–3.24 (m, 1H), 2.91–2.41 (m, 4H), 1.29 (d, *J* = 6.9, 7.0 Hz, 3H); Major isomer ^{13}C NMR (75 MHz, $CDCl_3$) δ 176.6 (s), 145.3 (s), 131.8 (d), 129.3 (d), 128.2 (2d), 127.8 (2d), 127.7 (d), 56.9 (d), 51.5 (q), 46.4 (d), 44.7 (d), 36.8 (t), 19.2 (q); Minor isomer ^{13}C NMR (75 MHz, $CDCl_3$) δ 176.8 (s), 145.2 (s), 132.2 (d), 128.6 (d), 128.2 (2d), 127.8 (2d), 126.2 (d), 56.8 (d), 51.8 (q), 46.8 (d), 44.8 (d), 37.1 (t), 19.8 (q); EIMS *m*/*z* (% relative intensity: 230 $[M]^+$ (10), 199 (4), 125 (6), 105 (100), 77 (6); EI–HRMS (*m*/*z*): $[M]^+$ calcd for $C_{15}H_{18}O_2$, 230.1307; found, 230.1302.

Methyl 2-(1-phenylethyl)cyclohex-3-enecarboxylate (5i)

Yield 1.39 g (57%); colorless liquid; IR (KBr) 3068, 2970, 1737, 1491, 1151, 920, 718, 701 cm^{-1}; ^{1}H NMR (300 MHz, $CDCl_3$) δ 7.42–7.07 (m, 5H), 5.90–5.49 (m, 2H), 3.65 (s, 1.5H), 3.62 (s, 1.5H), 2.92-2.70 (m, 2H), 2.51–2.32 (m, 1H), 2.08–2.00 (m, 1H), 1.94–1.70 (m, 3H), 1.31 (d, *J* = 6.7 Hz, 3H); ^{13}C NMR (75 MHz, $CDCl_3$) δ 175.6 (s), 175.2 (s), 144.5 (s), 143.2 (s), 127.2 (2d),

127.2 (2d), 127.0 (2d), 126.8 (d), 126.7 (2d), 126.5 (d), 126.1 (d), 125.8 (d), 125.1 (d), 125.1 (d), 50.6 (q), 50.6 (q), 42.4 (d), 42.2 (d), 42.2 (d), 42.1 (d), 42.1 (d), 41.0 (d), 24.8 (t), 23.6 (t), 23.0 (t), 22.9 (t), 17.7 (q), 14.8 (q); MS (EI): *m*/*z* (%): 244 $[M]^+$ (6), 138 (6), 105 (100), 79 (18), 77 (8); EI–HRMS (*m*/*z*): $[M]^+$ calcd for $C_{16}H_{20}O_2$, 244.1463; found, 244.1469.

4. Reaction of 4 with *t*-BuOK/THF (general procedure):

To a solution of *t*-BuOK (1.34 g, 0.012 mol) in THF (20 mL) was added dropwise 0.01 mol of cyclobutanone **4** in THF (20 mL) with stirring at 0 °C under argon. The mixture was stirred for several hours and then quenched with water (20 mL). The mixture was extracted with MTBE (3 × 30 mL), and the organic phases were combined, washed with brine, dried with $MgSO_4$, and concentrated in vacuo. The residue was purified by silica-gel chromatography to provide the ring-opened ester product **6**.

***tert*-Butyl 2-(but-2-en-2-yl)cyclopent-3-enecarboxylate (6a)**

Yield 1.02 g (46%); *E*:*Z* = 9:1; colorless liquid; IR (KBr): 3057, 2929, 1728, 1453, 1367, 1151 cm^{-1}; ^1H NMR (300 MHz, $CDCl_3$) δ 5.73–5.67 (m, 1H), 5.51–5.39 (m, 1H), 5.33–5.27 (m, 1H), 4.14–4.10 (m, 0.2H), 3.54–3.52 (m, 0.8H), 2.74–2.66 (m, 1H), 2.63–2.59 (m, 2H), 1.67–1.55 (m, 6H), 1.45 (s, 9H); ^{13}C NMR (75 MHz, $CDCl_3$) δ 174.3 (s), 136.0 (s), 131.5 (d), 128.3 (d), 118.4 (d), 78.9 (s), 57.9 (d), 47.6 (d), 35.3 (t), 28.1 (3q), 12.5 (q), 12.3 (q); EIMS *m*/*z*

(% relative intensity): 222 $[M]^+$ (4), 166 (100), 121 (16), 57 (89); EI–HRMS (*m*/*z*): $[M]^+$ calcd for $C_{14}H_{22}O_2$, 222.1620; found, 222.1627.

***tert*-Butyl 2-(but-2-en-2-yl)-4-methylcyclopent-3-enecarboxylate (6b)**

Yield 1.17 g (50%); *E*:*Z* = 95:5; colorless liquid; IR (KBr): 2977, 2931, 1728, 1663, 1367 cm^{-1}; ^1H NMR (300 MHz, $CDCl_3$) δ 5.30 (d, *J* = 6.9 Hz, 1H), 5.09 (s, 1H), 3.54–3.43 (m, 1H), 2.74 (dd, *J* = 6.9, 15.9 Hz, 1H), 2.51 (m, 2H), 1.73 (s, 3H), 1.58 (d, *J* = 6.9 Hz, 3H), 1.55 (s, 3H), 1.46 (s, 9H); ^{13}C NMR (75 MHz, $CDCl_3$) δ 175.4 (s), 139.0 (s), 137.6 (s), 126.2 (d), 118.9 (d), 79.8 (s), 59.1 (d), 49.2 (d), 40.2 (t), 28.1 (3q), 16.3 (q), 13.4 (q), 13.3 (q); EIMS *m*/*z* (% relative intensity): 236 $[M]^+$ (6), 163 (29), 180 (100), 135 (82), 119 (24), 107 (45), 91 (21), 57 (40); EI–HRMS (*m*/*z*): $[M]^+$ calcd for $C_{15}H_{24}O_2$, 236.1776; found, 236.1780.

***tert*-Butyl 5-(but-2-en-2-yl) spiro[4.2]hept-6-ene-4-carboxylate (6c)**

Yield 1.07 g (43%); *E*:*Z*=80:20; yellow liquid; IR (KBr) 2977, 2930, 1730, 1667, 1456, 1367, 1147 cm^{-1}; ^1H NMR (300 MHz, $CDCl_3$) δ 5.51 (d, *J* = 6.7 Hz, 0.7H), 5.43 (d, *J* = 6.7 Hz, 0.3H), 5.38-5.26 (m, 1H), 5.22–5.15 (m, 1H), 4.44–4.32 (m, 0.3H), 3.89–3.74 (m, 0.7H), 2.61 (d, *J* = 5.8 Hz, 1H), 1.64–1.55 (m, 6H), 1.44 (s, 9H), 0.93–0.57 (m, 4H); ^{13}C NMR (75 MHz, $CDCl_3$) δ 173.5 (s), 137.2 (s), 136.2 (d), 130.5 (d), 119.1 (d), 80.3 (s), 58.1 (d), 54.6 (d), 31.8 (s), 28.2 (3q), 19.9 (Me from *E*-**6c**, q), 13.7 (q), 13.0 (t), 10.1 (t); EIMS *m*/*z* (% relative intensity): 248 $[M]^+$ (4), 192 (41), 147 (38), 119 (20), 91 (26), 57 (100); EI–HRMS (*m*/*z*): $[M]^+$ calcd for $C_{16}H_{24}O_2$, 248.1776; found, 248.1780.

tert-Butyl 2-(but-2-en-2-yl) spiro[4.4]non-3-enecarboxylate (6d)

Yield 0.80 g (29%); *E*:*Z* > 99:1; colorless liquid; IR (KBr): 3057, 2929, 1728, 1665, 1453, 1367, 1151 cm^{-1}; ^{1}H NMR (300 MHz, $CDCl_3$) δ 5.58–5.54 (m, 1H), 5.42 (d, *J* = 6.7 Hz, 1H), 5.34–5.27 (m, 1H), 3.68 (d, *J* = 8.3 Hz, 1H), 2.59 (d, *J* = 8.3 Hz, 1H), 1.81–1.62 (m, 8H), 1.58–1.51 (m, 6H), 1.45 (s, 9H); ^{13}C NMR (75 MHz, $CDCl_3$) δ 172.4 (s), 137.6 (d), 135.9 (s), 128.6 (d), 118.1 (d), 79.0 (s), 58.2 (d), 58.2 (s), 55.4 (d), 36.9 (t), 33.1 (t), 27.2 (3q), 24.2 (t), 23.4 (t), 12.9 (q), 12.3 (q); EIMS *m*/*z* (% relative intensity): 276 $[M]^+$ (3), 220 (100), 203 (16), 191 (40), 175 (90), 163 (28), 119 (14), 57 (12); EI–HRMS (*m*/*z*): $[M]^+$ calcd for $C_{18}H_{28}O_2$, 276.2089; found, 276.2083.

(*E*)-*tert*-Butyl 2-(but-2-en-2-yl)cyclohex-3-enecarboxylate (6e)

Yield 1.71 g (72%); *E*:*Z* > 99:1; colorless liquid; IR (KBr): 2977, 2932, 1729, 1665, 1454, 1367, 1149 cm^{-1}; ^{1}H NMR (300 MHz, $CDCl_3$) δ 5.74–5.70 (m, 1H), 5.43 (dd, *J* = 2.1, 10.2 Hz, 1H), 5.34–5.28 (m, 1H), 3.02–2.98 (m, 1H), 2.42–2.34 (m, 1H), 2.07–2.05 (m, 2H), 1.91–1.84 (m, 1H), 1.79–1.71 (m, 1H), 1.58–1.54 (m, 6H), 1.40 (s, 9H); ^{13}C NMR (75 MHz, $CDCl_3$) δ 174.0 (s), 135.6 (s), 128.9 (d), 125.9 (d), 120.0 (d), 78.7 (s), 47.7 (d), 43.7 (d), 27.1 (3q), 24.0 (t), 23.3 (t), 12.3 (q), 11.6 (q); EIMS *m*/*z* (% relative intensity): 236 $[M]^+$ (8), 180 (100), 135 (47), 107 (32), 93 (36), 79 (44), 57 (99).

***tert*-Butyl 2-(but-3-en-2-yl)cyclooctanecarboxylate (6f)**

Yield 1.59 g (60%); colorless liquid; IR (KBr): 3035, 3004, 2937, 1721, 1634, 1435 cm^{-1}; ^{1}H NMR (300 MHz, $CDCl_3$) δ 5.86 (dd, *J* = 10.8, 17.4 Hz, 1H), 5.23

(dd, J = 17.4 Hz, 1H), 5.11 (dd, J = 10.8 Hz, 1H), 2.47–2.33 (m, 1H), 2.33–2.14 (m, 1H), 2.04–1.88 (m, 1H), 1.88–1.23 (m, 15H), 1.24 (s, 9H); ^{13}C NMR (75 MHz, $CDCl_3$) δ 178.7 (s), 140.2 (d), 114.6 (t), 85.6 (s), 50.9 (d), 46.1 (d), 44.6 (d), 29.7 (t), 27.9 (t), 27.3 (t), 27.1 (t), 26.9 (t), 26.0 (t), 21.61 (q), 19.4 (3q); EIMS m/z (% relative intensity): 266 $[M]^+$ (10), 167 (26), 109 (100), 57 (90).

(*E*)-4-methyl-3-phenylhex-4-enoic acid (6g)

Yield 1.33 g (65%); *E*:*Z* > 99:1; white solid; IR (KBr) 3000, 1696, 1494, 702 cm^{-1}; ^{1}H NMR (300 MHz, $CDCl_3$) δ 11.3–9.9 (brs, 1H), 7.47–7.15 (m, 5H), 5.67–5.32 (m, 1H), 3.77 (t, J = 7.8 Hz, 1H), 2.89–2.63 (m, 2H), 1.61 (d, J = 6.6 Hz, 3H), 1.47 (s, 3H); ^{13}C NMR (75 MHz, $CDCl_3$) δ 178.6 (s), 142.4 (s), 136.6 (s), 128.4 (2d), 127.6 (2d), 126.5 (d), 119.4 (d), 49.5 (d), 38.5 (t), 14.6 (q), 13.4 (q); EIMS m/z (% relative intensity): 204 $[M]^+$ (50), 145 (100), 129 (72), 117 (37), 77 (15).

***tert*-Butyl 2-(1-phenylethyl)cyclopent-3-enecarboxylate (6h)**

Yield 2.01 g (74%); colorless liquid; IR (KBr) 3028, 2973, 1727, 1602, 1391, 1151, 911, 765, 701 cm^{-1}; ^{1}H NMR (300 MHz, $CDCl_3$) δ 7.18–7.06 (m, 5H), 5.45–5.42 (m, 1H), 5.27–5.24 (m, 1H), 3.18–3.13 (m, 1H), 2.65–2.54 (m, 2H), 2.45–2.43 (m, 2H), 1.33 (s, 9H), 1.19 (d, J = 7.8 Hz, 3H); ^{13}C NMR (75 MHz, $CDCl_3$) δ 174.5 (s), 144.5 (s), 131.3 (d), 127.5 (d), 127.2 (2d), 126.6 (2d), 125.0 (d), 78.8 (s), 55.9 (d), 46.5 (d), 43.9 (d), 36.1 (t), 27.0 (3q), 18.7 (q); EIMS m/z (% relative intensity): 272 $[M]^+$ (3), 216 (32), 199 (10), 105 (100), 77

(6), 57 (52); EI–HRMS (*m*/*z*): $[M]^+$ calcd for $C_{18}H_{24}O_2$, 272.1776; found 272.1773.

***tert*-Butyl 2-(1-phenylethyl)cyclohex-3-enecarboxylate (6i)**

Yield 2.00 g (70%); colorless liquid; IR (KBr): 3024, 2937, 1721, 1678, 1369, 1136, 911, 714 cm^{-1}; ^1H NMR (300 MHz, $CDCl_3$) δ 7.52–7.02 (m, 5H), 5.85–5.38 (m, 2H), 3.08–2.62 (m, 2H), 2.47–2.15 (m, 1H), 2.09–1.98 (m, 1H), 1.98–1.65 (m, 3H), 1.47 (s, 9H), 1.36 (d, *J* = 6.9 Hz, 1.5H), 1.23 (d, *J* = 7.1 Hz, 1.5H); ^{13}C NMR (75 MHz, $CDCl_3$) δ 175.7 (s), 175.2 (s), 145.7 (s), 144.5 (s), 128.3 (2d), 128.2 (2d), 128.0 (2d), 127.6 (2d), 127.5 (d), 127.5 (d), 126.9 (d), 126.7 (d), 126.1 (d), 126.0 (d), 80.0 (s), 80.0 (s), 44.7 (d), 44.1 (d), 43.8 (d), 43.2 (d), 43.1 (d), 41.2 (d), 28.1 (3q), 28.1 (3q), 25.9 (t), 24.9 (t), 24.1 (t), 19.8 (q), 14.8 (q); EIMS *m*/*z* (% relative intensity): 286 $[M]^+$ (2), 230 (20), 125 (18), 105 (100), 77 (8), 57 (22); EI–HRMS (*m*/*z*): $[M]^+$ calcd for $C_{19}H_{26}O_2$, 286.1933; found, 286.1917.

Adol adduct (7)

To a solution of LDA (1.28 g, 0.012 mol) in THF (20 mL) was added dropwise cyclobutanone **4a** (1.48 g, 0.010 mol) in THF (20 mL) with stirring at 0 °C under argon. The mixture was stirred for another six hours then quenched with water (20 mL). The mixture was extracted with MTBE (3 × 30 mL), and the organic phases were combined, washed with brine, dried with $MgSO_4$, and concentrated in vacuo. The residue was purified by silica-gel chromatography to provide the adol product **7**.

Yield 0.75 g (51%); white solid; IR (KBr): 3525, 3051, 2962, 2925, 1771, 1632, 1450, 1412 cm^{-1}; ^{1}H NMR (300 MHz, $CDCl_3$) δ 6.13 (dd, *J* = 17.4, 10.4 Hz, 1H), 6.01–5.88 (m, 3H), 5.77–5.72 (m, 2H), 5.16–4.97 (m, 4H), 3.72–3.51 (m, 1H), 3.28–3.09 (m, 2H), 2.69–2.54 (m, 2H), 2.47–2.18 (m, 2H), 1.57 (brs, 1H), 1.07 (s, 3H), 1.02 (s, 3H); ^{13}C NMR (75 MHz, $CDCl_3$) δ 220.0 (s), 143.3 (d), 137.7 (d), 134.4 (d), 133.8 (d), 131.8 (d), 129.9 (d), 114.4 (t), 111.5 (t), 80.4 (s), 77.4 (s), 67.8 (s), 53.5 (s), 52.4 (d), 50.41 (d), 40.1 (d), 39.3 (t), 32.4 (t), 18.4 (q), 17.7 (q); EIMS *m*/*z* (% relative intensity): 296 $[M]^+$ (2), 214 (10), 196 (11), 148 (100), 133 (80), 121 (36), 105 (40), 93 (90), 79 (54), ESI–HRMS (*m*/*z*): $[M + Na]^+$ calcd for $C_{20}H_{24}O_2Na$, 319.1674; found, 319.1683.

References

1. Danheiser, R. L.; Martinez-Davilla, C.; Sard, H. *Tetrahedron* **1981**, *37,* 3943–3950. doi:10.1016/S0040-4020(01)93268-5
2. Ji, X.; Wang, Q.; Goeke, A. *Chem. Commun*. **2010**, *46*, 8845–8847. doi:10.1039/c0cc02694h
3. Danheiser, R. L.; Gee, S. K.; Sard, H. *J. Am. Chem. Soc.* **1982**, *104*, 7670–7672. doi:10.1021/ja00390a054
4. Rey, M.; Dunkelblum, E.; Allain, R.; Dreiding, A. S. *Helv. Chim. Acta* **1970**, *53,* 2159–2175. doi:10.1002/hlca.19700530829
5. Brady, W. T.; Parry, F. H., III.; Roe, R., Jr.; Hoff, E. F., Jr. *Tetrahedron Lett.* **1970**, *11*, 819–822. doi:10.1016/S0040-4039(01)97840-2
