# Supplementary material for: Alkoxide-induced ring opening of bicyclic 2-vinylcyclobutanones: A convenient synthesis of 2-vinyl-substituted 3-cycloalkene-1-carboxylic acid esters
Source: Beilstein J Org Chem. 2012 Apr 26;8:650–7. doi: 10.3762/bjoc.8.72 (PMC3388850; doi:10.3762/bjoc.8.72)
Supplement: File 2 — NMR spectral data for unknown compounds. [file Beilstein_J_Org_Chem-08-650-s002.pdf]

Supporting Information

for

Alkoxide-induced ring opening of bicyclic 2-vinylcyclobutanones: A convenient synthesis of 2-vinyl-substituted 3-cycloalkene-1-carboxylic acid esters

Xiufang Ji[1], Zhiming Li[1], Quanrui Wang*[1] and Andreas Goeke*[2]

Address: [1]Department of Chemistry, Fudan University, 220 Handan Road, 200433 Shanghai, P. R. China and [2]Shanghai Givaudan Ltd., Fragrances, 298 Li Shi Zhen Road, 201203 Shanghai, P. R. China

Email: Quanrui Wang* - qrwang@fudan.edu.cn; Andreas Goeke* - andreas.goeke@givaudan.com

*Corresponding author

NMR spectral data for unknown compounds

Spectra of compound **4b**……S2

Spectra of compound **4d**……S3

Spectra of compound **4g**……S4

Spectra of compound **4i**……S6

Spectra of compound **5a–5i**……S7

Spectra of compound **6a–6i**……S16

Spectra of compound **7**……S25

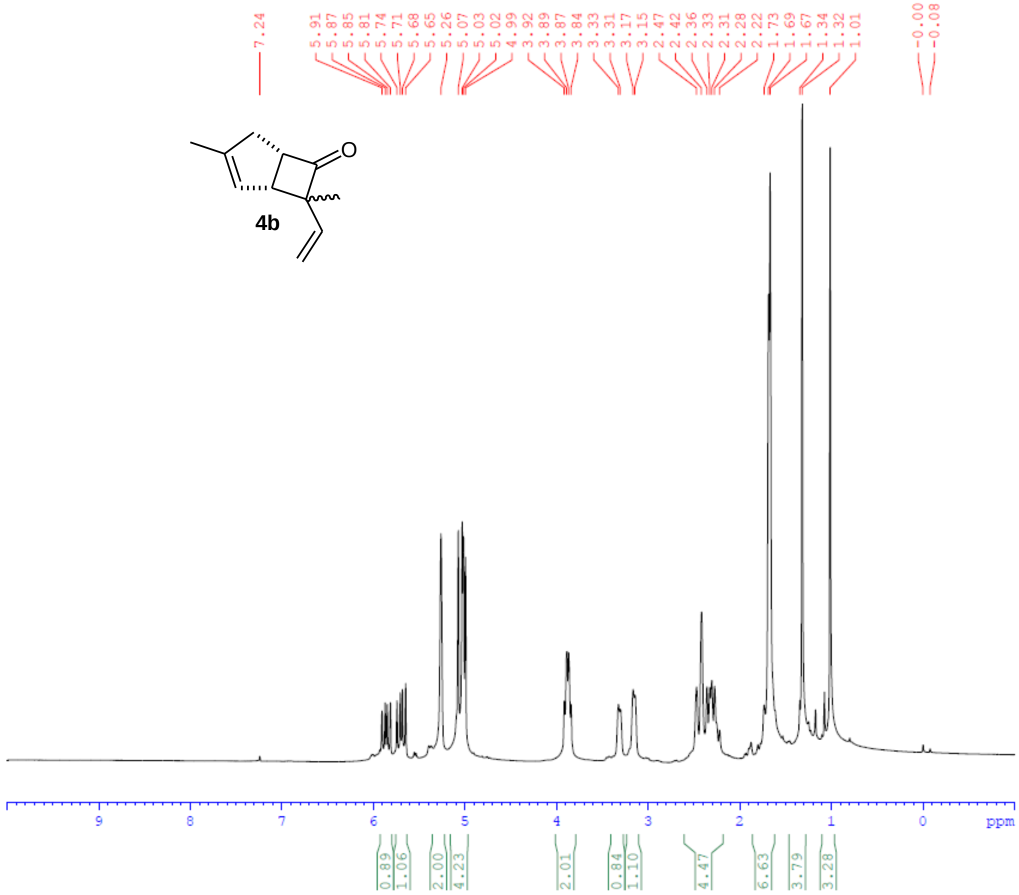
7.24
5.91 5.87 5.85 5.81 5.74 5.71 5.68 5.65 5.26 5.07 5.03 5.02 4.99 3.92 3.89 3.87 3.84 3.33 3.31 3.17 3.15 2.47 2.42 2.36 2.33 2.31 2.28 2.22 1.73 1.69 1.67 1.34 1.32 1.01
-0.00 -0.08
O
4b
9 8 7 6 5 4 3 2 1 0 ppm
0.89 1.06 2.00 4.23 2.01 0.84 1.10 4.47 6.63 3.79 3.28

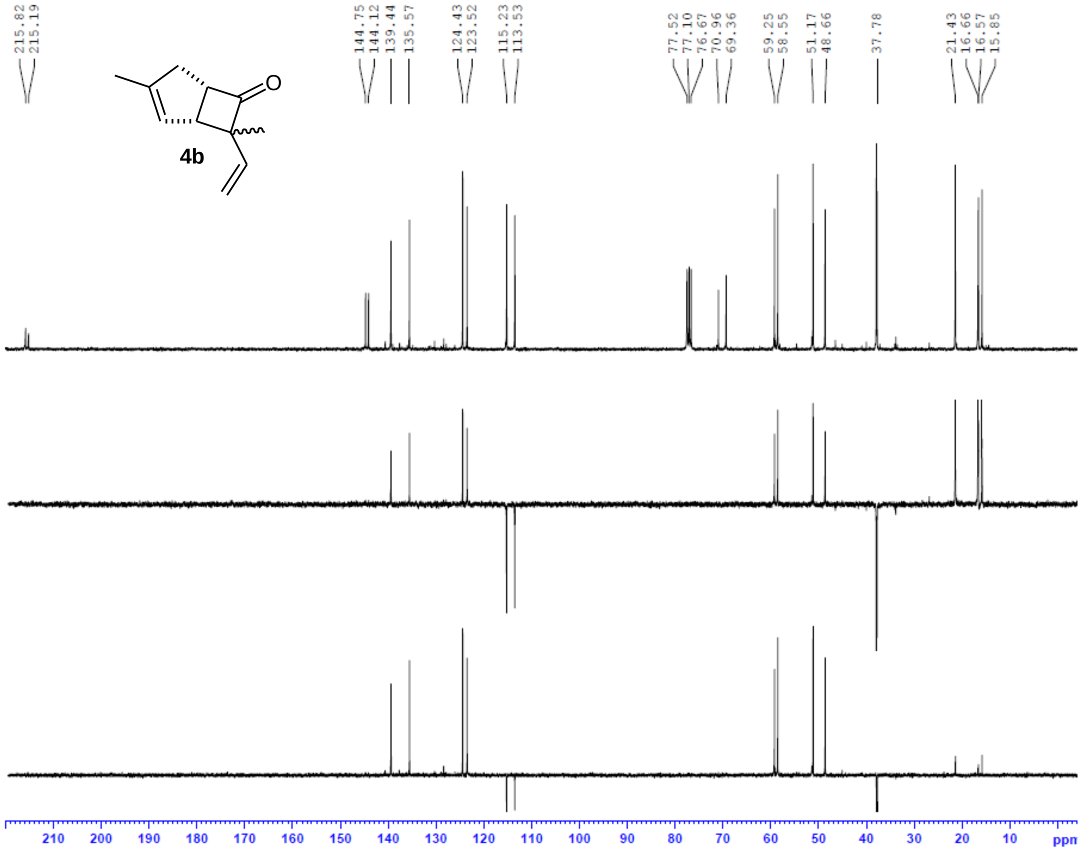
215.82 215.19
144.75 144.12 139.44 135.57 124.43 123.52 115.23 113.53
77.52 77.10 76.67 70.96 69.36 59.25 58.55 51.17 48.66 37.78 21.43 16.66 16.57 15.85
O
4b
210 200 190 180 170 160 150 140 130 120 110 100 90 80 70 60 50 40 30 20 10 ppm

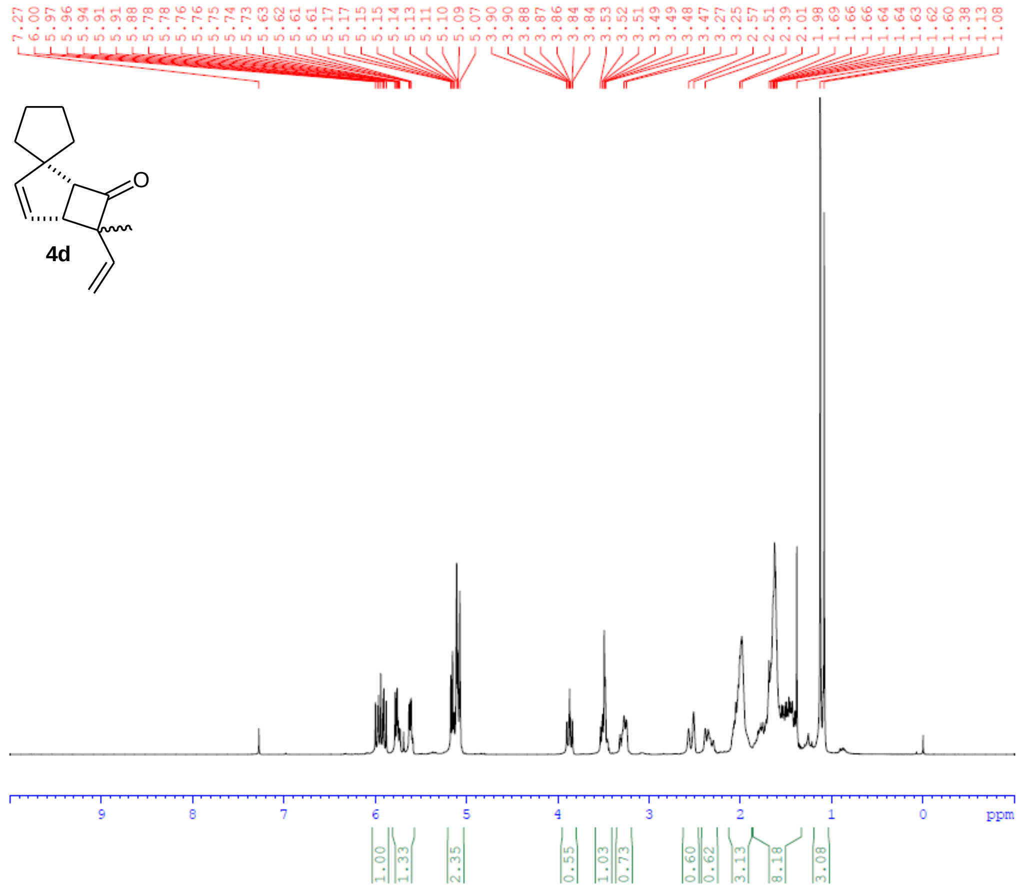
7.27 6.00 5.97 5.96 5.94 5.91 5.91 5.88 5.78 5.78 5.76 5.76 5.75 5.74 5.73 5.63 5.62 5.61 5.61 5.17 5.17 5.15 5.15 5.14 5.13 5.11 5.10 5.09 5.07 3.90 3.90 3.88 3.87 3.86 3.84 3.84 3.53 3.52 3.51 3.49 3.49 3.48 3.47 3.27 3.25 2.57 2.51 2.39 2.01 1.98 1.69 1.66 1.66 1.64 1.64 1.63 1.62 1.60 1.38 1.13 1.08
4d
9
8
7
6
5
4
3
2
1
0
ppm
1.00
1.33
2.35
0.55
1.03
0.73
0.60
0.62
3.13
8.18
3.08

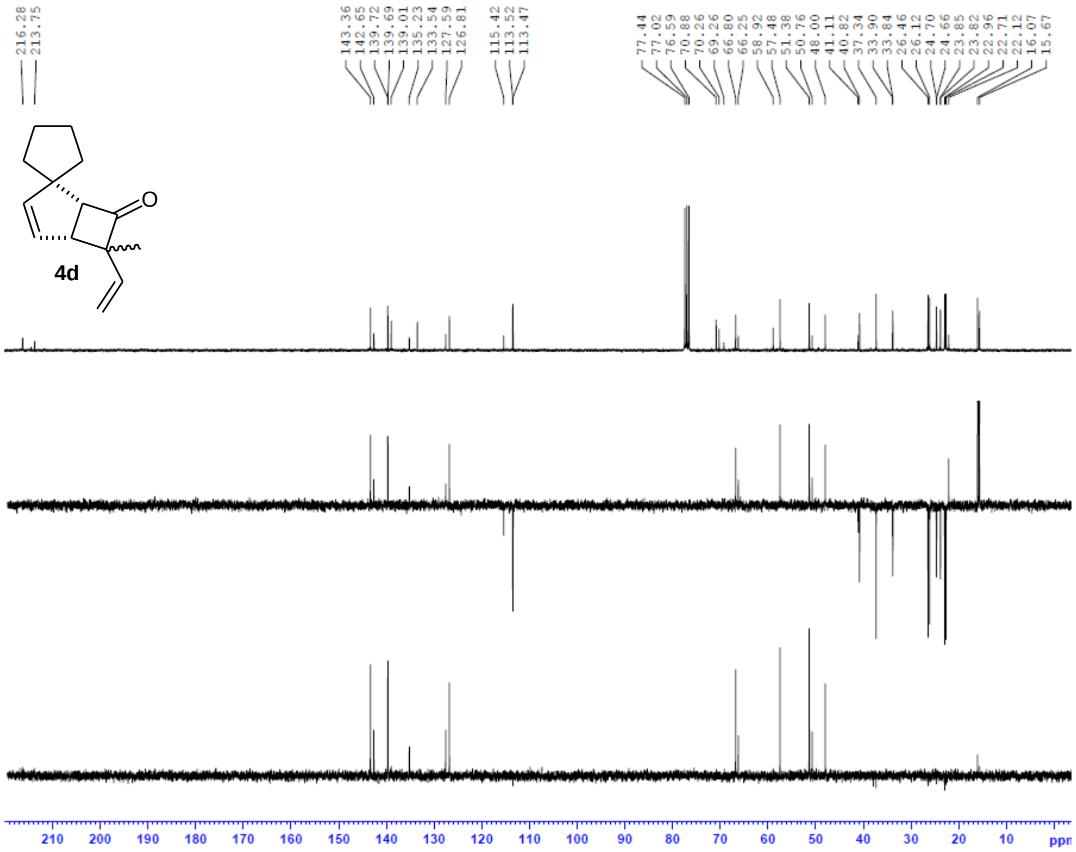
216.28 213.75
143.36 142.65 139.72 139.69 139.01 135.23 133.54 127.59 126.81
115.42 113.52 113.47
77.44 77.02 76.59 70.88 70.26 69.26 66.80 66.25 58.92 57.48 51.38 50.76 48.00 41.11 40.82 37.34 33.90 33.84 26.46 26.12 24.70 24.66 23.85 23.82 22.96 22.71 22.12 16.07 15.67
4d
210
200
190
180
170
160
150
140
130
120
110
100
90
80
70
60
50
40
30
20
10
ppm

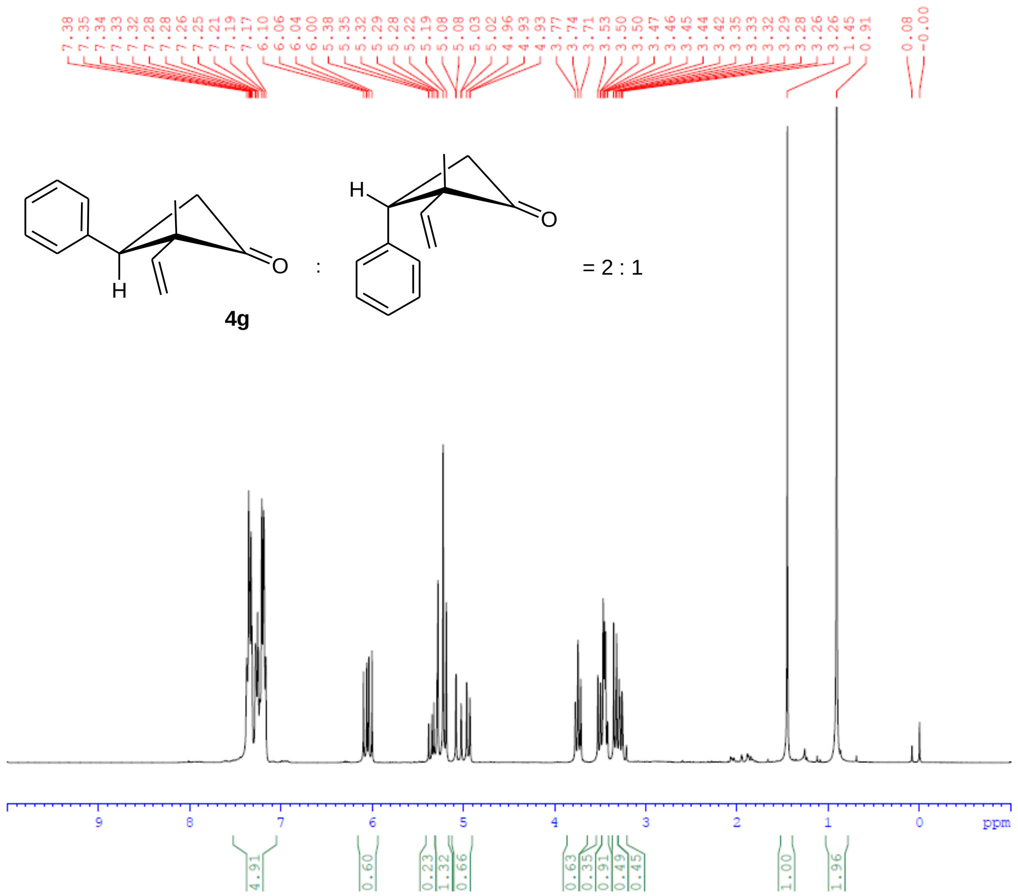
4g
= 2 : 1

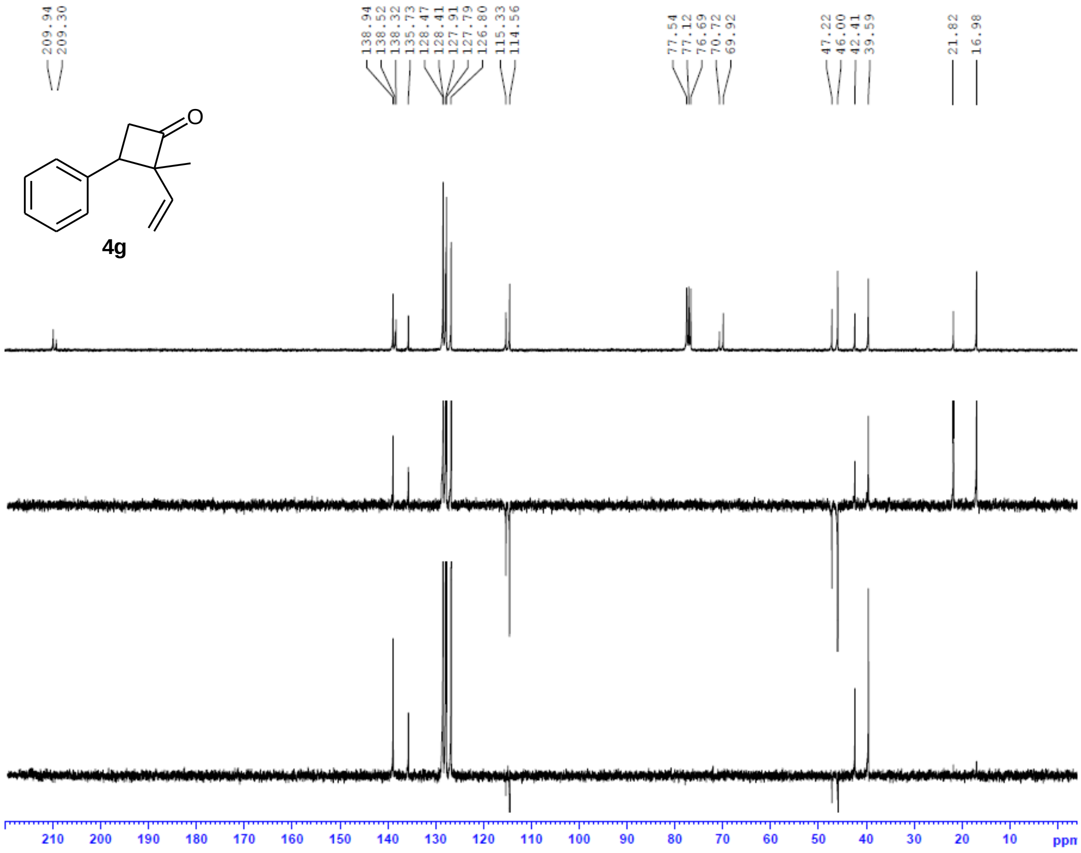
4g

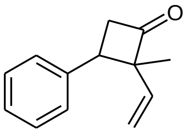

4g

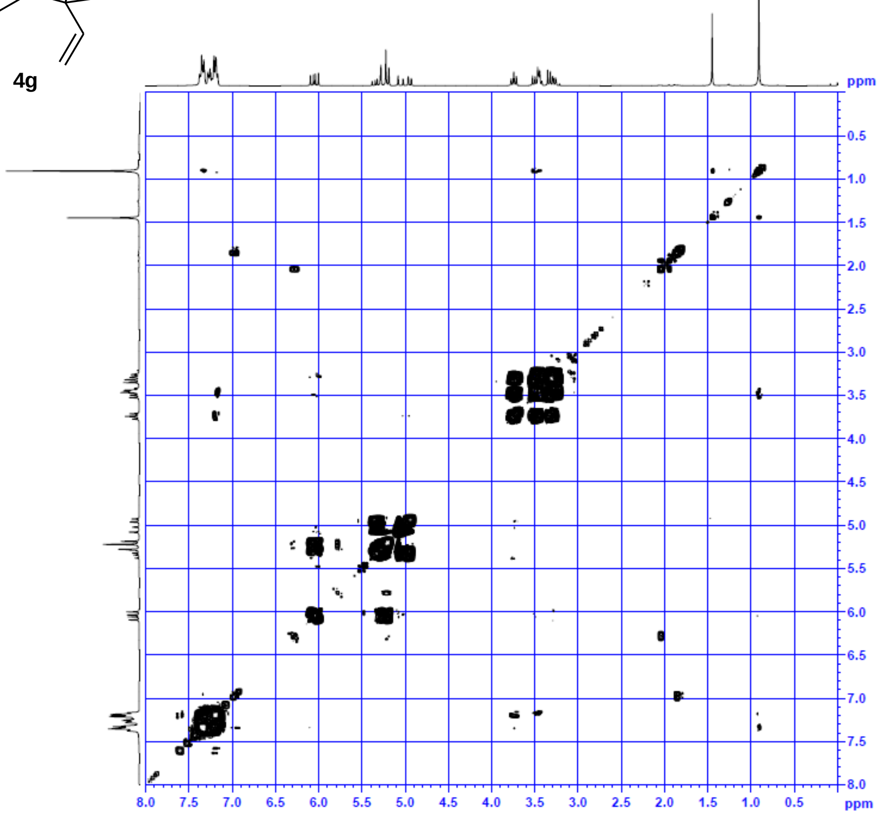

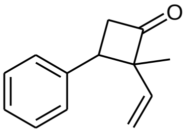

4g

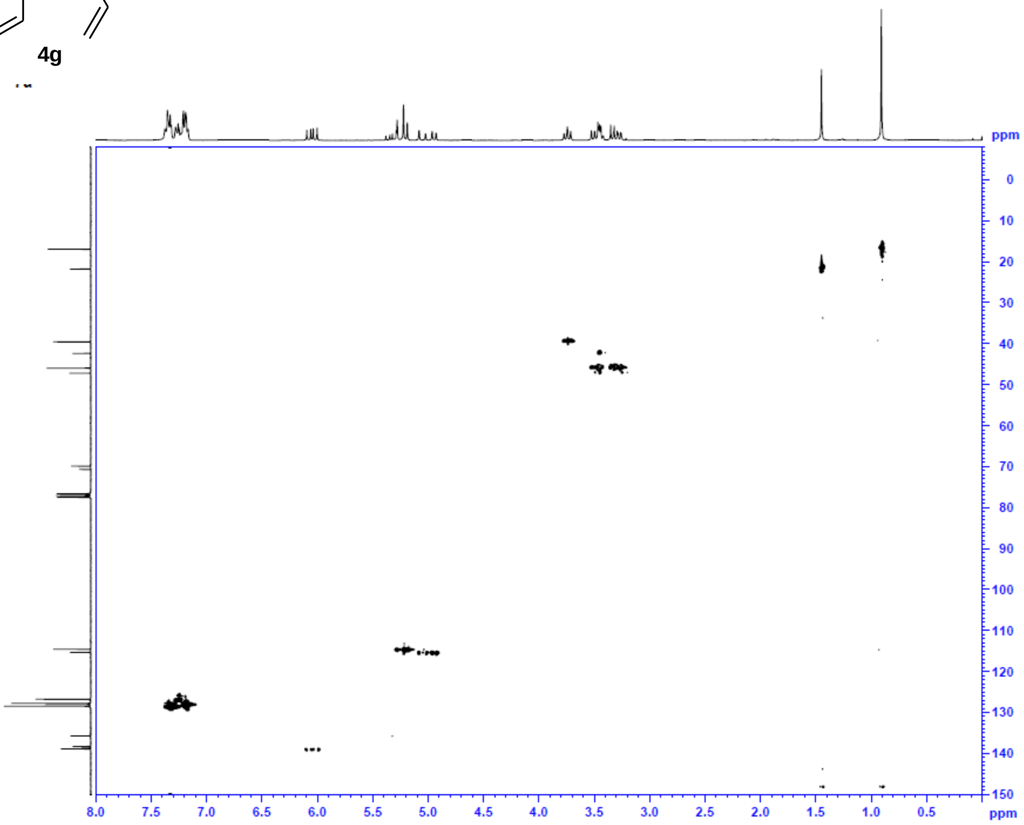

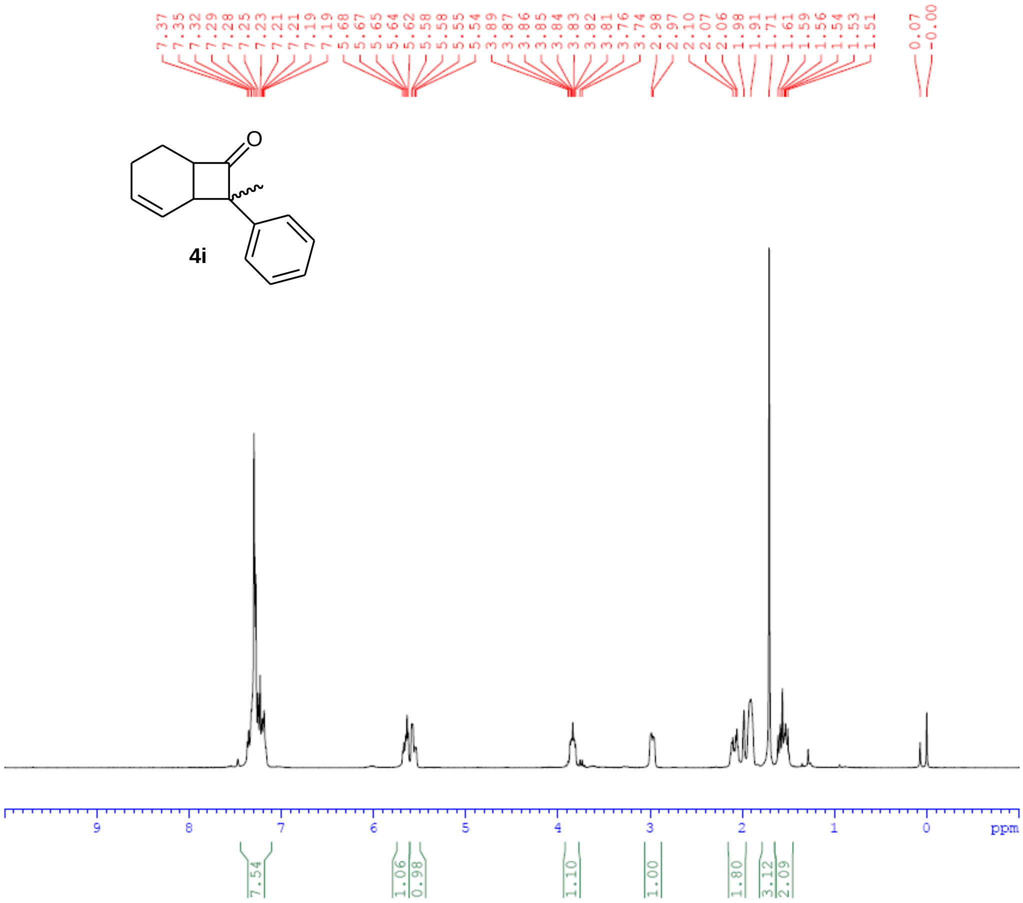
4i

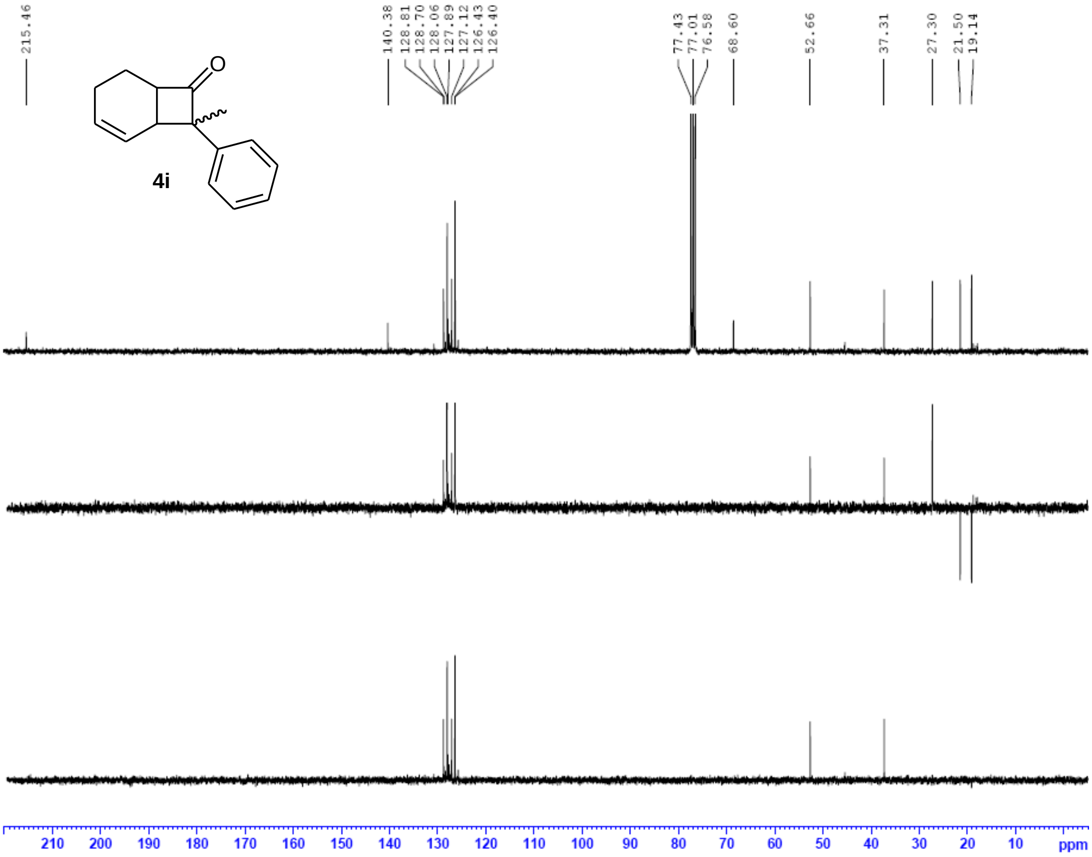
4i

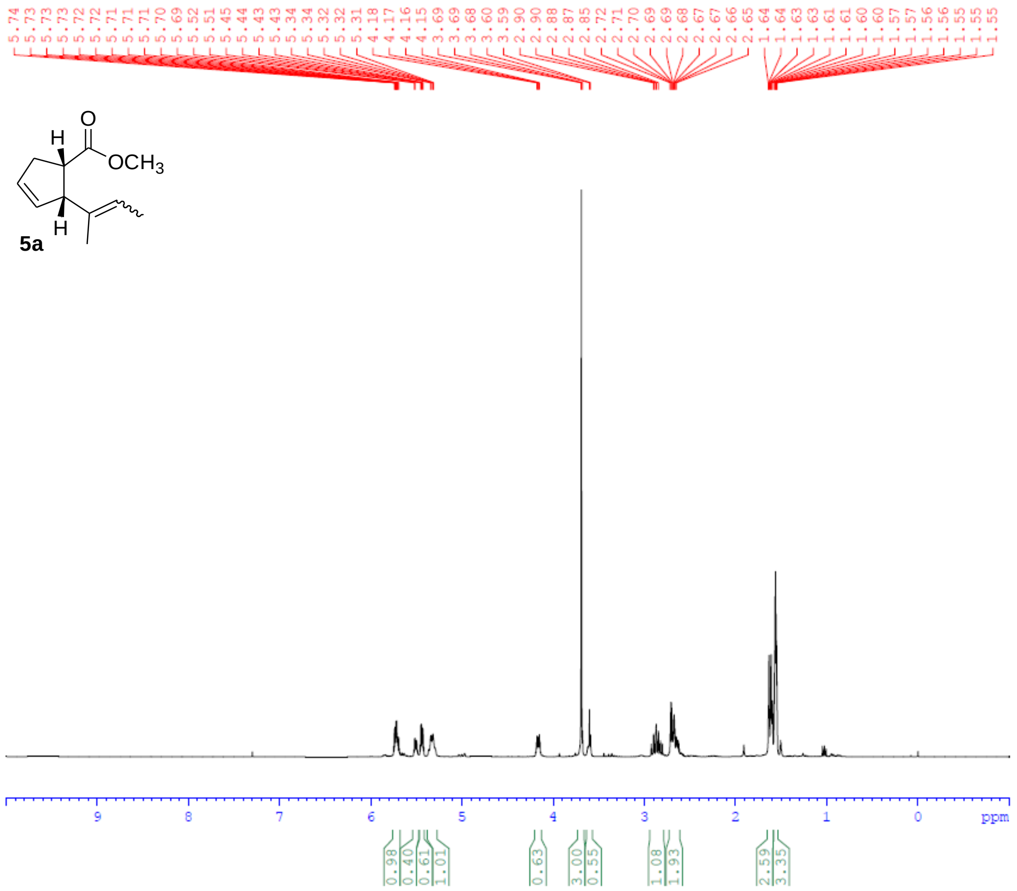

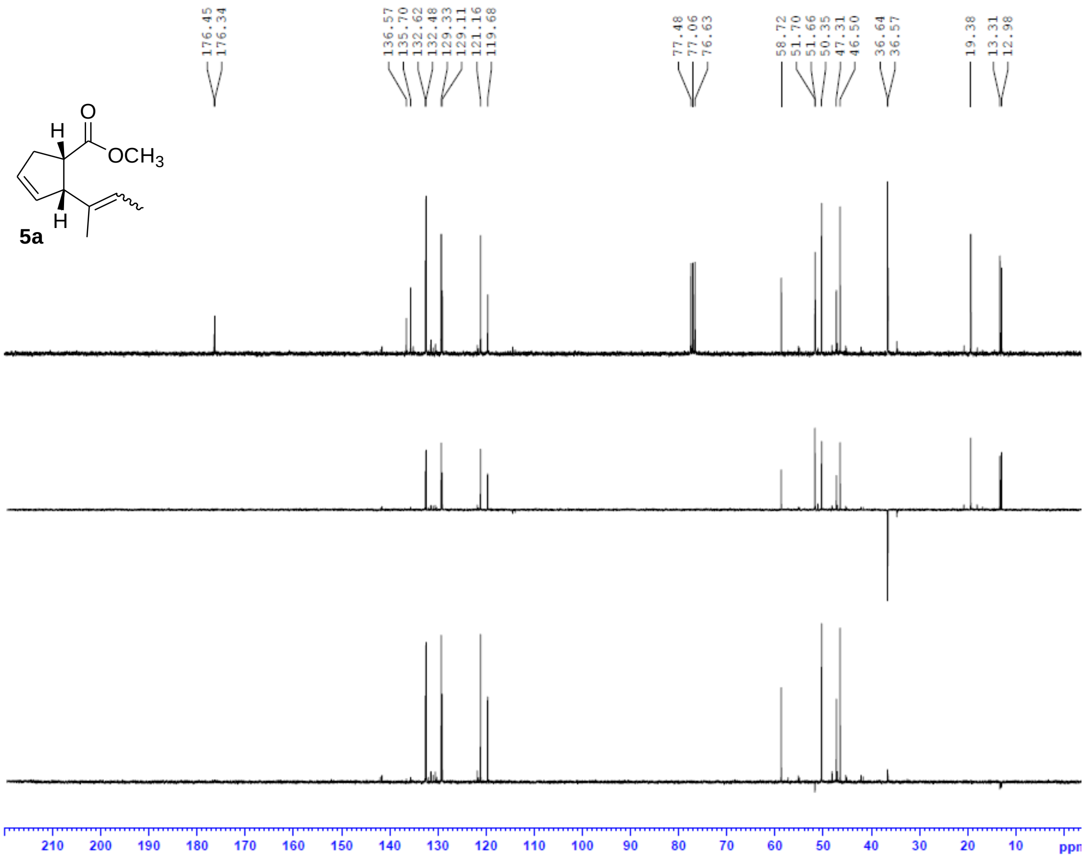

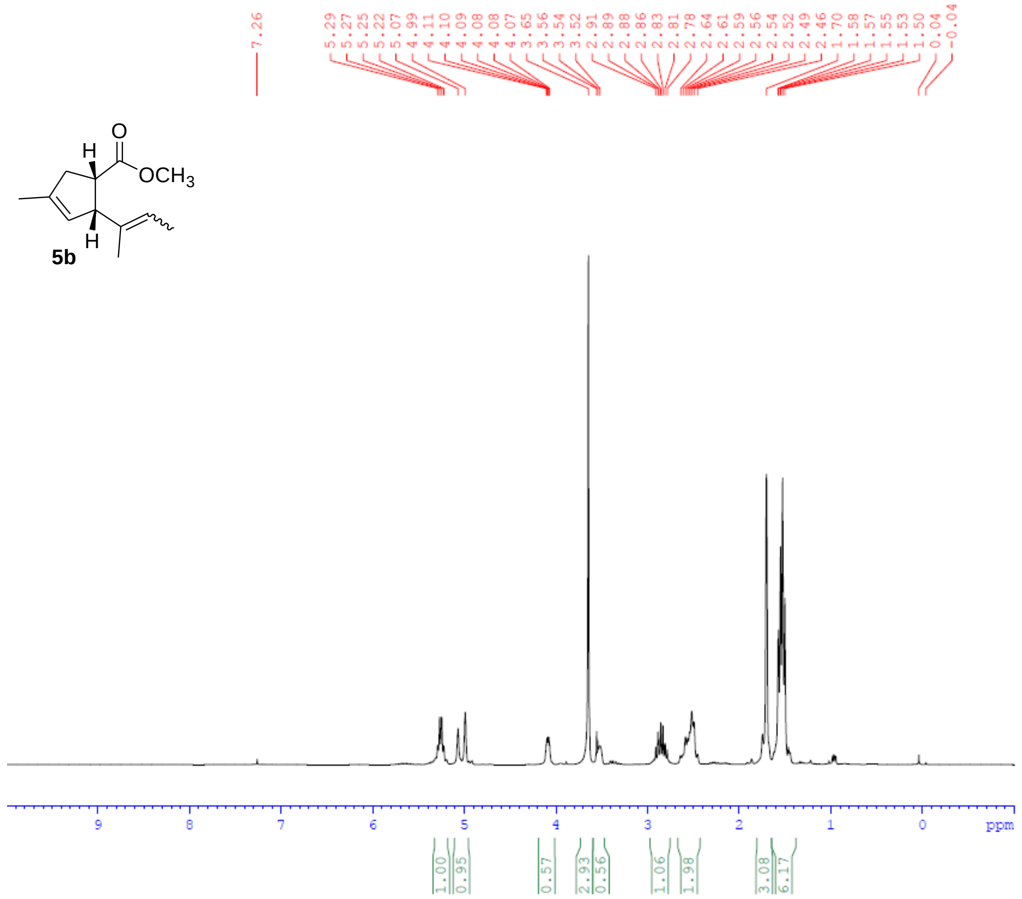
7.26
5.29
5.27
5.25
5.22
5.07
4.99
4.11
4.10
4.09
4.08
4.08
4.07
3.65
3.56
3.54
3.52
2.91
2.89
2.88
2.86
2.83
2.81
2.78
2.64
2.61
2.59
2.56
2.54
2.52
2.49
2.46
1.70
1.58
1.57
1.55
1.53
1.50
0.04
-0.04
O
H
OCH3
H
5b
9
8
7
6
5
4
3
2
1
0
ppm
1.00
0.95
0.57
2.93
0.56
1.06
1.98
3.08
6.17

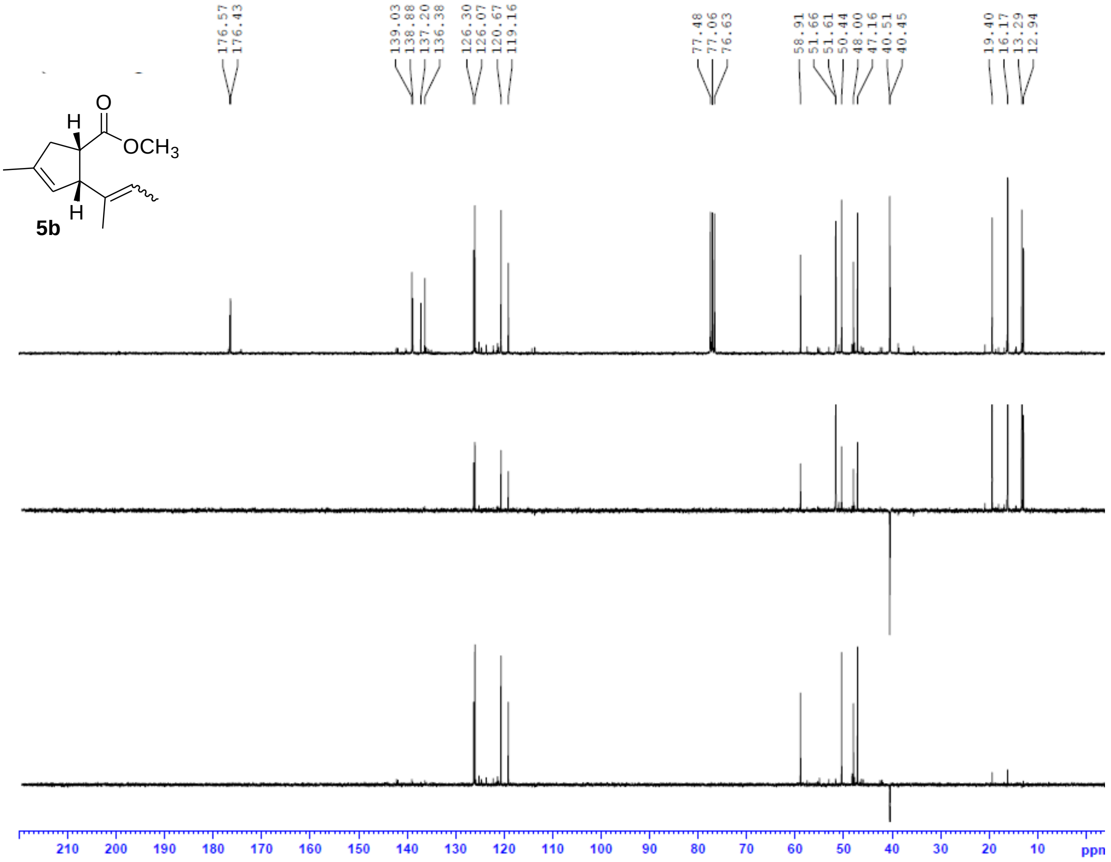
176.57
176.43
139.03
138.88
137.20
136.38
126.30
126.07
120.67
119.16
77.48
77.06
76.63
58.91
51.66
51.61
50.44
48.00
47.16
40.51
40.45
19.40
16.17
13.29
12.94
O
H
OCH3
H
5b
210
200
190
180
170
160
150
140
130
120
110
100
90
80
70
60
50
40
30
20
10
ppm

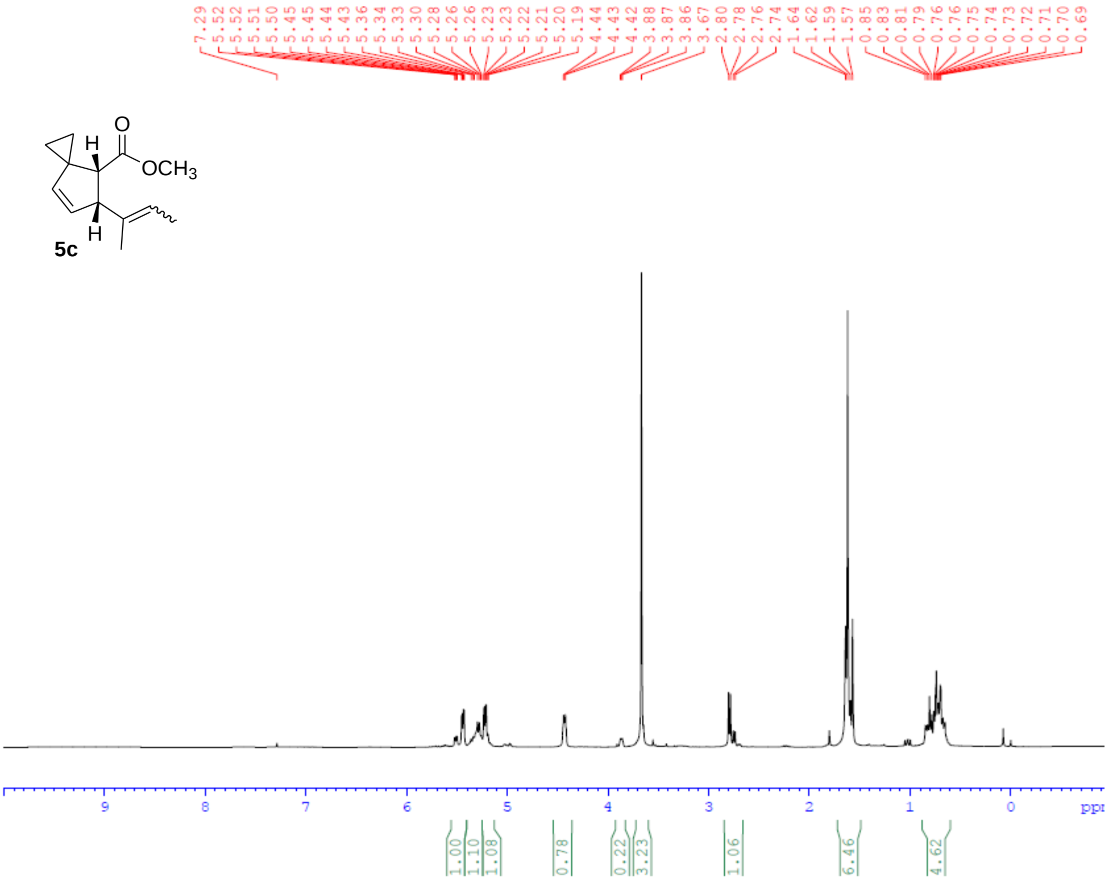
7.29 5.52 5.52 5.51 5.50 5.45 5.45 5.44 5.43 5.36 5.34 5.33 5.30 5.28 5.26 5.26 5.23 5.23 5.22 5.21 5.20 5.19 4.44 4.43 4.42 3.88 3.87 3.86 3.67 2.80 2.78 2.76 2.74 1.64 1.62 1.59 1.57 0.85 0.83 0.81 0.79 0.76 0.76 0.75 0.74 0.73 0.72 0.71 0.70 0.69
O
H
OCH3
H
5c
9 8 7 6 5 4 3 2 1 0 ppm
1.00 1.10 1.08 0.78 0.22 3.23 1.06 6.46 4.62

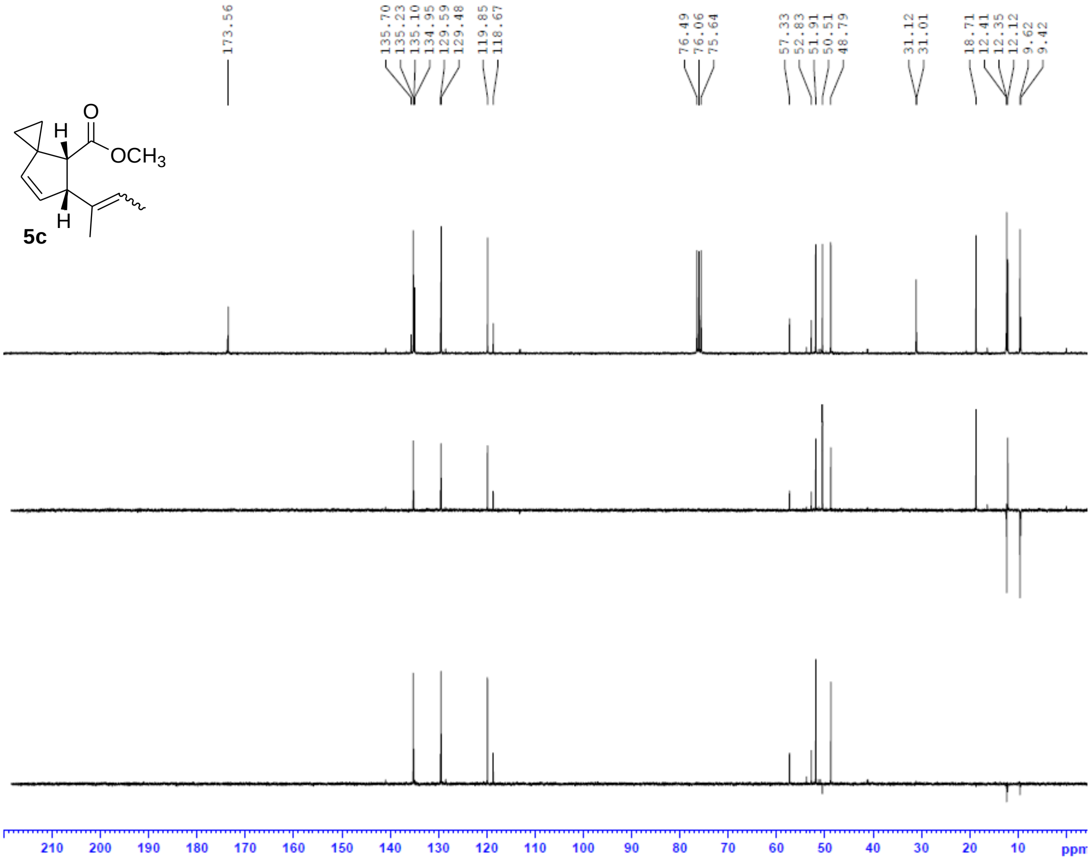
173.56
135.70 135.23 135.10 134.95 129.59 129.48 119.85 118.67
76.49 76.06 75.64
57.33 52.83 51.91 50.51 48.79
31.12 31.01
18.71 12.41 12.35 12.12 9.62 9.42
O
H
OCH3
H
5c
210 200 190 180 170 160 150 140 130 120 110 100 90 80 70 60 50 40 30 20 10 ppm

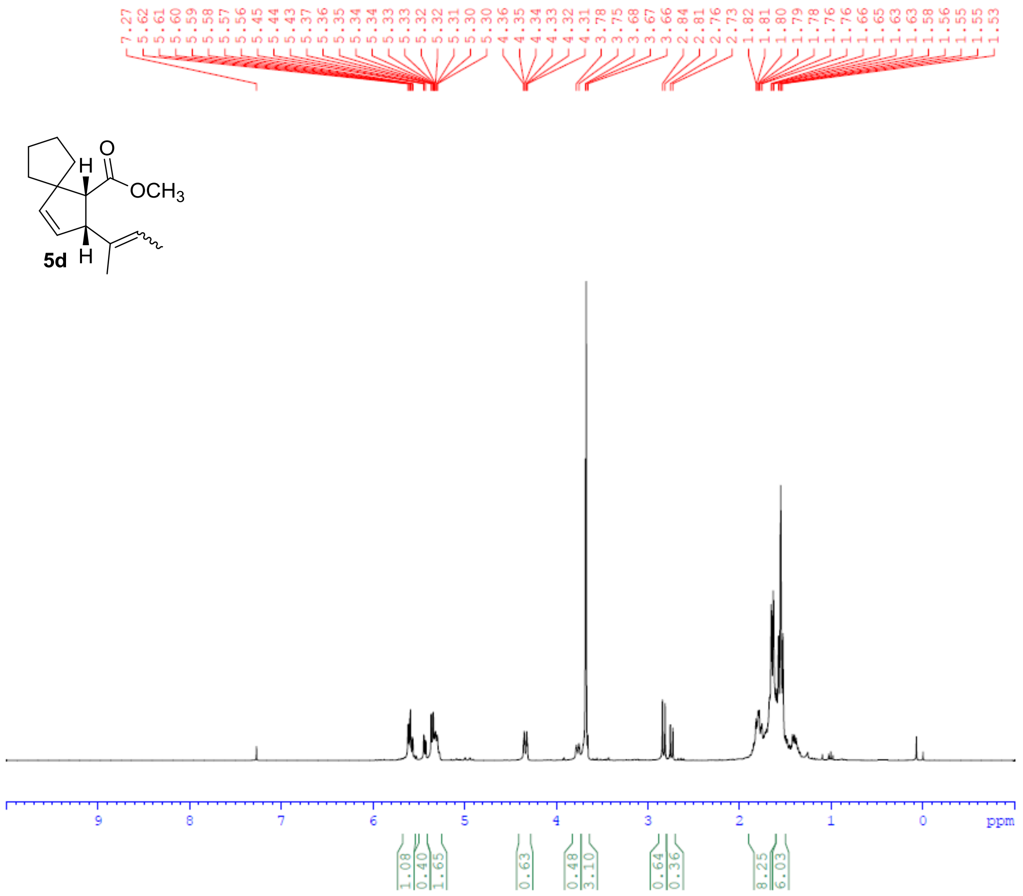
7.27 5.62 5.61 5.60 5.59 5.58 5.57 5.56 5.45 5.44 5.43 5.37 5.36 5.35 5.34 5.34 5.33 5.33 5.32 5.32 5.31 5.30 5.30 4.36 4.35 4.34 4.33 4.32 4.31 3.78 3.75 3.68 3.67 3.66 2.84 2.81 2.76 2.73 1.82 1.81 1.80 1.79 1.78 1.76 1.66 1.65 1.63 1.63 1.58 1.56 1.55 1.55 1.53
H
O
OCH3
5d
H
9 8 7 6 5 4 3 2 1 0 ppm
1.08 0.40 1.65 0.63 0.48 3.10 0.64 0.36 8.25 6.03

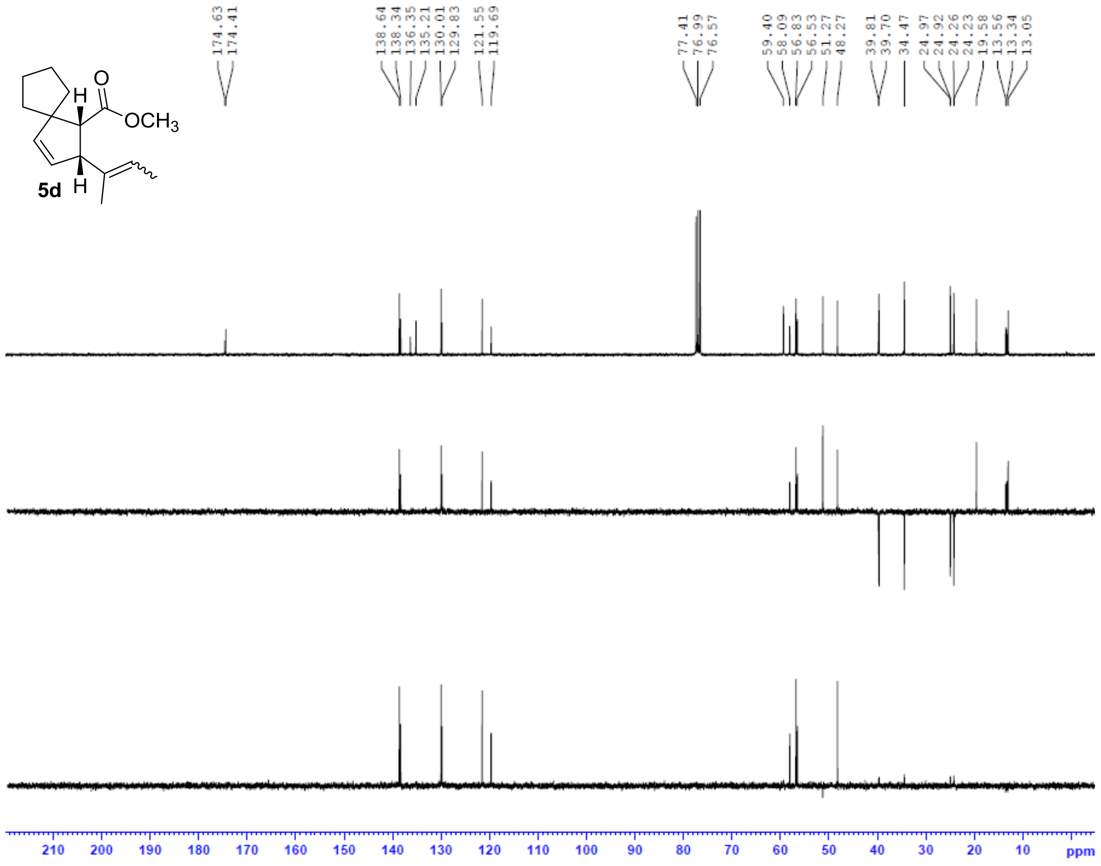
174.63 174.41 138.64 138.34 136.35 135.21 130.01 129.83 121.55 119.69 77.41 76.99 76.57 59.40 58.09 56.83 56.53 51.27 48.27 39.81 39.70 34.47 24.97 24.92 24.26 24.23 19.58 13.56 13.34 13.05
H
O
OCH3
5d
H
210 200 190 180 170 160 150 140 130 120 110 100 90 80 70 60 50 40 30 20 10 ppm

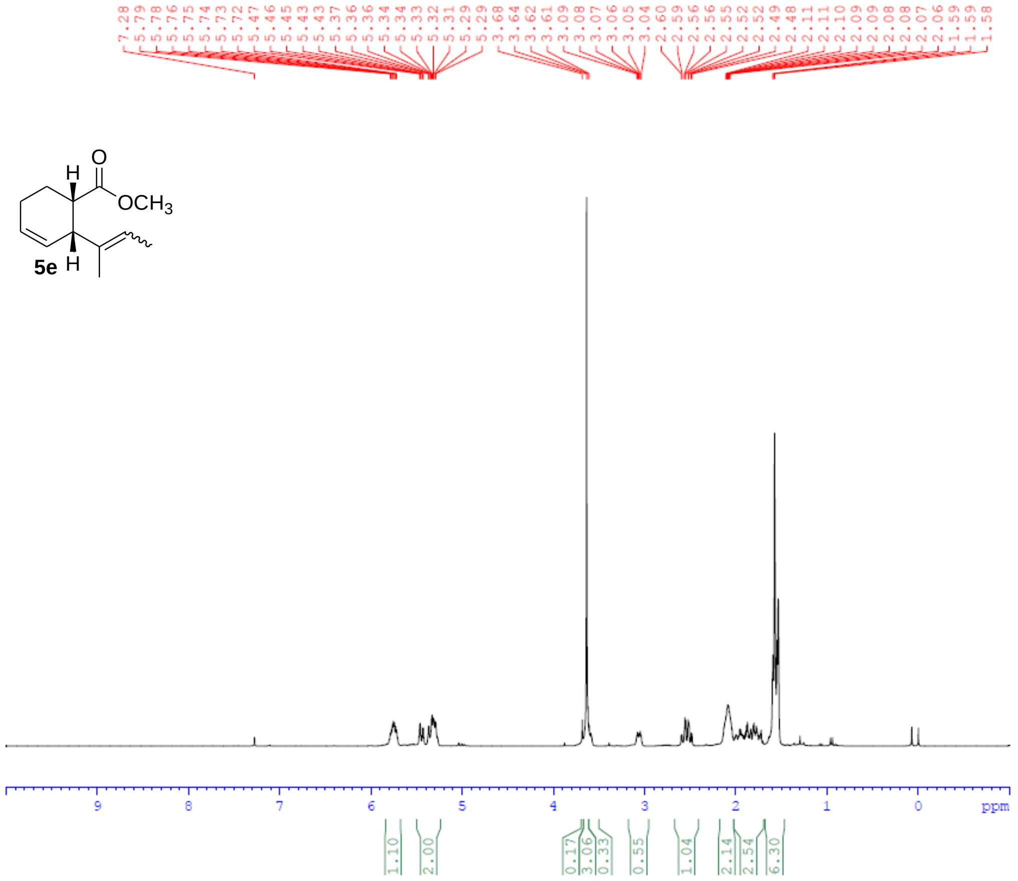
O
H
OCH3
5e
H
ppm

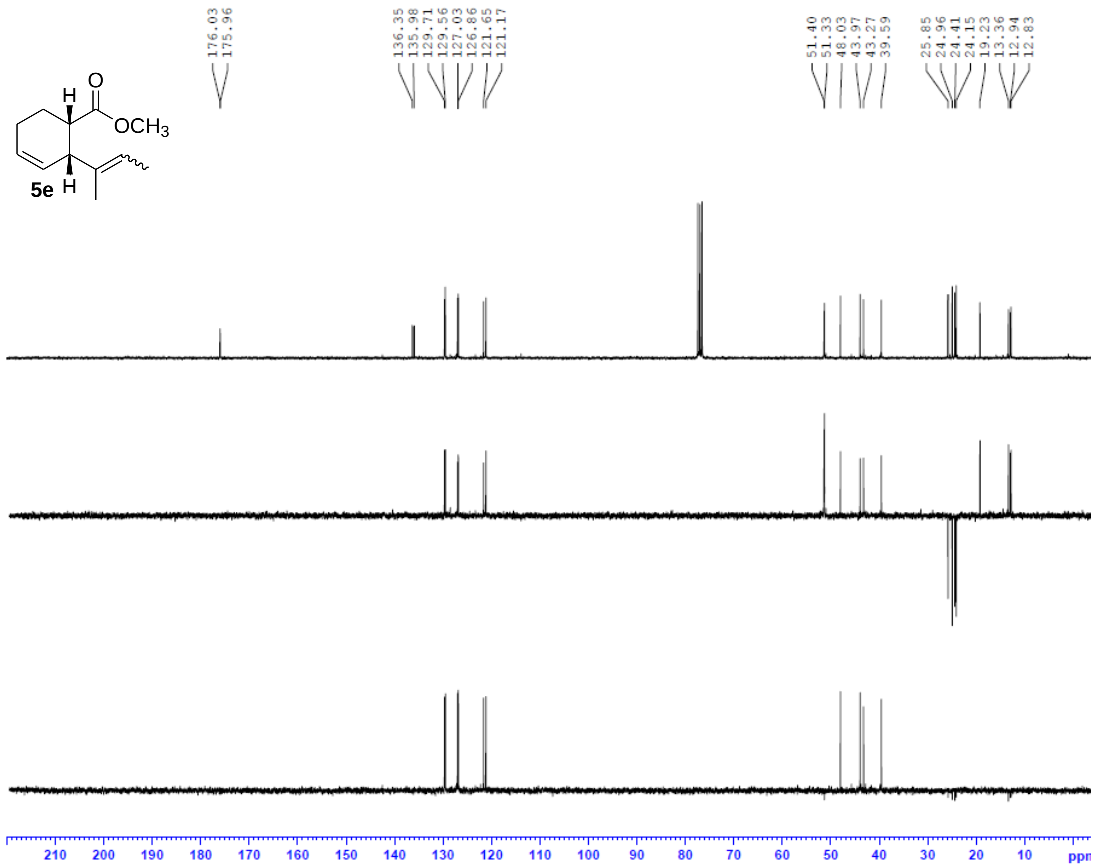
O
H
OCH3
5e
H
ppm

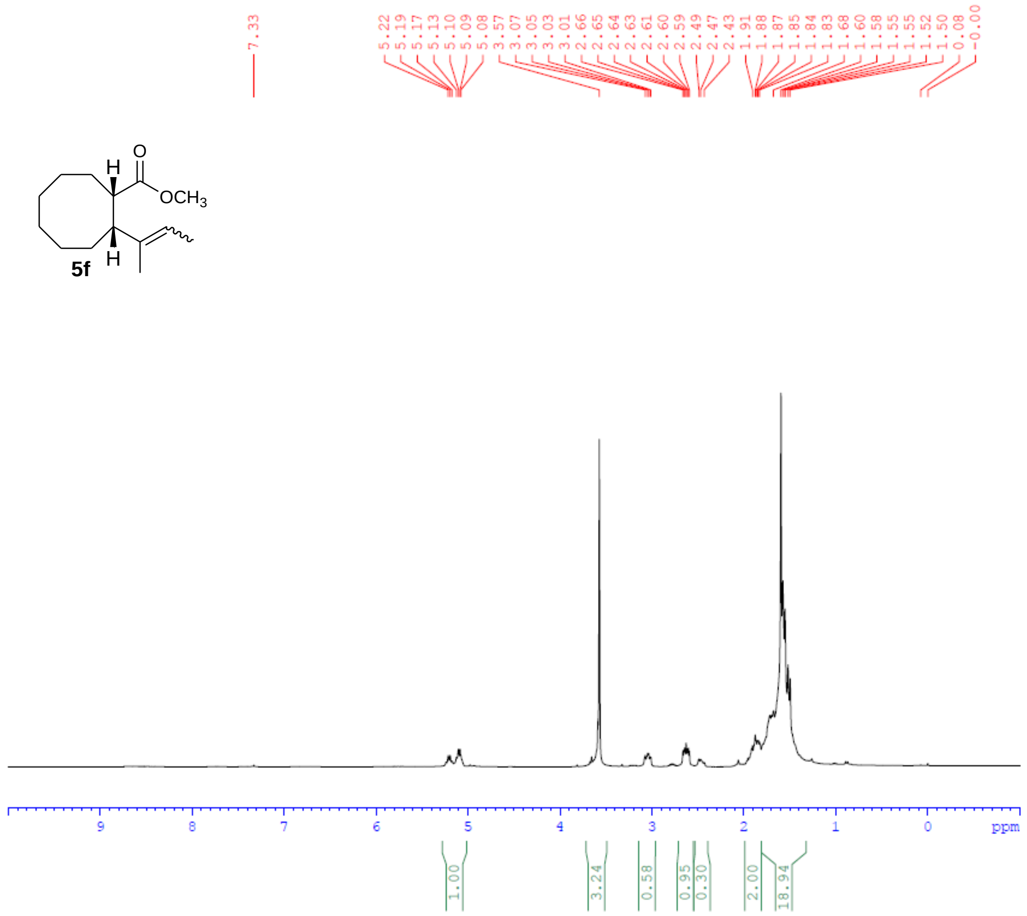
7.33
5.22 5.19 5.17 5.13 5.10 5.09 5.08 3.57 3.07 3.05 3.03 3.01 2.66 2.65 2.64 2.63 2.61 2.60 2.59 2.49 2.47 2.43 1.91 1.88 1.87 1.85 1.84 1.83 1.68 1.60 1.58 1.55 1.52 1.50 0.08 -0.00
H
O
OCH3
H
5f
9 8 7 6 5 4 3 2 1 0 ppm
1.00 3.24 0.58 0.95 0.30 2.00 18.94

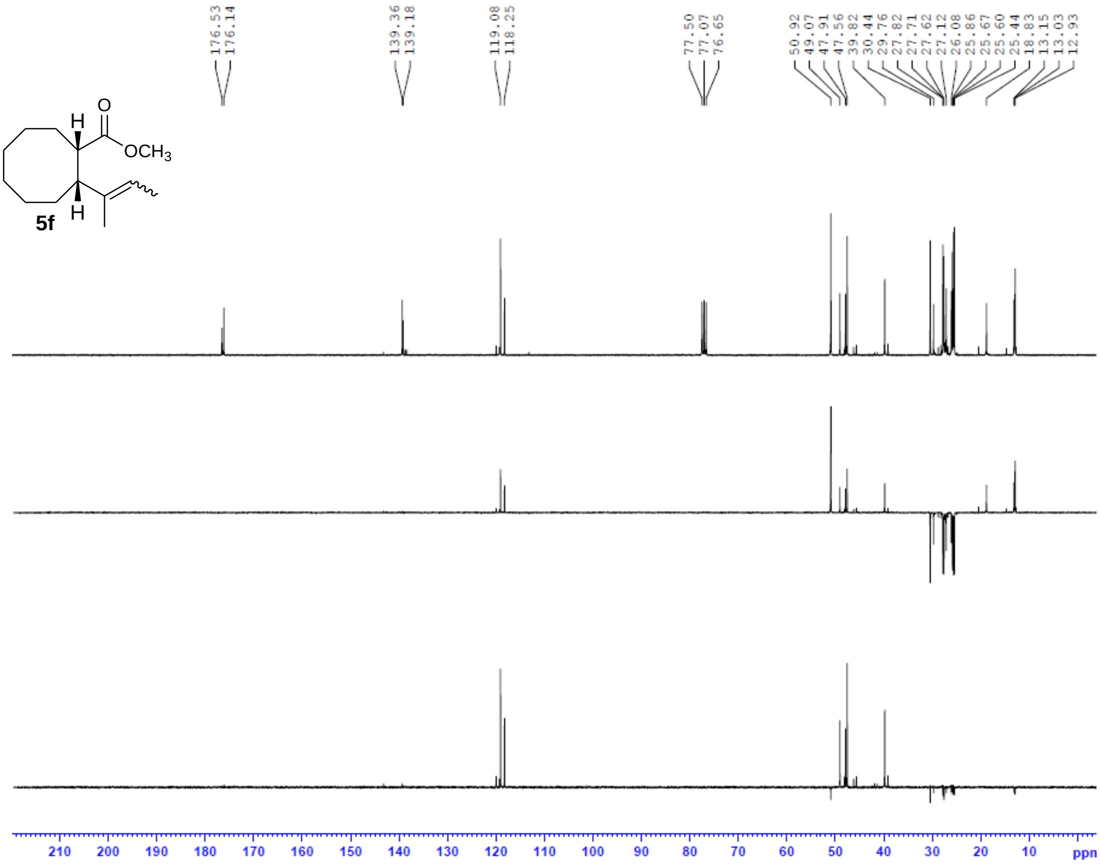
176.53 176.14
139.36 139.18
119.08 118.25
77.50 77.07 76.65
50.92 49.07 47.91 47.56 39.82 30.44 29.76 27.82 27.71 27.62 27.12 26.08 25.86 25.67 25.60 25.44 18.83 13.15 13.03 12.93
H
O
OCH3
H
5f
210 200 190 180 170 160 150 140 130 120 110 100 90 80 70 60 50 40 30 20 10 ppm

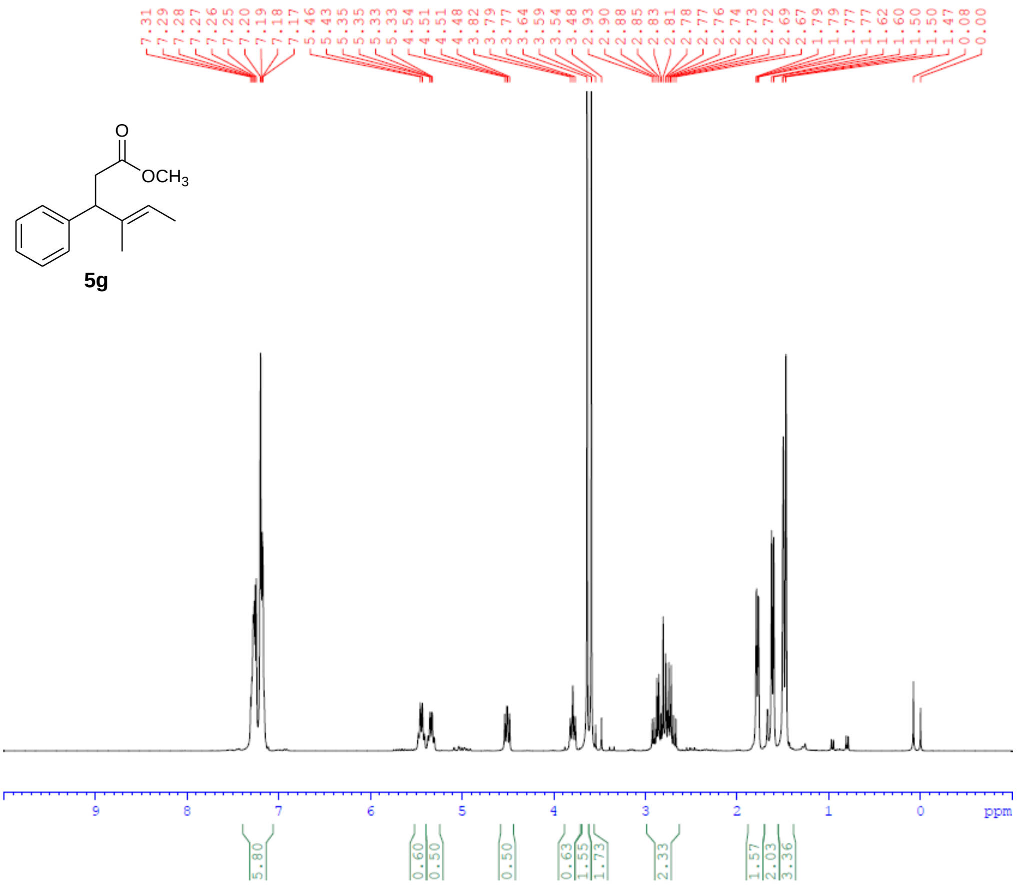

5g

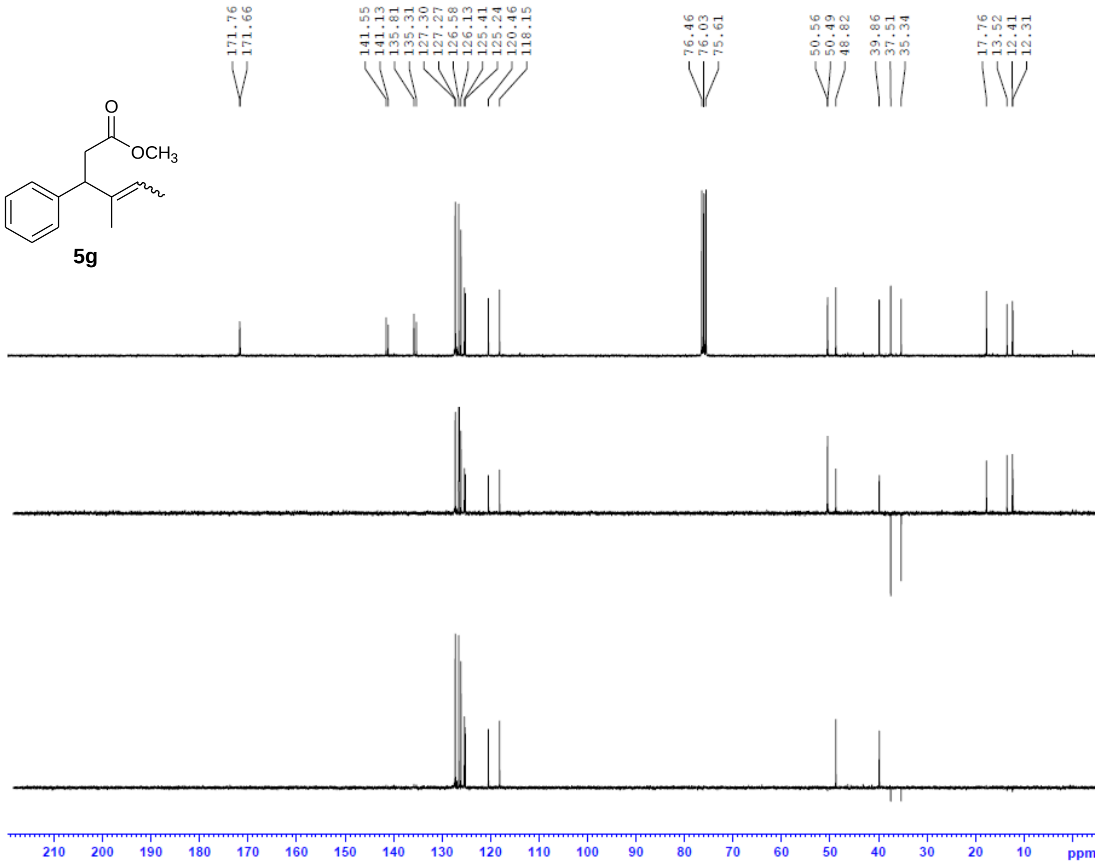

5g

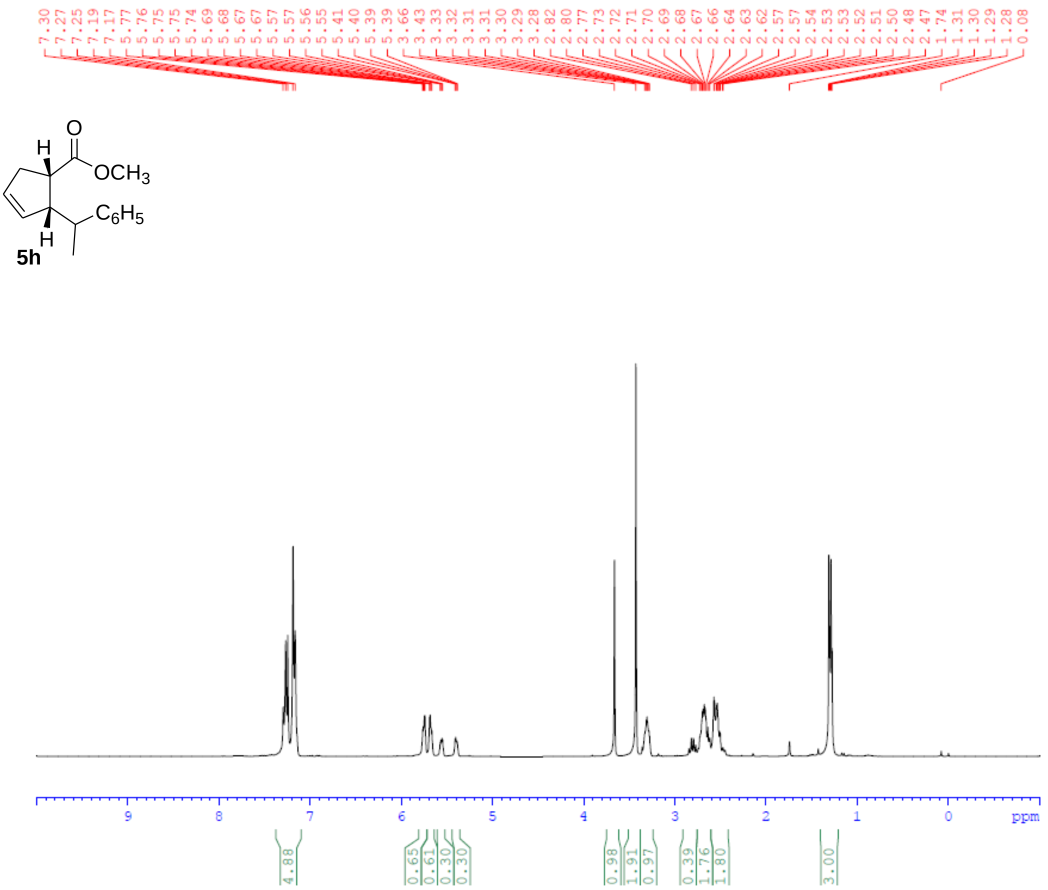
O
H
OCH3
C6H5
H
5h
4.88
0.65
0.61
0.30
0.30
0.98
1.91
0.97
0.39
1.76
1.80
3.00
ppm

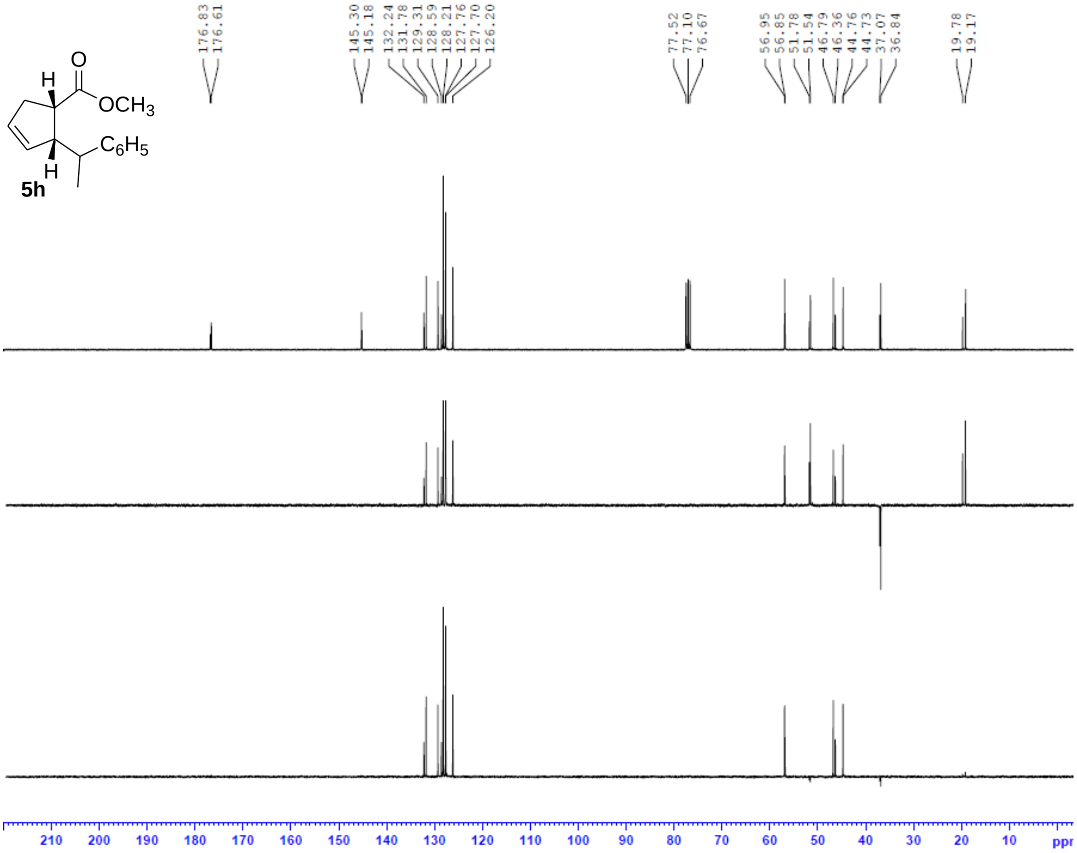
176.83
176.61
145.30
145.18
132.24
131.78
129.31
128.59
128.21
127.76
127.70
126.20
77.52
77.10
76.67
56.95
56.85
51.78
51.54
46.79
46.36
44.76
44.73
37.07
36.84
19.78
19.17
O
H
OCH3
C6H5
H
5h

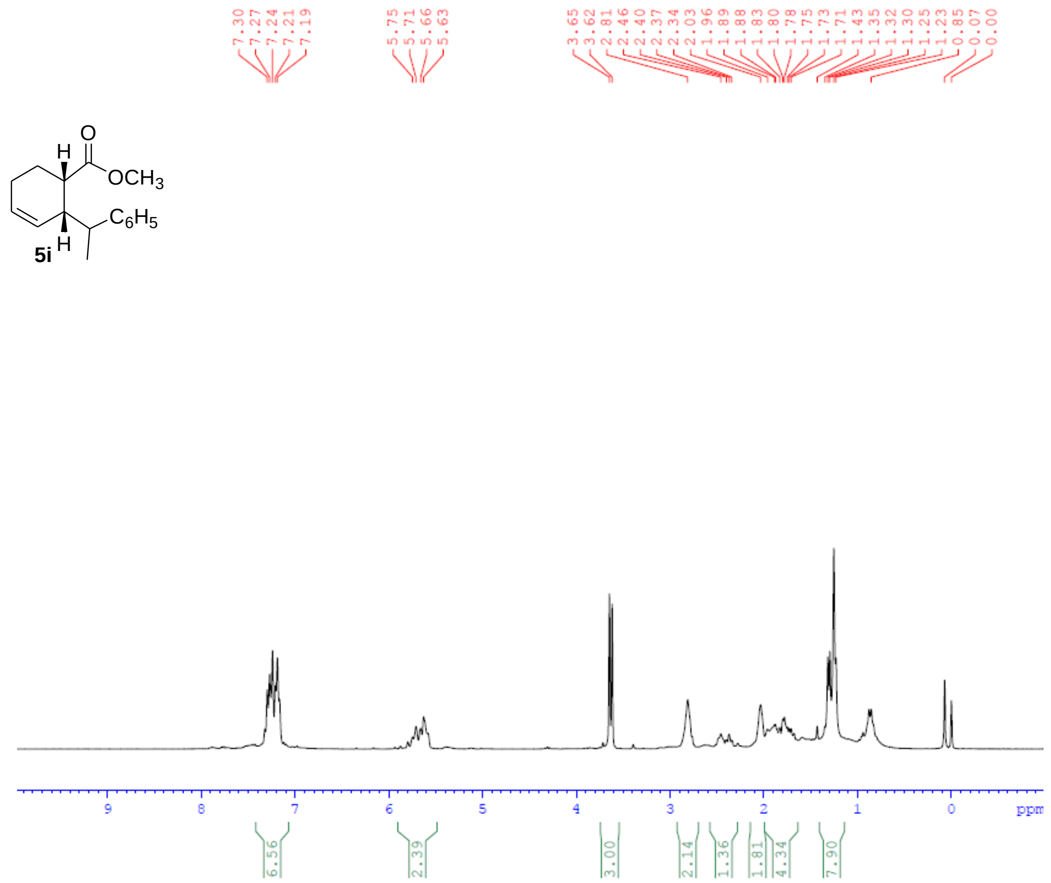

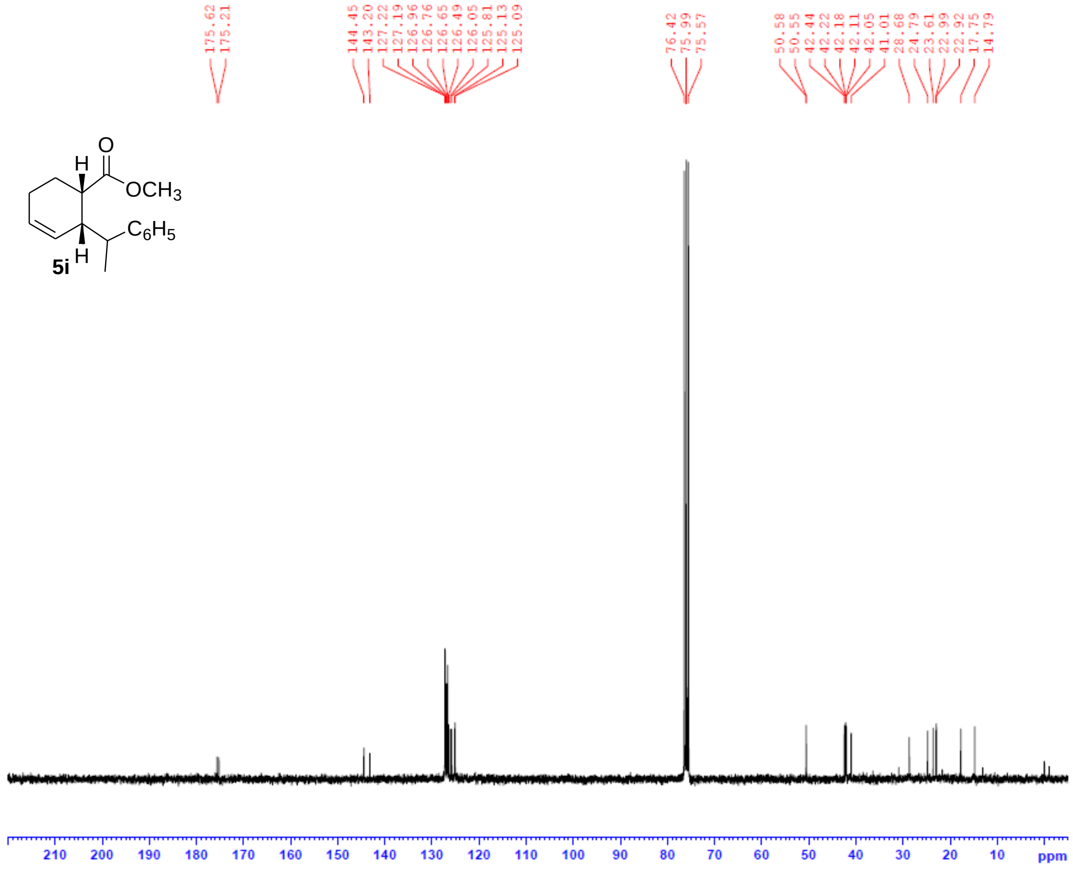

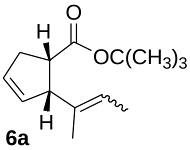
O
H
OC(CH3)3
H

6a

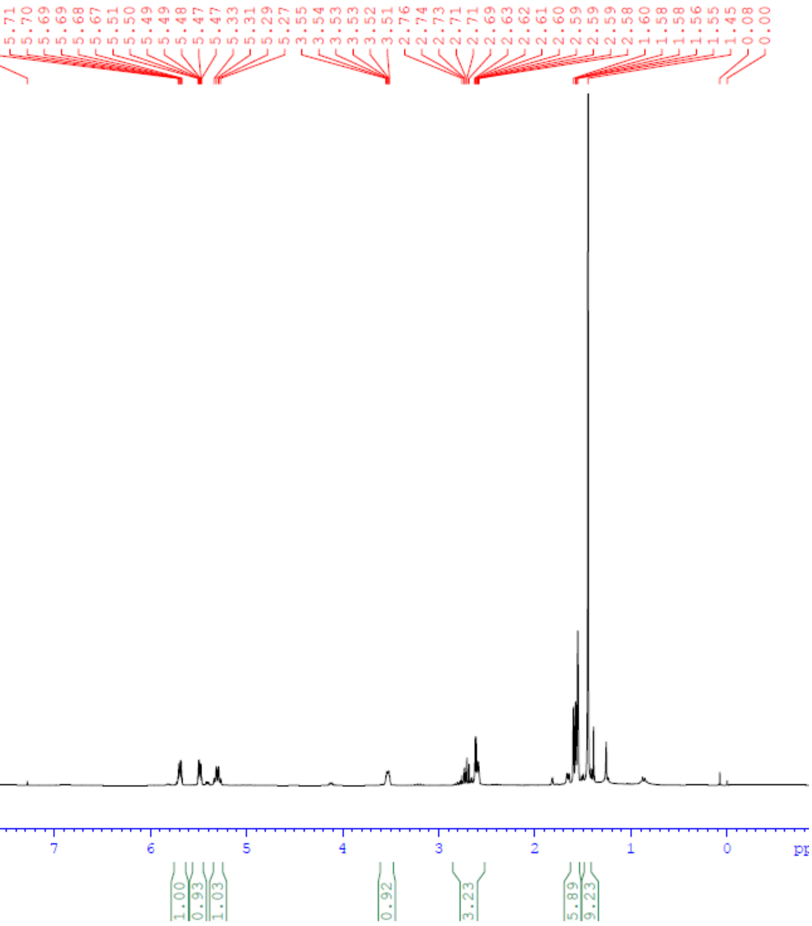
7.28
5.71
5.71
5.70
5.69
5.69
5.68
5.67
5.51
5.50
5.49
5.49
5.48
5.47
5.47
5.33
5.31
5.29
5.27
3.55
3.54
3.53
3.53
3.52
3.51
2.76
2.74
2.73
2.71
2.71
2.69
2.63
2.62
2.61
2.60
2.59
2.59
2.59
2.58
1.60
1.58
1.58
1.56
1.55
1.45
0.08
0.00
9
8
7
6
5
4
3
2
1
0
1.00
0.93
1.03
0.92
3.23
5.89
9.23

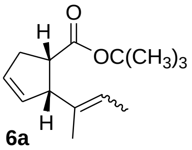
O
H
OC(CH3)3
H

6a

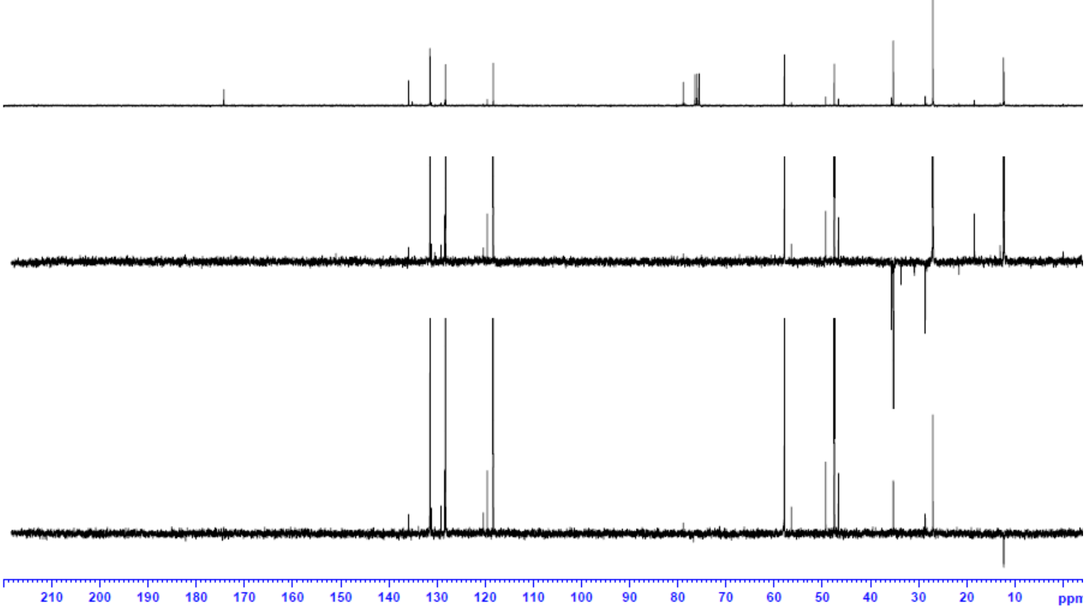
174.31
135.97
131.52
128.30
118.39
78.91
76.48
76.05
75.63
57.92
47.57
35.30
27.09
12.44
12.34
210
200
190
180
170
160
150
140
130
120
110
100
90
80
70
60
50
40
30
20
10
ppm

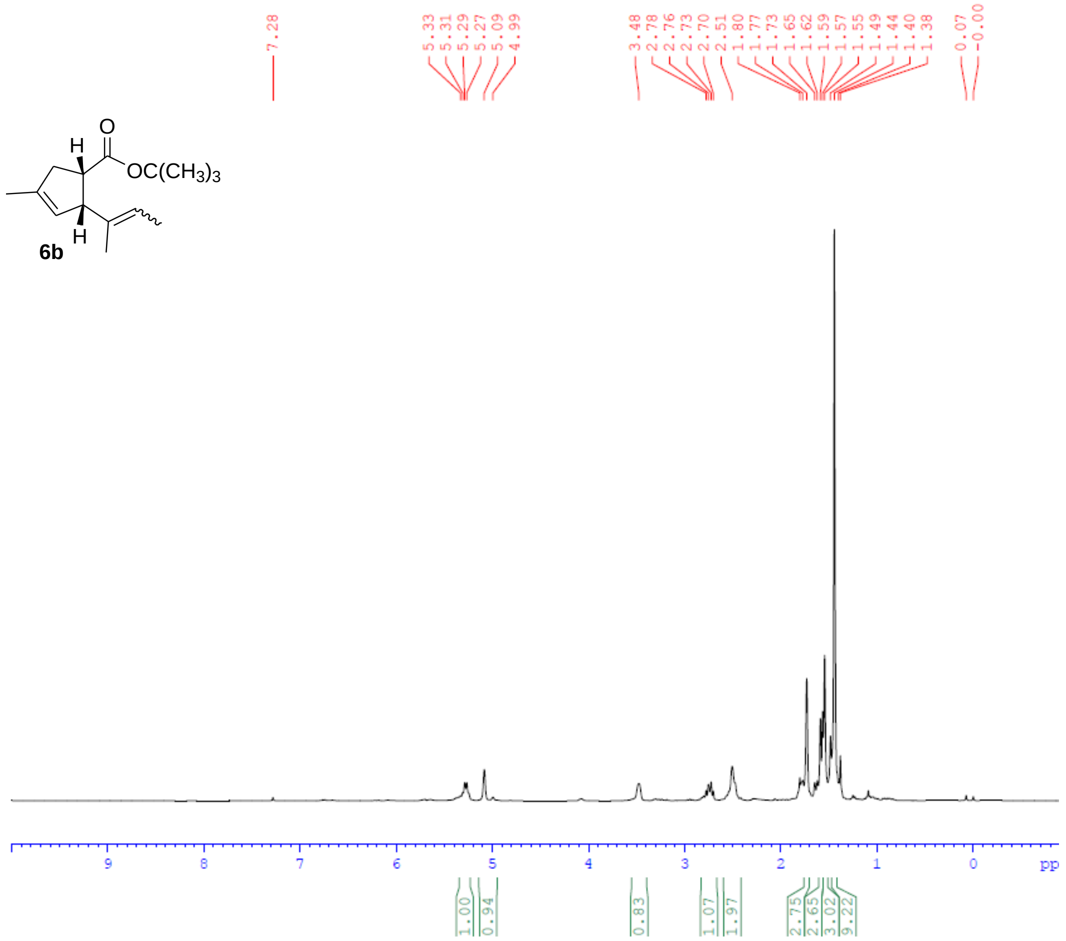

7.28
5.33
5.31
5.29
5.27
5.09
4.99
3.48
2.78
2.76
2.73
2.70
2.51
1.80
1.77
1.73
1.65
1.62
1.59
1.57
1.55
1.49
1.44
1.40
1.38
0.07
-0.00
O
H
OC(CH3)3
H
6b
9
8
7
6
5
4
3
2
1
0
pp
1.00
0.94
0.83
1.07
1.97
2.75
2.65
3.02
9.22

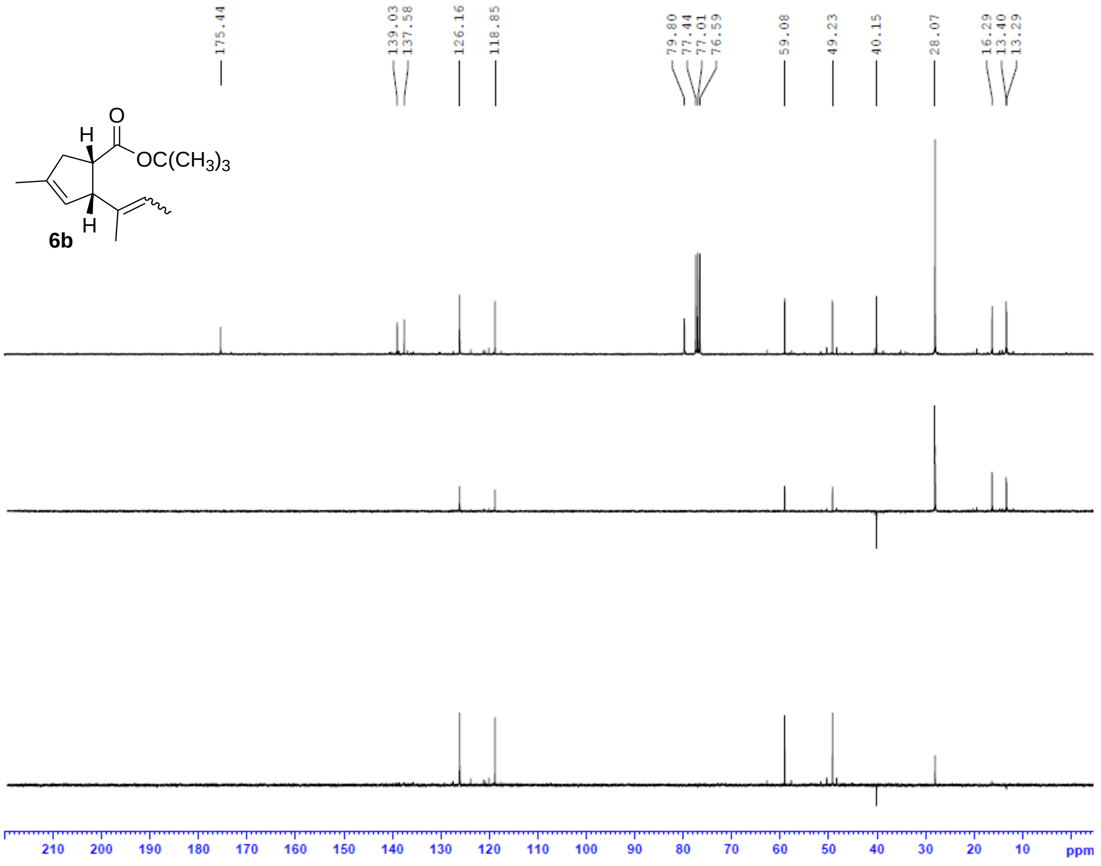

175.44
139.03
137.58
126.16
118.85
79.80
77.44
77.01
76.59
59.08
49.23
40.15
28.07
16.29
13.40
13.29
O
H
OC(CH3)3
H
6b
210
200
190
180
170
160
150
140
130
120
110
100
90
80
70
60
50
40
30
20
10
ppm

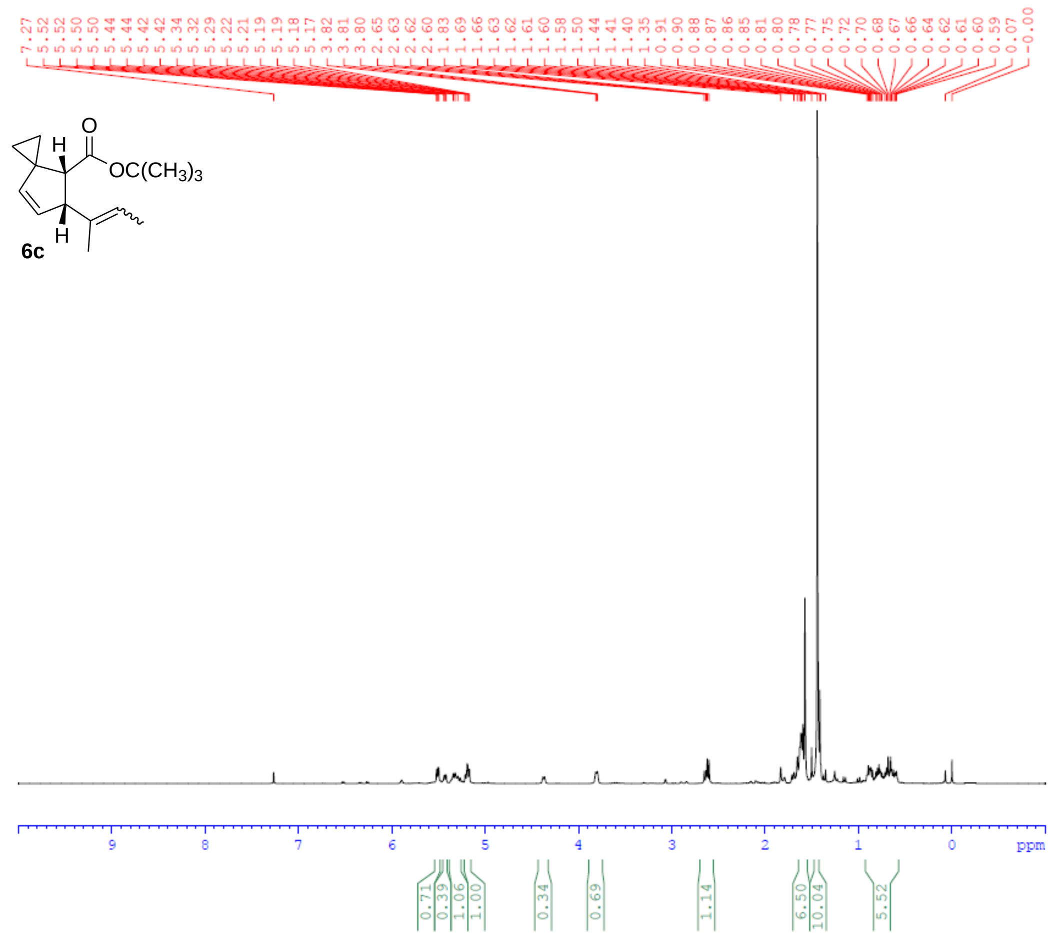

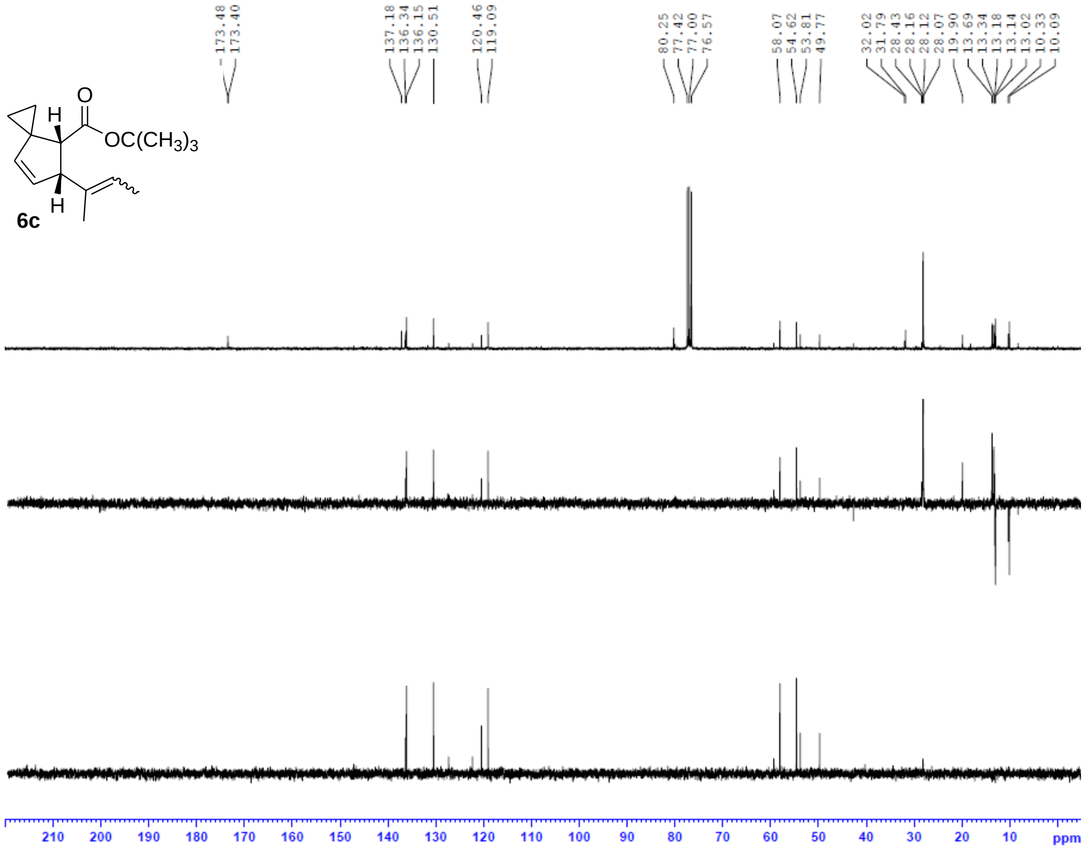

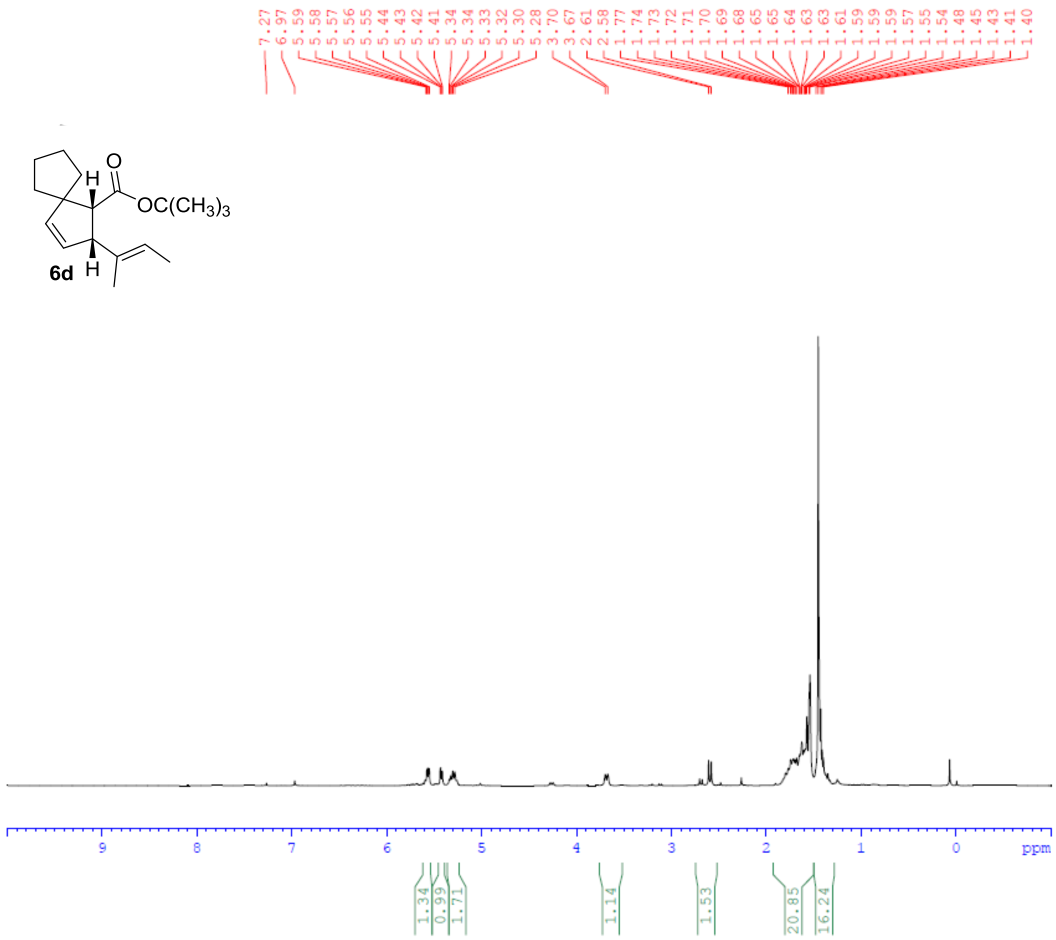
7.27
6.97
5.59
5.58
5.57
5.56
5.55
5.44
5.43
5.42
5.41
5.34
5.34
5.33
5.32
5.30
5.28
3.70
3.67
2.61
2.58
1.77
1.74
1.73
1.72
1.71
1.70
1.69
1.68
1.65
1.65
1.64
1.63
1.63
1.61
1.59
1.59
1.59
1.57
1.55
1.54
1.48
1.45
1.43
1.41
1.40
H
O
OC(CH3)3
H
6d
9
8
7
6
5
4
3
2
1
0
ppm
1.34
0.99
1.71
1.14
1.53
20.85
16.24

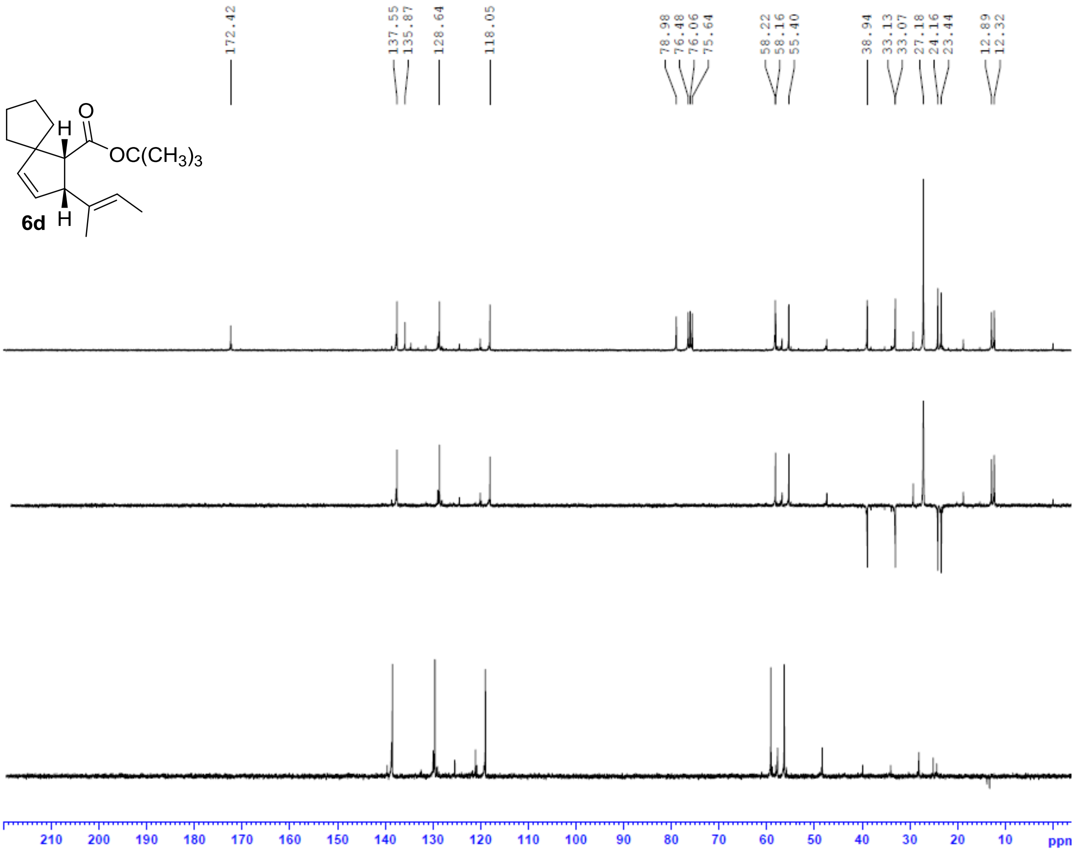
172.42
137.55
135.87
128.64
118.05
78.98
76.48
76.06
75.64
58.22
58.16
55.40
38.94
33.13
33.07
27.18
24.16
23.44
12.89
12.32
H
O
OC(CH3)3
H
6d
210
200
190
180
170
160
150
140
130
120
110
100
90
80
70
60
50
40
30
20
10
ppm

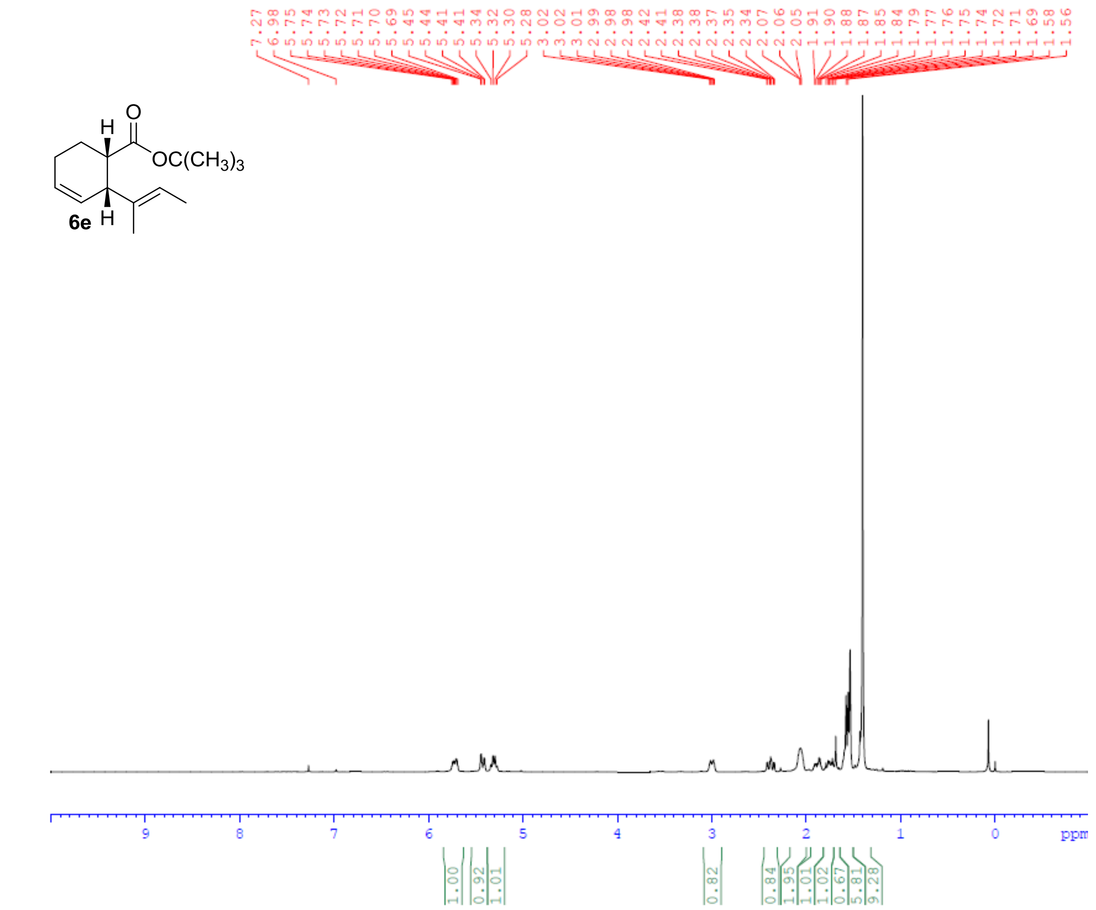
7.27
6.98
5.75
5.74
5.73
5.72
5.71
5.70
5.69
5.45
5.44
5.41
5.41
5.34
5.32
5.30
5.28
3.02
3.02
3.01
2.99
2.98
2.98
2.42
2.41
2.38
2.38
2.37
2.35
2.34
2.07
2.06
2.05
1.91
1.90
1.88
1.87
1.85
1.84
1.79
1.77
1.76
1.75
1.74
1.72
1.71
1.69
1.58
1.56
H
O
OC(CH3)3
6e
H
ppm
1.00
0.92
1.01
0.82
0.84
1.95
1.01
1.02
0.67
5.81
9.28

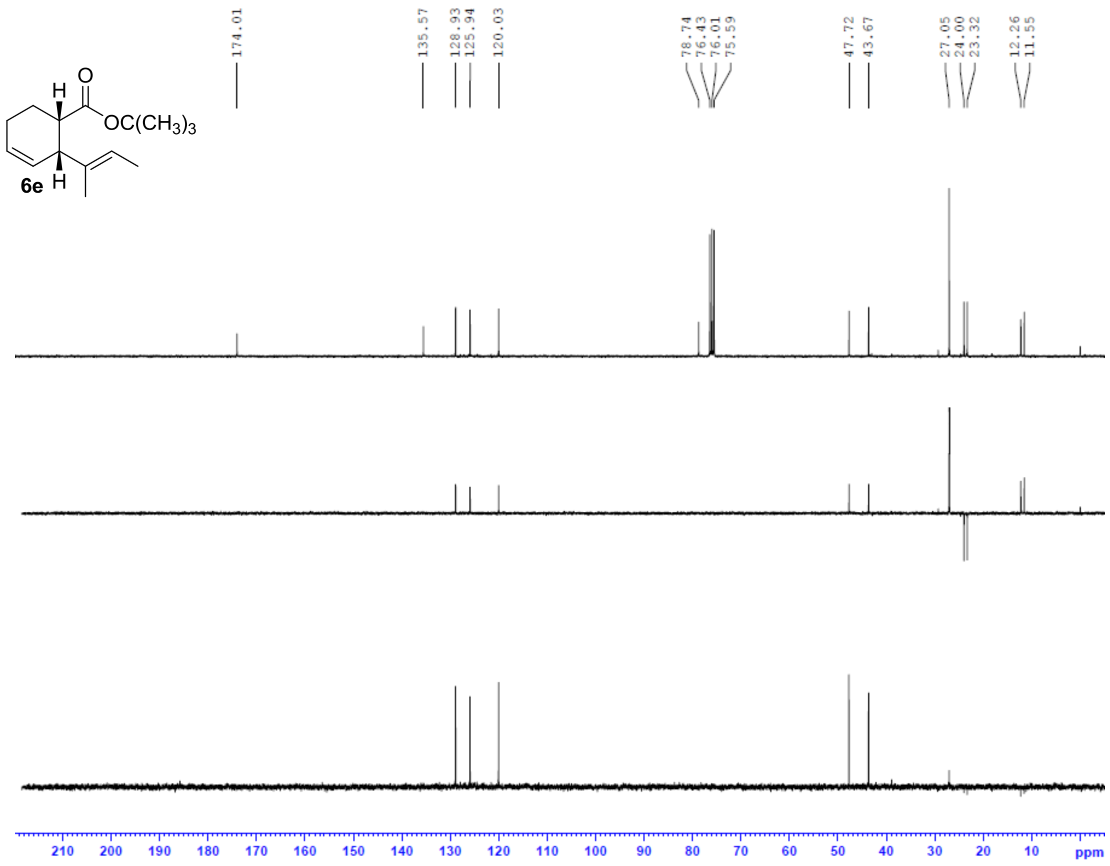
174.01
135.57
128.93
125.94
120.03
78.74
76.43
76.01
75.59
47.72
43.67
27.05
24.00
23.32
12.26
11.55
H
O
OC(CH3)3
6e
H
ppm

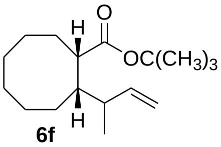
O
H
OC(CH3)3
H

6f

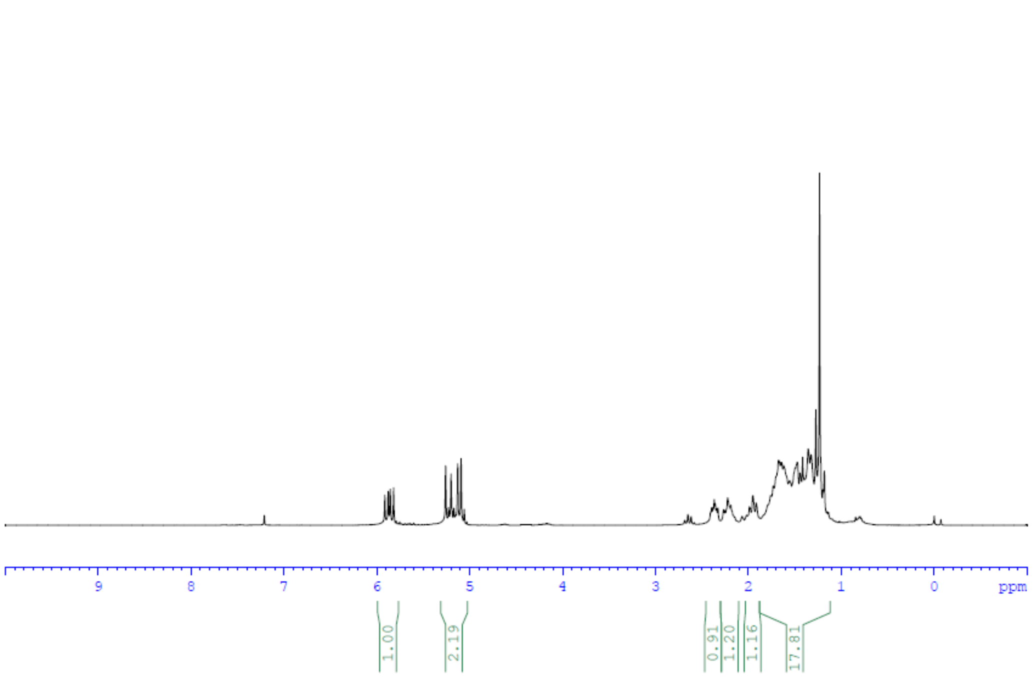
7.21
5.91
5.88
5.86
5.82
5.26
5.23
5.20
5.17
5.15
5.13
5.09
5.06
2.69
2.65
2.62
2.58
2.41
2.40
2.38
2.37
2.36
2.34
2.33
2.27
2.25
2.22
2.19
2.18
1.99
1.98
1.95
1.92
1.68
1.65
1.64
1.62
1.50
1.48
1.44
1.42
1.36
1.33
1.27
9 8 7 6 5 4 3 2 1 0 ppm
1.00
2.19
0.91
1.20
1.16
17.81

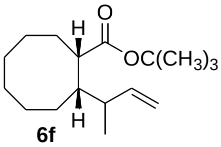
O
H
OC(CH3)3
H

6f

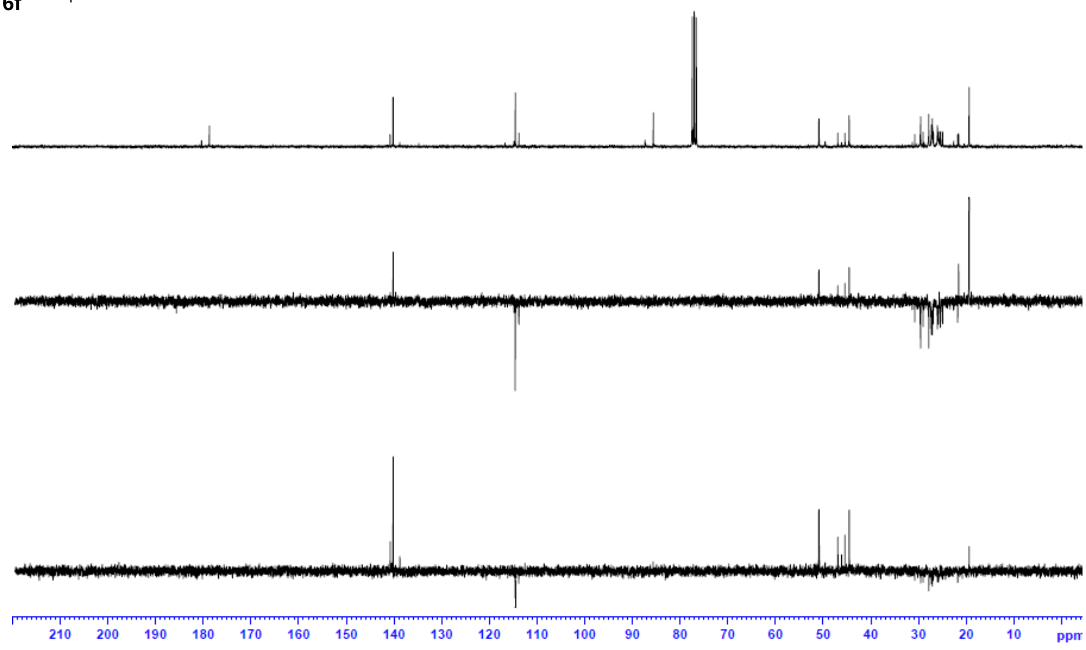
180.35
178.72
140.84
140.21
114.57
113.82
87.38
85.63
50.90
49.61
46.94
46.14
45.45
44.56
30.79
29.66
29.57
29.06
27.86
27.30
27.12
26.91
26.04
25.84
25.68
25.43
25.35
24.97
21.81
21.61
19.41
210 200 190 180 170 160 150 140 130 120 110 100 90 80 70 60 50 40 30 20 10 ppm

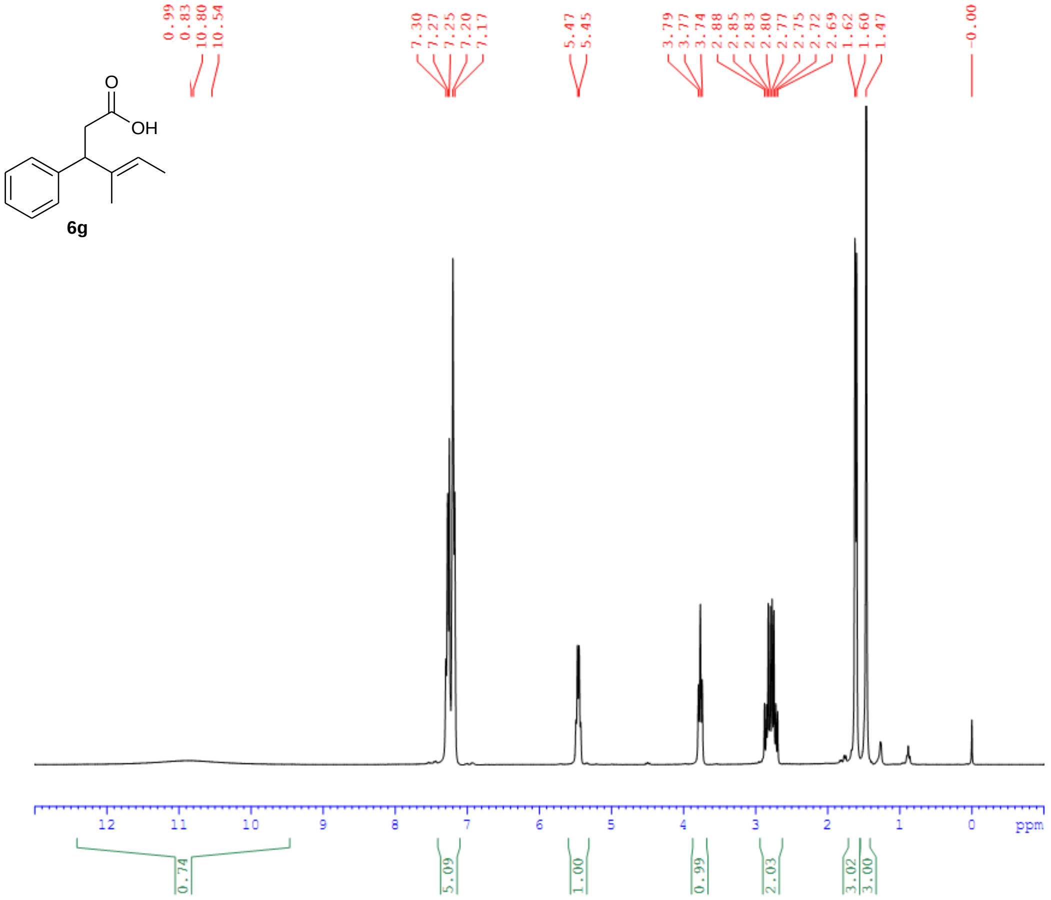
0.99
0.83
10.80
10.54
7.30
7.27
7.25
7.20
7.17
5.47
5.45
3.79
3.77
3.74
2.88
2.85
2.83
2.80
2.77
2.75
2.72
2.69
1.62
1.60
1.47
-0.00
O
OH
6g
ppm
0.74
5.09
1.00
0.99
2.03
3.02
3.00

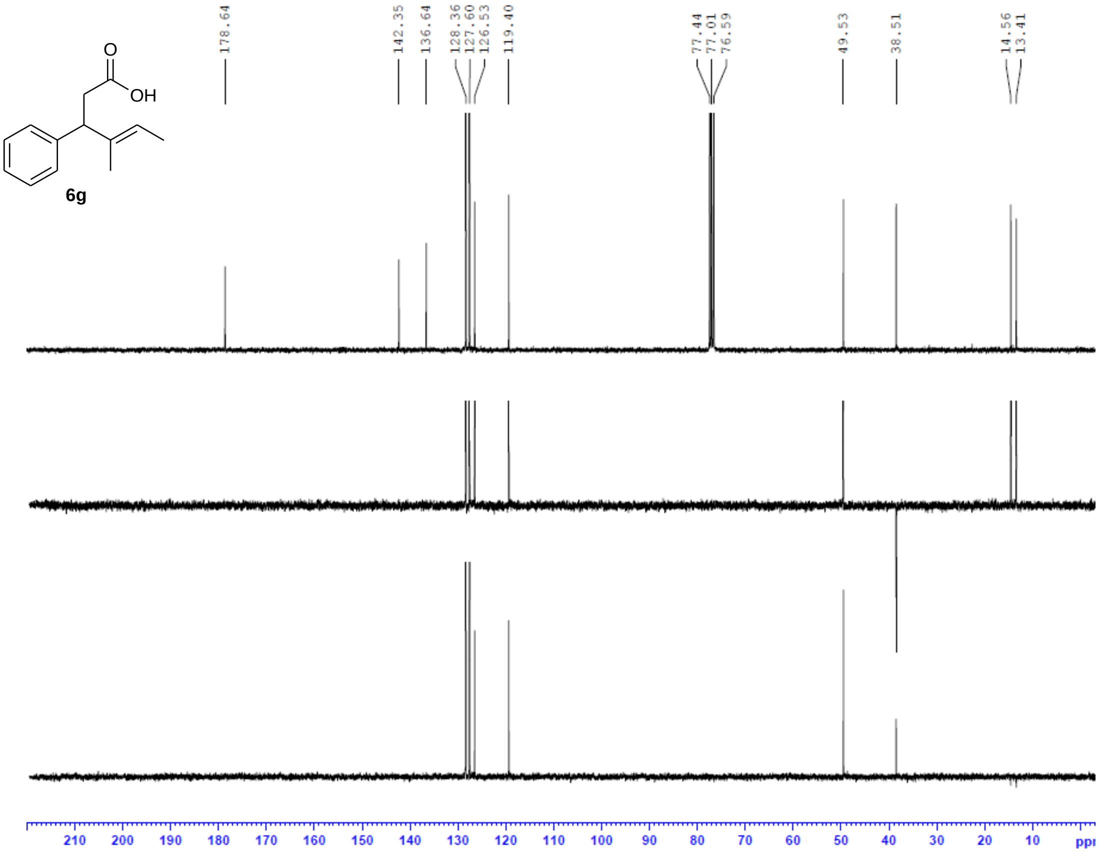
178.64
142.35
136.64
128.36
127.60
126.53
119.40
77.44
77.01
76.59
49.53
38.51
14.56
13.41
O
OH
6g

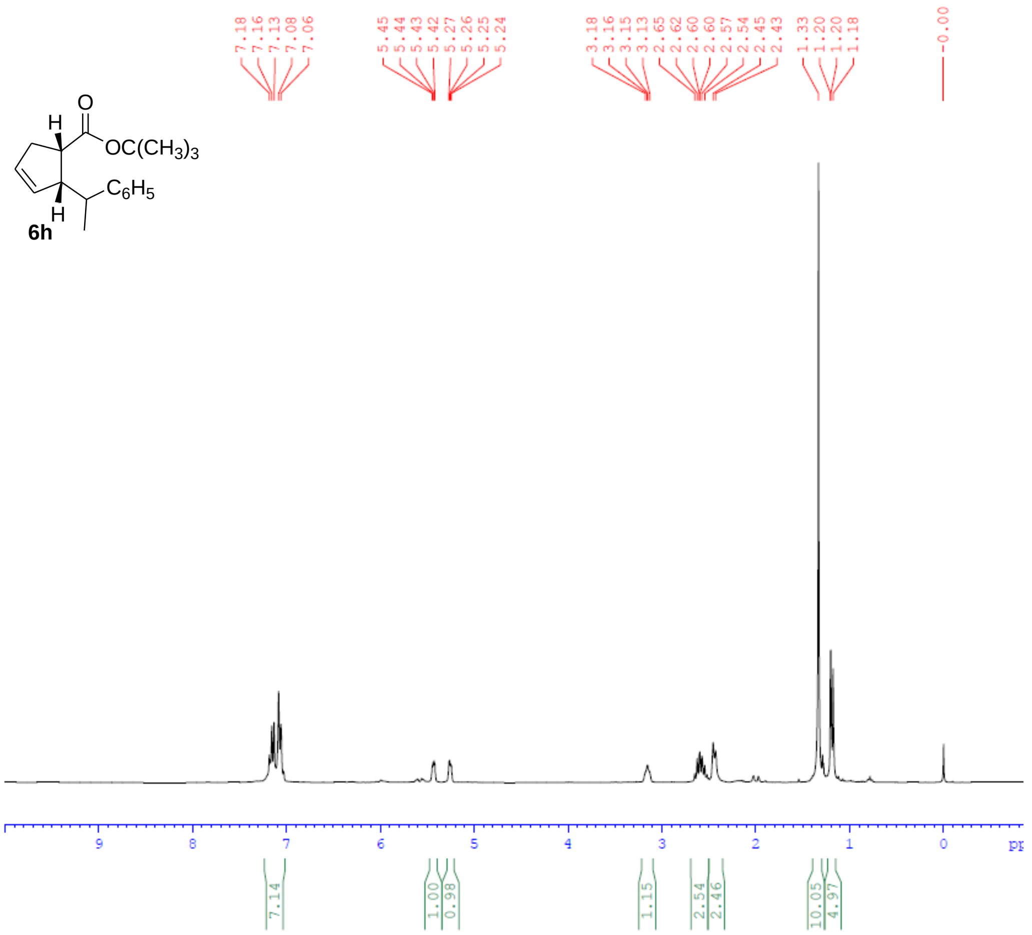

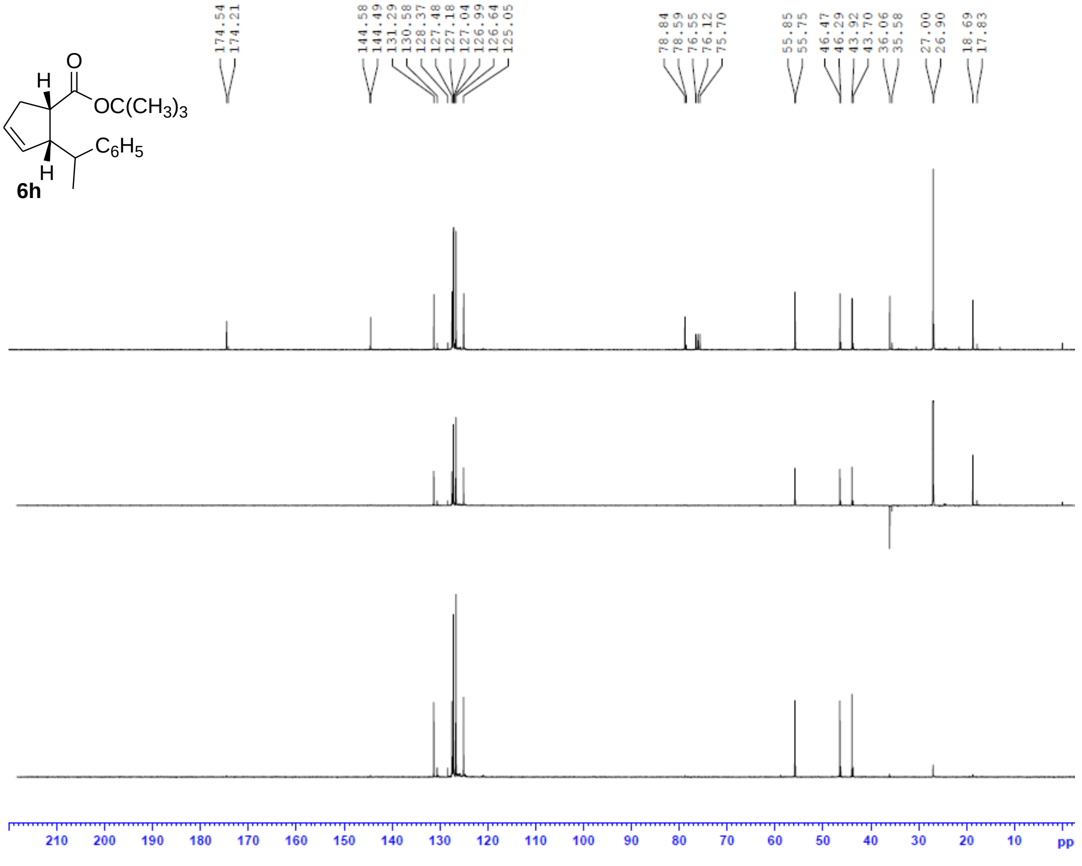

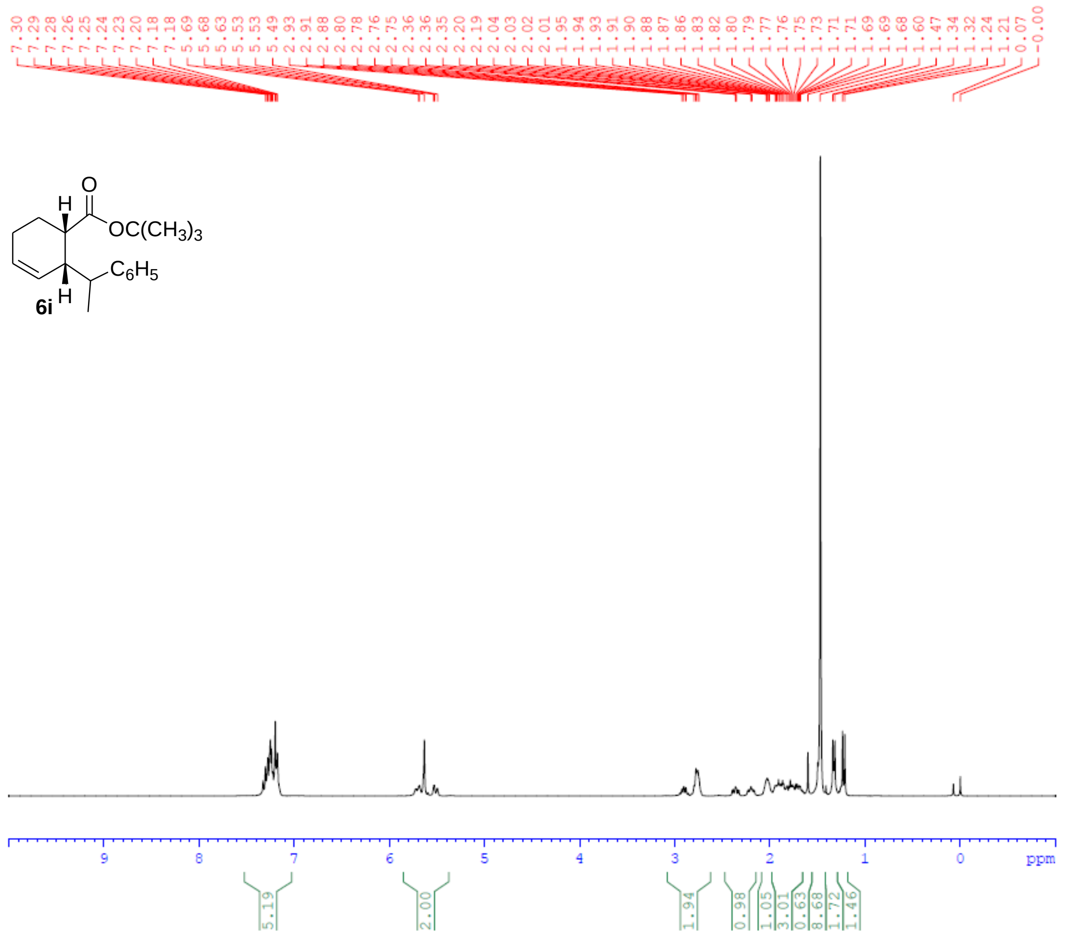

7.30 7.29 7.28 7.26 7.25 7.24 7.23 7.20 7.18 5.69 5.68 5.63 5.53 5.53 5.49 2.93 2.91 2.88 2.80 2.78 2.76 2.75 2.36 2.36 2.35 2.20 2.19 2.04 2.03 2.02 2.01 1.95 1.94 1.93 1.91 1.90 1.88 1.87 1.86 1.83 1.82 1.80 1.79 1.77 1.76 1.75 1.73 1.71 1.71 1.69 1.69 1.68 1.60 1.47 1.34 1.32 1.24 1.21 0.07 -0.00
H
O
OC(CH3)3
C6H5
6i
H
9 8 7 6 5 4 3 2 1 0 ppm
5.19 2.00 1.94 0.98 1.05 3.01 0.63 8.68 1.72 1.46

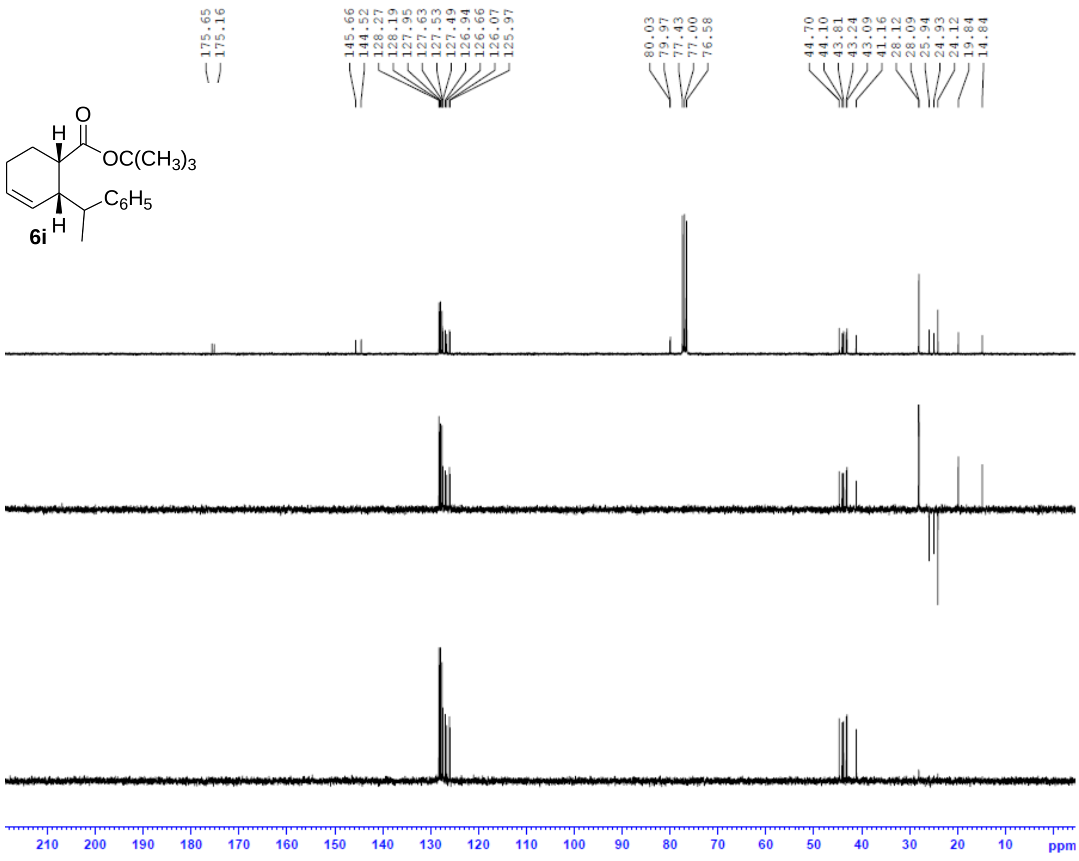

175.65 175.16 145.66 144.52 128.27 128.19 127.95 127.63 127.53 127.49 126.94 126.66 126.07 125.97 80.03 79.97 77.43 77.00 76.58 44.70 44.10 43.81 43.24 43.09 41.16 28.12 28.09 25.94 24.93 24.12 19.84 14.84
H
O
OC(CH3)3
C6H5
6i
H
210 200 190 180 170 160 150 140 130 120 110 100 90 80 70 60 50 40 30 20 10 ppm

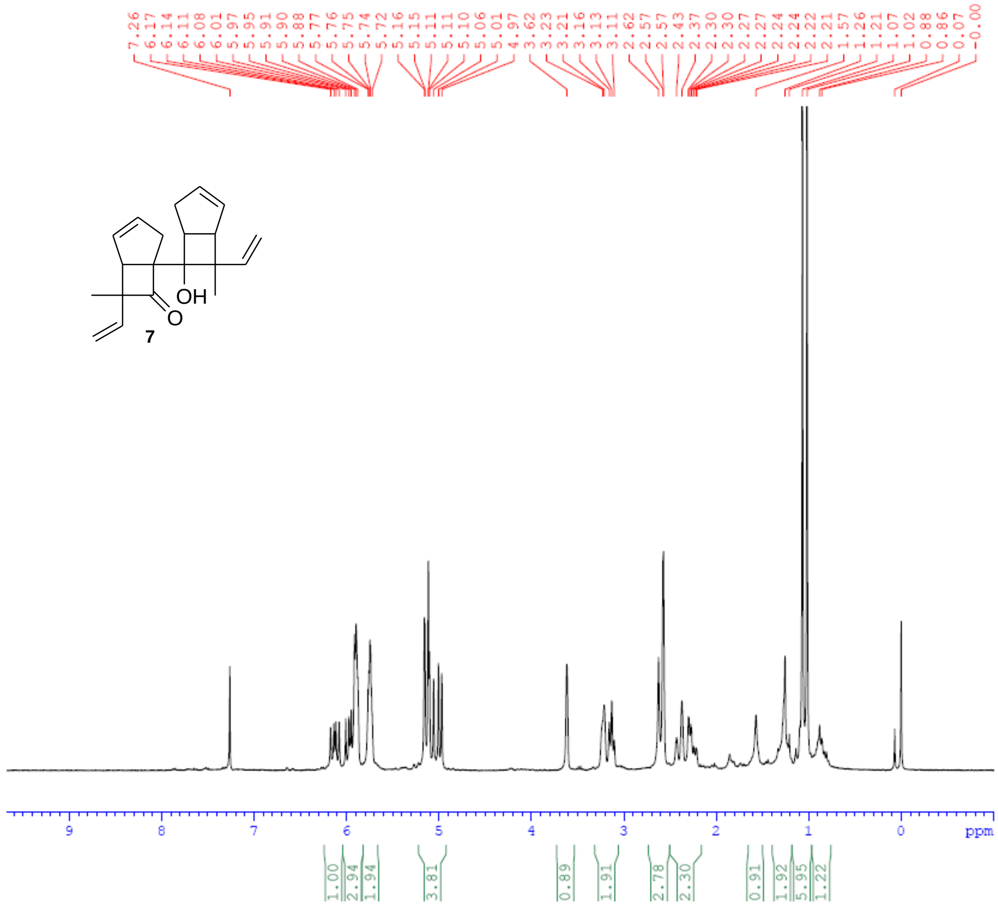
7.26 6.17 6.14 6.11 6.08 6.01 5.97 5.95 5.91 5.90 5.88 5.77 5.76 5.75 5.74 5.72 5.16 5.15 5.11 5.11 5.10 5.06 5.01 4.97 3.62 3.23 3.21 3.16 3.13 3.11 2.62 2.57 2.57 2.43 2.37 2.30 2.30 2.27 2.27 2.24 2.24 2.22 2.21 1.57 1.26 1.21 1.07 1.02 0.88 0.86 0.07 -0.00
OH
O
7
9 8 7 6 5 4 3 2 1 0 ppm
1.00 2.94 1.94 3.81 0.89 1.91 2.78 2.30 0.91 1.92 5.95 1.22

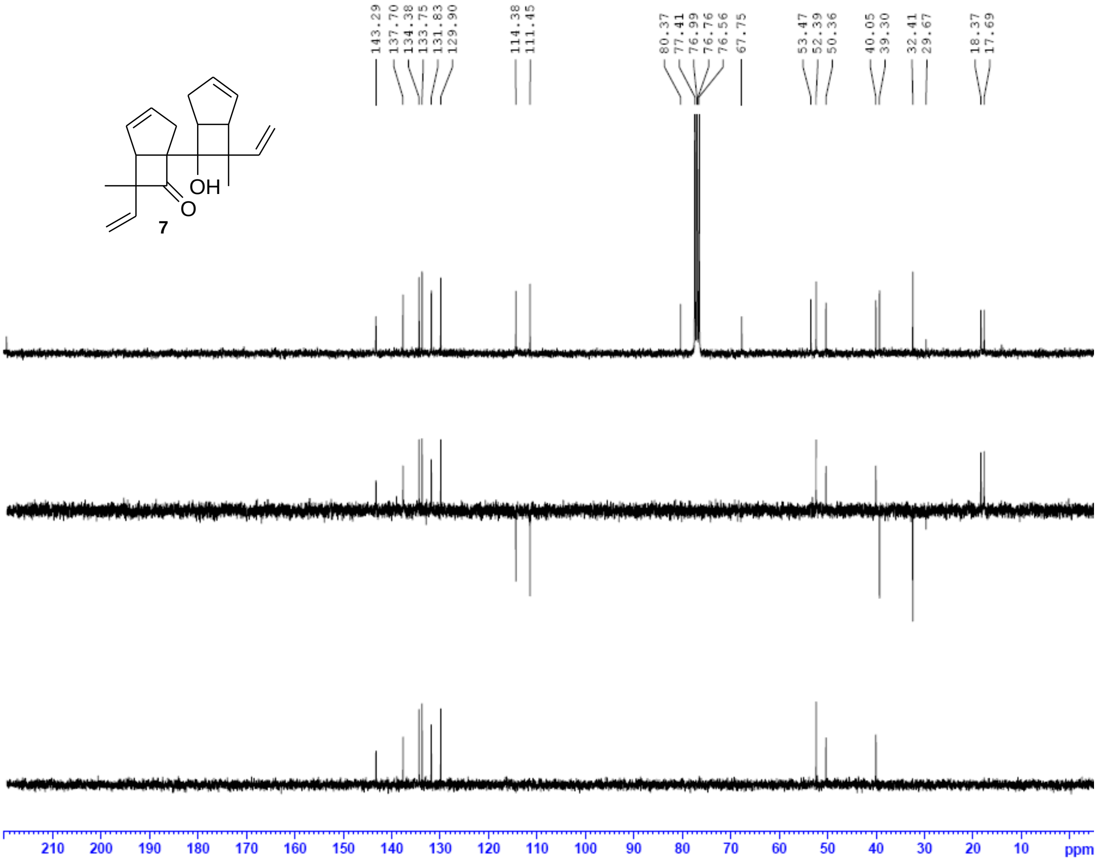
143.29 137.70 134.38 133.75 131.83 129.90 114.38 111.45 80.37 77.41 76.99 76.76 76.56 67.75 53.47 52.39 50.36 40.05 39.30 32.41 29.67 18.37 17.69
OH
O
7
210 200 190 180 170 160 150 140 130 120 110 100 90 80 70 60 50 40 30 20 10 ppm
